# Distinctive Nucleic Acid Recognition by Lysine-Embedded Phenanthridine Peptides

**DOI:** 10.3390/ijms25094866

**Published:** 2024-04-29

**Authors:** Josipa Matić, Patryciusz Piotrowski, Lucija Vrban, Renata Kobetić, Robert Vianello, Ivona Jurić, Ivana Fabijanić, Margareta Pernar Kovač, Anamaria Brozovic, Ivo Piantanida, Carsten Schmuck, Marijana Radić Stojković

**Affiliations:** 1 Laboratory for Biomolecular Interactions and Spectroscopy, Division of Organic Chemistry and Biochemistry, Ruđer Bošković Institute, Bijenička Cesta 54, 10000 Zagreb, Croatia; josipa.matic@gmail.com (J.M.); renata.kobetic@irb.hr (R.K.); juric.ivona@student.pmf.hr (I.J.); i.s.fabijanic@gmail.com (I.F.); ivo.piantanida@irb.hr (I.P.); 2 Institute for Organic Chemistry, University of Duisburg-Essen, Universitätsstrasse 7, 45141 Essen, Germany; p.piotrowski@gmx.net (P.P.);; 3 Laboratory for the Computational Design and Synthesis of Functional Materials, Division of Organic Chemistry and Biochemistry, Ruđer Bošković Institute, Bijenička Cesta 54, 10000 Zagreb, Croatia; lvrban@irb.hr (L.V.); robert.vianello@irb.hr (R.V.); 4 Division of Molecular Biology, Ruđer Bošković Institute, Bijenička Cesta 54, 10000 Zagreb, Croatia; margareta.pernar@irb.hr (M.P.K.); anamaria.brozovic@irb.hr (A.B.)

**Keywords:** DNA/RNA recognition, phenanthridine peptides, spectrophotometric AT- and GC-base pair probe, molecular dynamics simulations

## Abstract

Three new phenanthridine peptide derivatives (**19**, **22**, and **23**) were synthesized to explore their potential as spectrophotometric probes for DNA and RNA. UV/Vis and circular dichroism (CD) spectra, mass spectroscopy, and computational analysis confirmed the presence of intramolecular interactions in all three compounds. Computational analysis revealed that compounds alternate between bent and open conformations, highlighting the latter’s crucial influence on successful polynucleotide recognition. Substituting one glycine with lysine in two regioisomers (**22**, **23**) resulted in stronger binding interactions with DNA and RNA than for a compound containing two glycines (**19**), thus emphasizing the importance of lysine. The regioisomer with lysine closer to the phenanthridine ring (**23**) exhibited a dual and selective fluorimetric response with non-alternating AT and ATT polynucleotides and induction of triplex formation from the AT duplex. The best binding constant (K) with a value of 2.5 × 10^7^ M^−1^ was obtained for the interaction with AT and ATT polynucleotides. Furthermore, apart from distinguishing between different types of ds-DNA and ds-RNA, the same compound could recognize GC-rich DNA through distinct induced CD signals.

## 1. Introduction

Spectrophotometric probes targeting specific polynucleotide sequences are indispensable tools in medicinal chemistry and biochemistry. They enable visualization and quantification of polynucleotides in cells and tissues and provide insight into different biological mechanisms involving these macromolecules [1,2,3]. Besides specific binding, probes can show sensitivity to the surrounding environment, enabling, for example, the monitoring of pH value in the cellular environment [4,5,6]. An important characteristic of the small-molecule probes is their tunability, i.e., the ability to modify cell permeability, biocompatibility, specificity, or optical properties of the probe by simple synthetic modifications [7].

Among various fluorescent molecules described in the literature, phenanthridine dyes have received special attention [8,9,10]. One of the most prominent members of the phenanthridine family is ethidium bromide (EB), characteristic of its bright fluorescence that arises upon intercalation of EB between DNA base pairs. However, potential mutagenicity, low specificity for the polynucleotide sequences, and low quantum yield hinder its application [11,12]. Nonetheless, it was shown that modifications and functional groups introduced to the phenanthridine core significantly influence the binding specificity and fluorescence properties [12,13,14,15,16,17]. 

In our previous work, we described two phenanthridine probes functionalized with a guanidinocarbonylpyrrole (GCP) moiety, connected through different peptide linkers [18]. Phenanthridine was employed as a tuneable fluorescent reporter, while the GCP unit modulated the binding specificity. However, only one phenanthridine–GCP hybrid showed selectivity towards double-stranded (ds) polynucleotides and distinguished between the ds-DNA and ds-RNA by different spectroscopic responses. It turned out to be a result of the probes’ conformation, which had a decisive influence on their interaction. The linker length and the pH of the probe environment affected the conformation.

**Figure 1:**
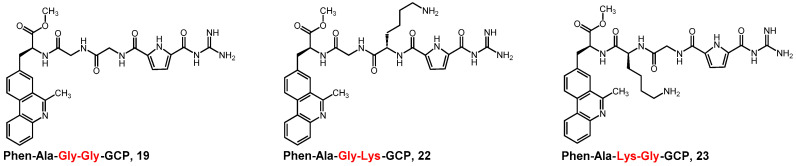
Structures of studied compounds **19**, **22**, and **23**.

In this work, we aimed to explore the tuneable nature of phenanthridine–GCP probes in more detail. Lysine is an essential amino acid crucial for protein synthesis and plays a key role in many metabolic and signaling processes [19]. From a chemical point of view, lysine contains two amino groups and one carboxylic acid group, which allows for increased reactivity and an additional conjugation site. It makes it an interesting unit for synthesizing lysine derivatives for various medical and supramolecular applications [20,21]. Under physiological pH, the positively charged amine sidechain can enable electrostatic interactions with DNA phosphates and hydrogen bonds within DNA grooves. Because of all these reasons, we decided to enrich the previous series of fluorescent probes with lysine. Thus, three new fluorescent probes were prepared, including Phen-Ala-Gly-Gly-GCP (**19**), Phen-Ala-Gly-Lys-GCP (**22**), and Phen-Ala-Lys-Gly-GCP (**23**), with phenanthridine coupled to GCP over flexible tripeptide linkers (Figure 1). Two of the linkers incorporated lysine amino acids with different positions in the linker, while the third contained glycine to assess the influence of lysine on interactions with nucleic acid structures. The literature indicates that the phenanthridine heterocyclic nitrogen undergoes protonation in an acidic medium (p*K*_a_ ≈ 6) [8], implying that our phenanthridine probes would exist in a protonated state at pH 5.0. In addition, attaching the guanidinium group via an acetyl group to the pyrrole decreases the basicity of the former. An illustrative example is acetylguanidine with a p*K*_a_ of 8.33, representing a drop of over 5 p*K*_a_ units from 13.71 measured in pure guanidine [22]. Yet, this still allows this moiety to assume a cationic form under both experimental conditions. Further, the p*K*_a_ value of the aminoalkyl unit of lysine is around 10.5 [23]. Thus, it can be assumed that **19** possesses two positive charges at pH 5.0, while at pH 7.0, the molecule is monoprotonated. Accordingly, **22** and **23** possess two positive charges at pH 7.0 and three positive charges at pH 5.0.

In the present work, we studied interactions of new phenanthridine–GCP conjugate probes with various ds-polynucleotides and a triple-stranded (ts) polynucleotide dAdTdT. New modifications of the peptide linker and additional interacting units significantly altered the conjugates’ binding mode and specificity and opened possibilities for new applications. 

## 2. Results and Discussion

### 2.1. Synthesis of Phenanthridine–Guanidinocarbonylpyrrole Tetrapeptides

**Scheme 1:**
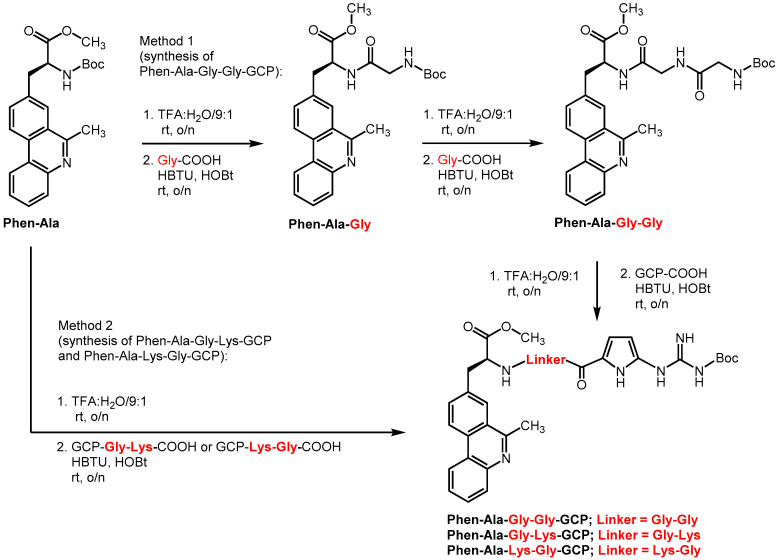
Synthesis of **19**, **22**, and **23**.

Synthesis of studied phenanthridine–guanidinocarbonylpyrrole (Phen-GCP) tetrapeptides started from phenanthridine amino acid (Phen-Ala) [18,24], via two pathways as follows: (1) functionalizing Phen-Ala with a Gly-Gly linker and subsequent amide coupling with GCP–carboxylic acid and (2) amide coupling of Phen-Ala with a preconstructed guanidinocarbonylpyrrole building block (Gly-Lys-GCP or Lys-Gly-GCP). We prepared compound Phen-Ala-Gly-Gly-GCP (**19**) and compounds Phen-Ala-Gly-Lys-GCP (**22**) and Phen-Ala-Lys-Gly-GCP (**23**) following pathways 1 and 2, respectively (Scheme 1). Syntheses of building blocks Phen-Ala and GCP-carboxylic acid were described previously [18].

### 2.2. Spectroscopic Characterization of ***19***, ***22***, and ***23*** in Aqueous Solutions

#### 2.2.1. UV/Vis, Fluorescence, and Circular Dichroism (CD) Spectra 

Compounds **22** and **23** were soluble in pure water (*c* = 1 × 10^−3^ mol dm^−3^), while compound **19** was dissolved in DMSO (*c* = 5 × 10^−3^ mol dm^−3^) and water (*c* = 2 × 10^−3^ mol dm^−3^).

**Figure 2:**
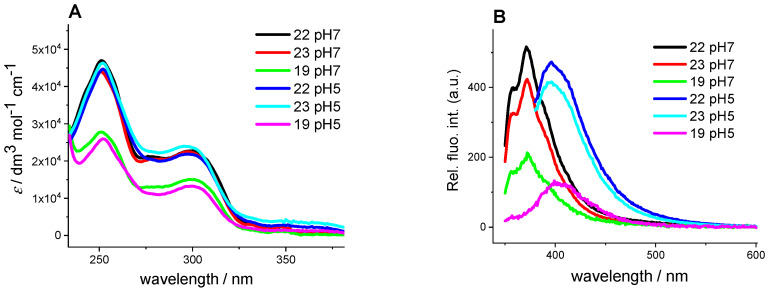
(**A**) Absorption spectra of **19**, **22**, and **23** at *c* = 5 × 10^−6^ mol dm^−3^. (**B**) Fluorescence spectra of **19**, **22**, and **23** at concentration, *c* = 1.5 × 10^−6^ mol dm^−3^ at λ_exc_ = 251 nm, pH 7.0 and 5.0, Na–cacodylate buffer, *I* = 0.05 mol dm^−3^.

The studied compounds remained stable in buffered solutions for several days, while their absorbancies at pH 7.0 and pH 5.0 are proportional to their concentrations up to *c* = 2.5 × 10^−5^ mol dm^−3^ (Figure 2 and Figures S1 and S2 in the Supporting Information (SI)). Absorption maxima and determined molar extinction coefficients (ε) are shown in Table 1. Compound **19** was highly hygroscopic, which obstructed its accurate weighing. The stock solution concentration was determined using the extinction coefficient of tetrapeptide **23**. 

The absorption spectra of **19**, **22**, and **23** exhibited small changes upon a temperature rise to 95 °C at the 251 and 252 nm maxima at both pHs. The UV/Vis spectra exhibited great reproducibility upon cooling back to 25 °C. However, all three compounds, especially **22**, showed an increase in absorbance at 300 nm maximum (~12–15% at pH 7.0 and up to 10% at pH 5.0), indicating potential molecular stacking (see Figure S3 in SI). The linearity observed in UV/Vis and CD measurements with increasing compound concentrations up to 2.5 × 10^−5^ mol dm^−3^ suggests the absence of intermolecular interactions or the formation of intermolecular dimers (see Figures S1, S2 and S5 in SI).

The absorption maxima of **22** and **23** were remarkably similar to the reference compounds **Phen-Ala**, **Phen-Ala-Gly**, and **GCP** [24] (Table 1, Scheme 1) under neutral and weakly acidic conditions. However, a hypochromic effect was observed by comparing the absorption spectra of **22** and **23** (at both pHs) with the corresponding referent compounds **Phen-Ala**, **Phen-Ala-Gly**, and **GCP** (Table 1, see Figure S3 in SI). This observation, in line with the previously mentioned temperature dependence, strongly supports the presence of intramolecular stacking interactions between the phenanthridine and pyrrole chromophores. A similar phenomenon was noted in the first generation of phenanthridine–GCP conjugates lacking lysine [18]. Furthermore, the molar absorptivities (Table 1) of **22** and **23** at both pHs were smaller than the sums of the molar absorptivities of corresponding references (**Phen-Ala** or **Phen-Ala-Gly** and **GCP**). This suggests the influence of intramolecular interactions. This assumption was additionally confirmed through mass spectroscopy and computational analysis.

The fluorescence intensities of the studied compounds in buffered solutions (sodium cacodylate buffer, *I* = 0.05 mol dm^−3^, pH 7.0) are directly proportional to their concentrations up to *c* = 2 × 10^−6^ mol dm^−3^ (Figure S4 in SI). Fluorimetric measurements were conducted within a spectral range where the emission and excitation spectra did not overlap. The pH of the solution influenced the values of the maximum fluorescence wavelengths of all compounds (Δλ = 20–30 nm, Table 1). This shift is likely attributable to the protonation of the phenanthridine fluorophore.

**Figure 3:**
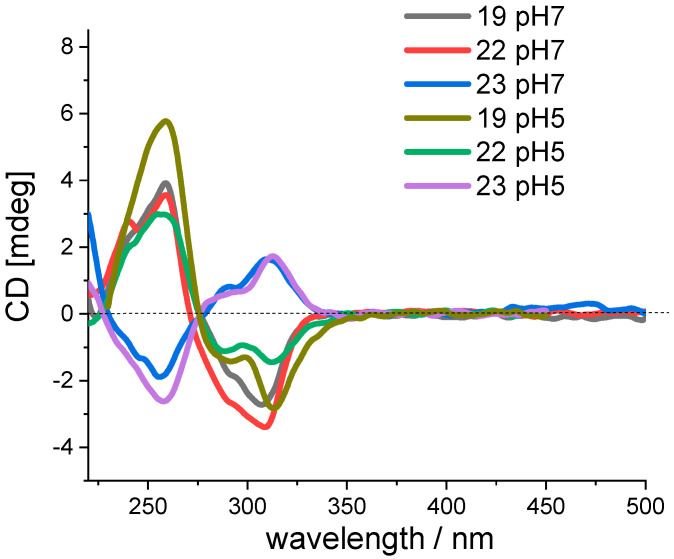
CD spectra of **19**, **22**, and **22** (*c* = 1.5 × 10^−5^ mol dm^−3^) at pH 7.0 and 5.0, sodium cacodylate buffer, *I* = 0.05 mol dm^−3^.

The CD spectra of **19** and **22** at both pH values (Figure 3) were characterized by a dominant negative and a positive CD band of similar intensities, located at 309 nm and 260 nm, respectively. Tetrapeptides **19** and **22** had an additional CD band at 288 nm at pH 5.0. Compound **23** was also characterized by bisignate CD bands, both in neutral and weakly acid conditions, of similar intensities (centered around 257 nm and 310 nm, respectively, with an additional CD band at 290 nm) but opposite signs compared with **19** and **22** intrinsic CD signals. These wavelength values match the phenanthridine and pyrrole absorption maxima in compounds **19**, **22**, and **23**. The aforementioned bisignate CD signals suggest the formation of intramolecular or intermolecular dimers [25,26,27].

#### 2.2.2. Mass Spectra (MS)

Even though electrospray ionization (**ESI**) is a mild technique and the non-covalent interactions such as hydrogen bonding or electrostatic interactions could be transferred into the gas phase of the instrument, we do not neglect the fact that various properties of the non-covalent bonds—strength, directionality, and geometry—may significantly change during the transition from a condensed phase to high vacuum. Any interaction that competes with the solvent could, upon evaporation, either increase in its strength (like hydrogen bonds or electrostatic interactions) or lower in its strength (like hydrophobic interactions). Thus, we suggest that the observed results in the gas phase could be related to the aggregation tendency of the presently studied compounds [28,29,30,31].

ESI MS analysis of compounds **19** (Figure S41a,b), **22** (Figure S41c,d), and **23** (Figure S41e–g) dissolved in water and D_2_O showed the presence of multiply charged species in the gas phase analog for the pH 7.0 conditions in the condensed phase. The signals at *m*/*z* 587.2 and 294.1 assigned as the single/double-charged adducts (hydrogen) were observed in the spectra of compound **19**. Compounds **22** and **23** are isomers having identical signals observed at *m*/*z* 658.3 (single-charged H-adduct), 329.7 (double-charged H-adduct), and 220.1 (triple-charged H-adduct). 

The aqueous solutions of isomers **22** and **23** have the same signals in the gas phase, but with time, the abundance of the signals corresponding to triple-charged species changed. We did not detect the signal for the triple-charged species of compound **22** after one hour. 

All three compounds contain functional groups capable of forming inter- and intra-molecular noncovalent interactions—H-bonds. Interestingly, the H/D exchange rate for two isomers and the assembling differed. Namely, the signals corresponding to the aggregation were observed only for compound **23** (e.g., the signal at *m*/*z* 1337.6 assigned as a dimer [2M+Na]^+^). The H/D exchange of compounds **19** and **22** is faster than that of **23** (Figure S41a–g). For example, following the isotope distribution of the signal at *m*/*z* 329,6 assigned as [M+2H]^2+^ or at *m*/*z* 658,3 assigned as [M+H]^+^ for both isomers during the 24 h, we observed that the distribution matched if the H/D exchange was compared by 1 h of **22** with 24 h of compound **23** (Figure S41c–f). Therefore, we can sum that compound **23** has a slower H/D exchange rate and forms detectable aggregates in the gas phase. 

### 2.3. Study of Interactions of ***19***, ***22***, and ***23*** with ds-DNA and ds-RNA in Aqueous Media

#### 2.3.1. Spectrophotometric Titrations

Calf thymus DNA (ctDNA), comprising 42% GC base pairs and 58% AT base pairs, along with alternating polynucleotides poly(dAdT)_2_ and poly(dGdC)_2_ (featuring a minor groove sterically hindered by guanine amino groups) were selected for examination with double-stranded (ds-) polynucleotides as representatives of the classic B-helix. Poly A-poly U (pApU) was used as an RNA model exhibiting a distinctive A-helical structure with a wide and shallow minor groove and a deep, narrow major groove [32].

Additionally, poly dA-poly dT (pdApdT) was employed, characterized by consecutive AT sequences that create a unique twisted secondary structure. This structure features a minor groove, notably narrower and deeper than the typical B-helix observed in other ds-DNAs [33].

**Figure 4:**
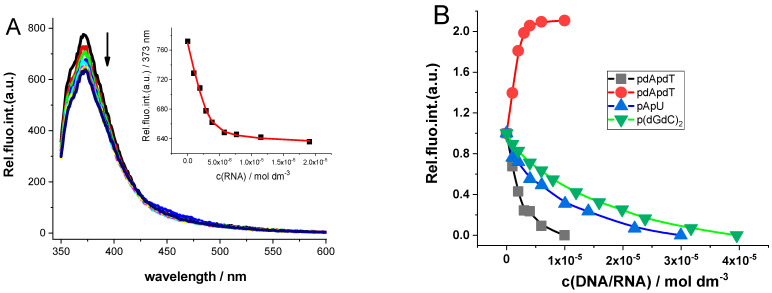
(**A**) Changes in the fluorescence spectrum of **23** (*c* = 1.0 × 10^−6^ mol dm^−3^, λ_exc_ = 300 nm) upon titration with poly A-poly U (*c* = 1.0 × 10^−6^–1.9 × 10^−5^ mol dm^−3^). Inset: Dependence of **23** fluorescence at λ_max_ = 372 nm on *c* (poly A-poly U) at pH 7.0. (**B**) Normalized changes in the fluorescence emission of **23** (*c* = 1.0 × 10^−6^ mol dm^−3^) upon polynucleotide addition (pH 5.0, sodium cacodylate buffer, *I* = 0.05 mol dm^−3^).

The different protonation states of the investigated compounds allowed for the assessment of binding affinities in neutral (pH 7.0) and slightly acidic (pH 5.0) conditions. Polynucleotide addition to the buffered aqueous solution of **19**, **22**, and **23** at pH 7.0 and pH 5.0 resulted in a small or moderate decrease in their fluorescence (Figure 4A and Figures S6–S37 in SI). 

**Figure 5:**
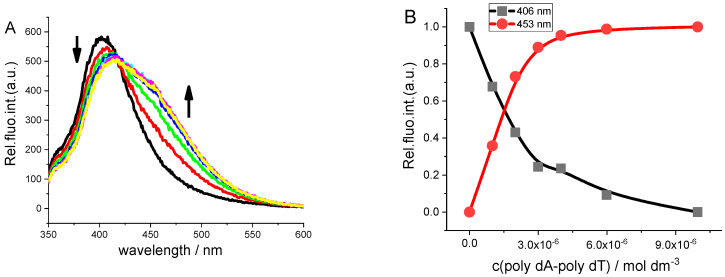
(**A**) Changes in the fluorescence spectrum of **23** (*c* = 1.0 × 10^−6^ mol dm^−3^, λ_exc_ = 300 nm) upon titration with poly dA-poly dT (*c* = 1.0 × 10^−6^–1 × 10^−5^ mol dm^−3^). (**B**) Dependence of **23** absorbance at λ_max_ = 406 and 453 nm on *c* (poly dA-poly dT), at pH 5.0, sodium cacodylate buffer, *I* = 0.05 mol dm^−3^.

Nevertheless, poly dA-poly dT addition to the solution of **23** at pH 5.0 revealed a distinct fluorimetric response, unlike the other polynucleotides tested. Specifically, poly dA-poly dT addition led to the fluorescence quenching of **23** at its maximum emission value of 406 nm and an enhancement in the fluorescence of **23** at the alternate maximum value (453 nm) (Figure 5). This change, coupled with a consistent deviation from the isosbestic point (413–420 nm), suggests the formation of two distinct complexes between **23** and poly dA-poly dT.

A similar dual fluorimetric response, akin to that observed with poly dA-poly dT, was evident during the titration with dAdTdT (ATT) triplex (Figure S37 in SI). We employed additional methods like CD spectroscopy and thermal melting experiments to further elucidate the binding modes of **23** with poly dA-poly dT and ATT triplex. It is noteworthy that compound **23** caused an increase in fluorescence only with consecutive AT base pairs (Figure 4B).

Analysis of the measured data collected with an excess of polynucleotide over a compound using the Scatchard equation [34,35] yielded binding constants *K*_a_ and ratios *n*_[bound compound]/[polynucleotide]_ (Table 2). The measured data of fluorescence quenching and an increase in the titration of **23** with poly dA-poly dT and ATT were separately analyzed using the Scatchard equation. In a neutral medium, **23** showed stronger affinity to AT-DNA (poly dA-poly dT, poly(dAdT)_2_) and RNA (poly A-poly U) than to GC-DNA (poly(dGdC)_2_). Additionally, the binding affinities of **23**-DNA/RNA complexes at neutral conditions were higher (log *K*_a_ > 6) than those at lower pH, pH 5.0 (log *K*_a_ < 6). Exceptions were the titrations of **23** with homopolynucelotide poly dA-poly dT and ATT triplex, where analysis of the measured data using the Scatchard equation yielded higher values of binding constants (log *K*_a_ > 6). Compound **22**, an analog of **23**, showed a similar trend in the magnitude of binding affinities, higher log *K*_a_ at neutral conditions, and one order of magnitude lower binding affinities in a weakly acidic medium. In contrast to **23**, **22** did not induce dual fluorimetric response with poly dA-poly dT at pH 5.0. Under neutral conditions, compound **22** induced emission changes significant enough to calculate binding affinity only in titrations with poly(dAdT)_2_ and poly(dGdC)_2_. An accurate calculation of association constants for compound **19** was not possible either because of too small fluorescence changes in titrations with polynucleotides at both pHs or the insufficient number of data points in the non-linear part of the curve (see Figures S6–S37 in SI). 

The binding affinities obtained by titration with the ATT triplex, in which the initial fluorescence of **23** (λ_max_ = 403 nm) was quenched and the fluorescence of the primary complex enhanced by the formation of the secondary complex (λ_max_ = 466 nm), were similar (log *K*_a_ 5.7 and log *K*_a_ 7.4, see Figure S37 in SI) to those obtained in the titration with poly dA-poly dT at pH 5.0. Under neutral conditions, the binding constant of **23** with ATT (log *K*_a_ 7.4, see Figure S21 in SI) was also comparable to that from titration with poly dA-poly dT (Table 2). 

#### 2.3.2. Thermal Melting Experiments

It is widely acknowledged that when ds-helices are heated to a specific temperature (*T*_m_ value), they separate into two single-stranded chains [36]. The non-covalent binding of small molecules to double-stranded polynucleotides typically influences the thermal stability of the helices, leading to variations in *T*_m_ values. The difference between the *T*_m_ values of free polynucleotides and those in complexes with small molecules (Δ*T*_m_ value) is important in characterizing interactions between small molecules and double-stranded polynucleotides. In our investigation, all examined compounds exhibited more pronounced stabilization effects, either on mixed DNA base pairs (with a composition of 58% AT and 42% GC) or on AU and AT base pairs, under weakly acidic pH conditions compared with neutral conditions (Table 3, Figure S38).

**Figure 6:**
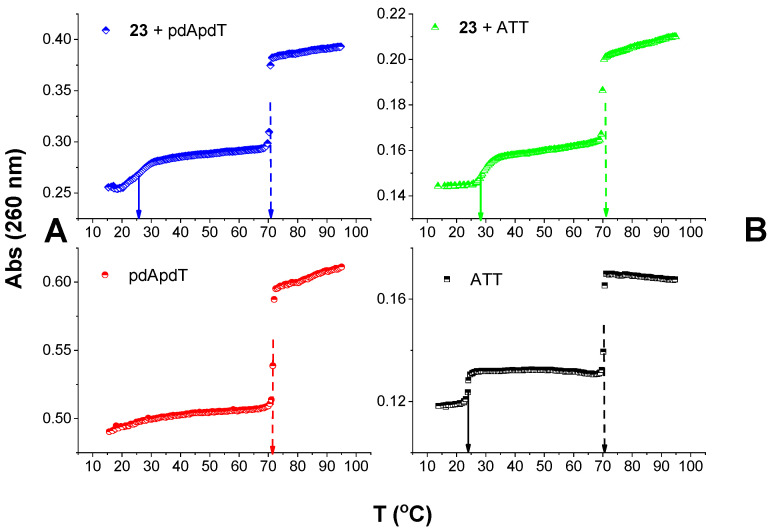
Melting curve of (**A**) pdApdT duplex and (**B**) ATT triplex upon addition of ratio, **r** ([compound]/[nucleotide phosphate]) = 0.3 of **23** at pH 5.0 (sodium cacodylate buffer with NaCl, *I* = 0.1 mol dm^−3^ + 1 mM ethylenediaminetetraacetic acid (EDTA); solid arrows—first transition, dashed arrows—second transition).

Exceptions to that were better stabilization effects of **23** at neutral conditions with RNA and poly dA-poly dT. However, **23** displayed biphasic curves with consecutive AT sequences (Table 3) at experimental buffer conditions (pH 7.0 and pH 5.0, *I* = 0.05 mol dm^−3^). We performed experiments with the ATT triplex at a higher ionic strength, *I* = 0.1 mol dm^−3^ (Figure 6), to check whether this biphasic curve resulted from two different types of binding or induction of triplex formation. We also repeated the thermal melting experiment with poly dA-poly dT with compound **23** at the higher ionic strength, *I* = 0.1 mol dm^−3^, to compare the results under the same ionic conditions (Table 4). 

The first thermal transition, *T*_m3→1_, denotes the separation of the third strand involving poly dT, which forms Hoogsteen base pairs in the major groove (Table 4, Figure 6). The subsequent transition, *T*_m2→1_, signifies the dissociation of the Watson–Crick base pairs of the double-stranded poly dA-poly dT [38,39]. The *T*_m_ values acquired for complexes of **23** with the duplex, under pH 5.0 and ionic strength, *I* = 0.1 mol dm^−3^, are consistent with the *T*_m_ values observed for the ATT triplex [40]. The promotion of triplex formation was not observed with compound **23** at pH 7.0 and higher ionic strength (*I* = 0.1 mol dm^−3^), although this effect was noticeable at a lower ionic strength of *I* = 0.05 mol dm^−3^. 

These findings indicate that compound **23** induces the formation of a triplex structure during its interaction with non-alternating double-stranded configurations. Specifically, compound **23** preferentially stabilized the triplex form over the duplex (*T*_m3→1_ > *T*_m2→1_). Various small ligands, including berenil, berberine, coralyne, palmatine, neomycin, benzothiazoles, and cyanines, demonstrated analogous induction effects on non-alternating nucleic acid sequences containing adenine (dA) [41,42]. Understanding the recognition of AT base pairs is important for drug development that affects DNA replication and repair, which can result in genetic diseases or alterations in protein synthesis [43,44,45].

#### 2.3.3. Circular Dichroism (CD) Experiments

Fluorescence titrations were employed to investigate non-covalent interactions by observing the spectroscopic changes in the compound upon polynucleotide addition. CD spectroscopy is a sensitive technique for detecting conformational changes in the secondary structure of polynucleotides following the binding of small molecules [46].

**Figure 7:**
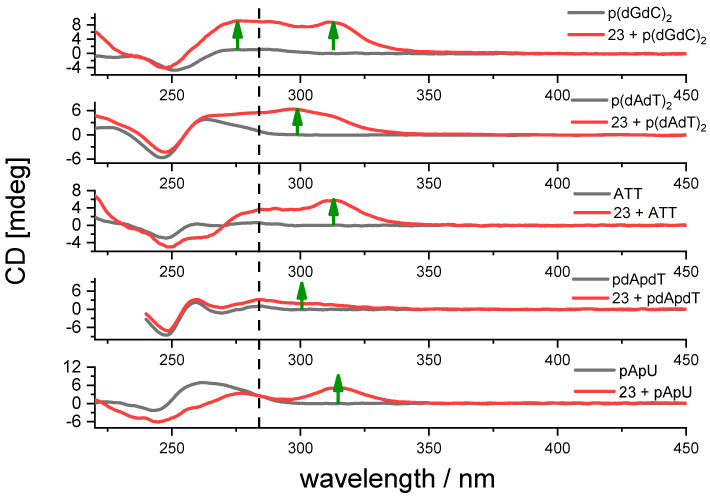
CD titration of p(dGdC)_2_, p(dAdT)_2_, ATT triplex, poly dA-poly dT, and poly A-poly U (*c* = 3.0 × 10^−5^ mol dm^−3^) with **23** at molar ratio **r** = [compound]/[polynucleotide] = 0.5 (pH 5.0, buffer sodium cacodylate, *I* = 0.05 mol dm^−3^).

As the compounds under investigation exhibit chirality and inherently possess intrinsic circular dichroism (CD) activity, we acquired CD spectra of free **19**, **22**, and **23** in an aqueous solution (Figure 3) to clarify more easily the compounds’ influences on the conformation of polynucleotides. CD spectra interpretation in the wavelength region below 300 nm for polynucleotides is challenging because ligands absorb light in that range. However, as **19**, **22**, and **23** also absorb beyond 300 nm, any CD changes in this range refer to their chromophores’ interactions with DNA and RNA (Figure 7 and Figure S40 in SI).

The addition of **19**, **22**, and **23** caused significant changes in the CD spectra of DNA and RNA polynucleotides (260–280 nm) in neutral and weakly acid media. However, the enhanced circular dichroism (CD) signals observed in the CD spectra of DNA and RNA (260–280 nm) and the negative induced circular dichroism (ICD) signals at 309–313 nm during titrations with **19** were comparable in both shape and intensity to the intrinsic CD signals of **19**. However, in the case of poly(dGdC)_2_ at both pH values and poly(dAdT)_2_ at pH 5.0, signals of reduced intensity compared with the intrinsic CD signals of **19** (Figure 3) were observed.

In contrast to **19**, in titrations of **22** and DNA and RNA polynucleotides in neutral and weakly acidic media, negative ICD signals at 309–311 nm were smaller than the intrinsic CD signals of **22** (see Figure 3 and Figure S40 in SI). The addition of **23** to the polynucleotide solution in neutral conditions caused an appearance of positive ICD signals at 311 nm that were lower in intensity than the intrinsic CD bands of **23**. Such a resemblance of CD signals of formed complexes and intrinsic CD bands of studied compounds and spectroscopic (UV/Vis and MS spectra) suggest that **19**, **22**, and **23** bind to polynucleotides in folded conformation either in grooves or in their vicinity along the polynucleotide backbone. 

Nevertheless, in titration with the ATT triplex at pH 5.0 and all studied polynucleotides at pH 5.0, the ICD signals were significantly more positive than the intrinsic CD signals of **23**. This increase in ICD intensity at 300 or 313 nm was most apparent in titrations with the ATT triplex, poly(dAdT)_2_, and poly(dGdC)_2_ (Figure 7). The stabilizing impact observed with poly(dAdT)_2_ and positive ICD signals detected in CD titrations with ctDNA, poly(dAdT)_2_, and poly(dGdC)_2_ (particularly notable at pH 5.0) suggests the binding of **23** inside the minor groove [47,48]. The positive ICD signal (around 314 nm) also appeared upon the binding of **23** to poly A-poly U. Considering the dimensions of the minor groove (broad and very shallow) of RNA (poly A-poly U), the major groove is probably the dominant binding site [49,50]. Noteworthily, **23** induced a significant increase in the intensity of polynucleotide CD maximum at 275 nm only with poly(dGdC)_2_ and GC-containing polynucleotide (ctDNA) (Figure S40 in SI).

In addition, the CD bands induced by RNA at 285 nm are close to zero (see vertical line in Figure 7). In contrast, there was a substantial increase in intensity in the DNA-induced CD bands. Therefore, **23** can serve as a straightforward chiral probe for GC-rich DNA and differentiation between ds-RNA (A-helical structure) and ds-DNA (B-helix). GC recognition is biologically relevant since numerous protein-coding genes contain GC-rich isochores [51]. Furthermore, specific segments of the human genome, such as the regulatory regions of oncogenes and the genome of the Epstein–Barr virus, exhibit exceptionally high guanine–cytosine contents, surpassing 80% [52].

### 2.4. Computational Analysis of Systems ***19***, ***22***, and ***23*** in Aqueous Solution

The focus of the computational analysis was on inspecting whether the intrinsic dynamics and conformational preference of the investigated systems determine trends in their ability to recognize nucleic acid sequences reported in this work. This agrees with our earlier efforts on similar derivatives that demonstrated an inverse correlation between significant intramolecular contacts and affinities towards polynucleotides [18,53,54]. In this way, we successfully interpreted the biological sensing properties as a compromise between each derivative’s tendency to form stable intramolecularly connected structures and those with a liberated phenanthridine, deprived of any notable intramolecular contacts, which allows it to interact with nucleic acid components more freely. With this in mind, we carried out several molecular dynamics simulations of different protonation forms of **19**, **22**, and **23**, corresponding to the employed pH conditions, and analyzed their conformational preferences in the obtained trajectories.

**Figure 8:**
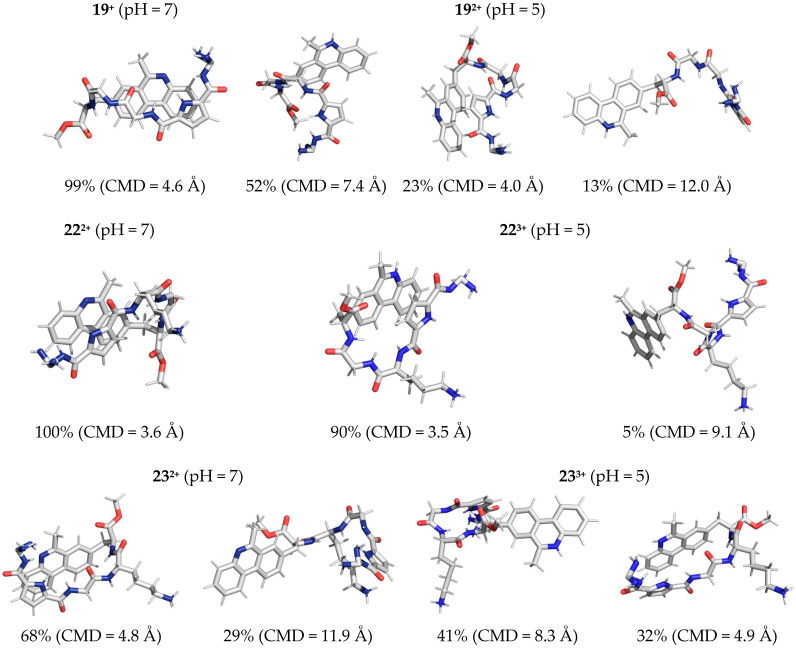
Most representative structures of **19**, **22**, and **23** under diverse pH conditions, together with their total populations during MD simulations (in %) and the center of mass distance (CMD) between phenanthridine and pyrrole rings (in Å). These are elucidated after the clustering analysis of the matching MD trajectories.

A combination of clustering (Figure 8) and RDF analyses (Figure S42) indicates that all three conjugates displayed notable tendencies to form intramolecularly connected structures under both pH conditions, which likely prevented them from efficiently recognizing polynucleotides in solution. These are established through several stabilizing π–π stacking contacts, mostly including pyrrole and phenanthridine rings, structurally connected by flexible linkers that allow them to approach each other. The latter is evident in RDF displays describing the evolution of center of mass distances (CMDs) between these ring elements, particularly as peaks at values lower than 5 Å, indicating strong and persistent stacking interactions. 

System **19**, without lysine as the linking residue, is the least charged among all three derivatives. As such, it is rational to anticipate that the neutral Gly-Gly linker would introduce plenty of flexibility into the molecular structure, while not interfering with a likely favorable π–π stacking interaction between phenanthridine and pyrrole bearing a charged guanidine. This is certainly the case, as at pH = 7, we identified 99% of structures resembling stacked conformation with a rather low CMD = 4.6 Å. This agrees with the matching RDF display showing a strong peak at the same value and two lower peaks below 5 Å. It is precisely this exclusivity of stacked conformations to which we ascribe the demonstrated absence of the polynucleotide sensing ability of **19** under neutral conditions. The situation is not significantly improved upon acidifying the solution to pH = 5, although the matching RDF graph revealed some broadening at values exceeding 6 Å. There, bent conformations are grouped into two clusters as follows: the first represents those with RDFs below 5 Å (23%) and the second represents those with RDFs between 5 and 10 Å (52%). In total, this implies that 75% of the solution structures exhibit intramolecularly stacked conformations. In contrast, RDF signals above 10 Å originate from 13% of non-stacked structures, where phenanthridine is devoid of intramolecular stacking contacts. Still, such a low percentage of structures with an improved ability to interact with polynucleotides is insignificant since no interactions with polynucleotides were recorded experimentally. 

It is reasonable to expect that the introduction of the positively charged lysine within the linker unit would disrupt the described π–π stacking interactions through charge repulsions, thus promoting polynucleotide sensing. Indeed, both **22** and **23**, under either pH conditions, exhibited reduced populations of stacked structures followed by assessable binding constants (Table 2). Still, by enabling that, the position of the introduced lysine and the phenanthridine protonation state both proved to be important factors for the magnitude of the resulting effect. Interestingly, for dicationic **22^2+^** at pH = 7, all structures recorded during MD simulations still clustered into a single conformation with prominent π–π stacking contacts. The latter is evident in the lowest phenanthridine–pyrrole CMD of only 3.6 Å in the representative structure (Figure 8), consistent with a very sharp and high peak at 3.5 Å in the matching RDF graph (Figure S42). This explains its low activity among lysine-containing derivatives, associated with a complete lack of binding affinity for ctDNA, pApU, and pdApdT. In contrast, lysine becomes more efficient in dicationic **23^2+^** at pH = 7. Only 68% of structures retained stacked conformations, while 29% offered a more liberated phenanthridine. The latter resulted in a much more consistent biological activity of **23^2+^** towards all investigated polynucleotides over that in **22^2+^**.

When pH conditions were lowered, the overall sensing ability of **22^3+^** became much broader, as it showed moderate affinities for all studied polynucleotides except pdApdT, being consistent with a 5% improvement in the number of relevant conformers without stacking interactions. As was the case among derivatives at pH = 7, under acidic conditions, the effect of lysine again turned out as more optimal in **23^3+^**, which revealed further advances in its sensing ability over **22^3+^** through 41% of its non-stacked structures. The latter culminated with the highest affinity reported here of log *K*_a_ = 7.4 measured towards pdApdT, which highlights the utility of system **23** as a useful chiral probe and confirms the experimental observations. These results underline a very significant effect of the lysine residue and its position on the sensing properties. We note in passing that this influence is not linked with its immediate contacts with the phenanthridine ring through potential cation–π interactions. In contrast, during MD simulations, the average distances between the charged lysine nitrogen and the phenanthridine center of mass are 7.3 Å (in **22^2+^**) and 8.7 Å (in **23^2+^**) at pH = 7, which reveal no direct interactions. As expected, these were further increased with protonated phenanthridine at pH = 5, where they assumed 12.3 Å (in **22^3+^**) and 12.8 Å (in **23^3+^**). This is a result of the fact that the lysine sidechain comes with a very localized positive charge on its amine, which prefers its solvation over undergoing hydrophobic cation–π interactions. This leads to the conclusion that lysine exerted its favorable effect by hindering the flexibility and modulating the electrostatic environment around each derivative, which jointly interfered with the π–π interactions among terminal ring fragments. 

**Figure 9:**
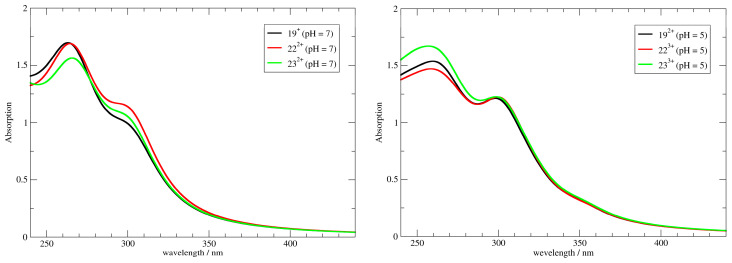
Calculated UV/Vis spectral lines for the representative structures of **19**, **22**, and **23** under normal (pH = 7, left) and acidic conditions (pH = 5, right) obtained with the TD-DFT approach and the (SMD)/B3LYP/6–31+G(d) model in water.

In the end, since our computational analysis relied on identifying representative conformations of each derivative under two pH conditions, we decided to verify the accuracy of this selection and check whether the studied systems are indeed well represented with the most dominant cluster structures in Figure 8. For that purpose, we calculated the energies of the excited states shaping the experimental UV/Vis spectra by taking the most abundant structures of each isolated conjugate, in line with our earlier reports [18,53,54]. The obtained vertical transitions (Figure 9) agree well with experiments (Figure 2, Table 1), indicating absorption maxima at 264 and 296 nm for all three systems at pH = 7, while 258 and 300 nm at pH = 5. We believe this lends credence to the utilized computational strategy and confirms the clustering analysis validity. 

Lastly, we investigated the efficiency of UV/Vis spectroscopy to differentiate biologically less active bent conformations from those with not as much hindered phenanthridine unit that appear more potent in this respect. For this, we utilized data for system **23** since it allowed notable percentages of both types of conformations under either pH condition (Figure 8). It turned out that computed spectra for both of its conformers at pH = 7 are highly overlapped, especially in the position of absorption maxima, and so are those at pH = 5 (Figure S43). The underlying conformational changes, although substantial, do not appear to induce major changes in the photophysical UV/Vis responses. Therefore, this technique seems moderately sensitive to distinguish between bent and open conformations under employed conditions. To further substantiate this conclusion, we calculated total atomic charges within phenanthridine in each case and evaluated how they are modified depending on its stacking contacts with pyrrole. Remarkably, in bent conformations, the charge within the entire phenanthridine ring is −0.02 |e| in **23^2+^** and 0.98 |e| in **23^3+^**, while it assumes −0.02 |e| in **23^2+^** and 0.96 |e| in **23^3+^** when these are in open conformations, which strongly confirm minor changes in both computed and experimentally recorded UV/Vis spectra. Overall, our analysis underlined that the sensing abilities of the studied conjugates critically depend on their conformational preferences, which are manifested as pure steric effects, while they do not occur because of, for example, any charge transfer or polarization effects between stacked fragments.

### 2.5. Biological Activity of ***19***, ***22***, and ***23***

**Figure 10:**
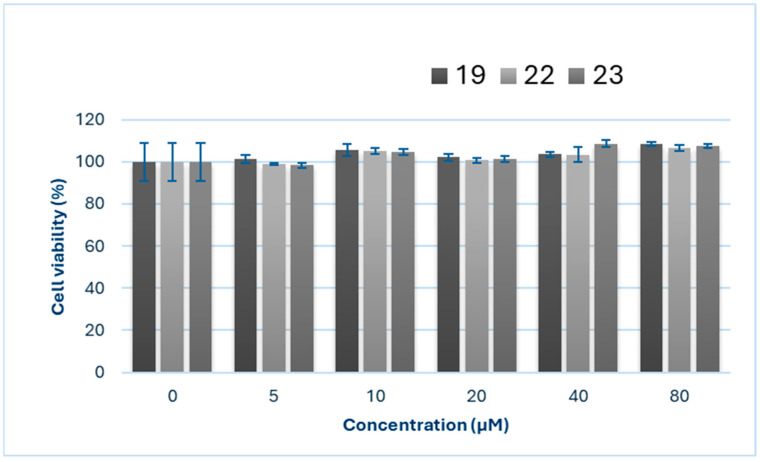
The impact of compounds **19**, **22**, and **23** on the viability of HeLa cells. The HeLa cells were treated with the indicated concentrations of compounds **19**, **22**, and **23**. After 72 h of incubation, the effect on cell viability was measured by the MTT assay.

Since the impact of compounds **19**, **22**, and **23** on cell viability has not been examined so far, we decided to test their biological activity using the cervical tumor HeLa cell line (Figure 10). All tested compounds did not impact cell viability in the concentration range used. To avoid solvent toxicity (DMSO), concentrations above 80 μM were not used.

## 3. Materials and Methods

### 3.1. Synthetic Procedures

#### 3.1.1. Synthesis of HOOC-Gly-Lys-GCP-Boc (**1a**)

1. **Cbz-protection** of **2** to obtain acid **3**

**Figure 11:**
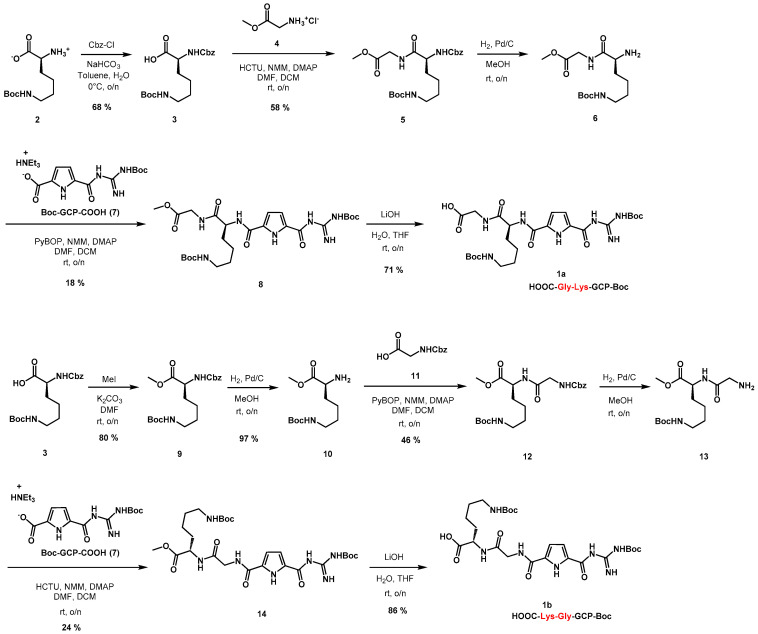
Synthesis of **1a** and **1b**.

Compound **2** (3.0 g, 12.2 mmol, 1.0 eq.) was suspended in water (50 mL) (Figure 11). The suspension was cooled in an ice bath, and sodium bicarbonate (2.6 g, 30.9 mmol, 2.5 eq.) was added until **2** was completely dissolved. Then, Cbz-Cl (2.5 g, 14.6 mmol, 1.2 eq., 98%) dissolved in toluene (5 mL) was added. The reaction mixture was stirred at 0 °C overnight. The solvent was removed under vacuum. The dry crude was suspended in distilled water (200 mL) and purified by extraction with diethyl ether (100 mL) once. After the separation of the organic and aqueous phases, a concentrated solution of citric acid was added to the aqueous phase until the formation of a precipitate was noticed. The precipitate was then extracted with ethyl acetate (2 × 200 mL). The organic phase was dried over magnesium sulfate. The solvent was evaporated, and the residue was dried under reduced pressure, yielding **3** as a colorless oil (3.16 g, 8.3 mmol, 68%). 

**^1^H NMR** (300 MHz, [D_6_]DMSO, 25 °C): δ/ppm = 1.26–1.41 (m, 9H + 4H, Boc-CH_3_ + CH_2_), 1.45–1.74 (m, 2H, CH_2_), 2.80–2.95 (m, 2H, CH_2_), 3.83–3.97 (m, 1H, CH), 5.03 (s, 2H, Cbz-CH_2_), 6.64–6.82 (m, 1H, Boc-NH), 7.12–7.41 (m, 5H, aryl-CH), 7.42–8.08 (d, 1H, Cbz-NH), 12.46 (br.s, 1H, COOH); **^13^C NMR** (100 MHz, [D_6_]DMSO, 25 °C): δ/ppm = 23.0 (CH_2_), 28.3 (Boc-CH_3_), 29.1 (CH_2_), 30.5 (CH_2_), 39.6 (CH_2_), 53.9 (CH), 65.4 (Cbz-CH_2_), 77.4 (Boc-Cq), 127.7 (aryl-CH), 127.9 (aryl-CH), 128.4 (aryl-CH), 137.1 (aryl-Cq), 155.6 (Cq), 156.3 (Cq), 174.0 (Cq); **HRMS** (ESI): *m*/*z* = calc. for C_19_H_28_N_2_O_6_: 379.1875 [M-H]^−^; found 379.1954 [M-H]^−^.

2. **Coupling** of **3** with **4** to obtain peptide **5**

A solution of **3** (1.5 g, 3.9 mmol, 1.0 eq.), HCTU (2.0 g, 4.8 mmol, 1.2 eq., ≥98%), NMM (3 mL, 27.2 mmol, 7 eq., 99%), and DMAP (catalytic amount, ≥99%) in a mixture of DMF and DCM (1:2, 100 mL) was stirred at room temperature for 30 min. A solution of compound **4** (0.5 g, 4.0 mmol, 1.0 eq.) in a mixture of DMF and DCM (1:2, 60 mL) was added slowly, followed by NMM (0.5 mL). The reaction mixture was stirred at room temperature overnight. The solvents were evaporated, and the residue was dissolved in 50 mL of methanol. The methanol solution of the crude was then mixed with 1 L of water and stirred until the formation of a precipitate. The precipitate was extracted with ethyl acetate (3 × 200 mL), and the obtained organic phase was dried over magnesium sulfate. The solvent was removed under reduced pressure, and the residue was purified by column chromatography (ethyl acetate/cyclohexane = 5/5) yielding **5** as a colorless solid (1.03 g, 2.3 mmol, 58%). 

**^1^H NMR** (300 MHz, [D_6_]DMSO, 25 °C): δ/ppm = 1.24–1.42 (m, 9H + 4H, Boc-CH_3_ + CH_2_), 1.43–1.70 (m, 2H, CH_2_), 2.79–2.97 (m, 2H, CH_2_), 3.62 (s, 3H, CH_3_), 3.7–3.94 (m, 2H, CH_2_), 3.94–4.05 (m, 1H, CH), 5.03 (s, 2H, Cbz-CH_2_), 6.74 (1, 1H, Boc-NH), 7.1–7.52 (m, 5H + 1H, aryl-CH + Lys-NH), 8.31 (t, 1H, Gly-NH); **^13^C NMR** (75 MHz, [D_6_]DMSO, 25 °C): δ/ppm = 22.6 (CH_2_), 28.2 (Boc-CH_3_), 29.1 (CH_2_), 31.5 (CH_2_), 39.6 (CH_2_), 40.4 (CH_2_), 51.5 (CH_3_), 54.4 (CH), 65.3 (Cbz-CH_2_), 77.3 (Boc-Cq), 127.6 (aryl-CH), 127.7 (aryl-CH), 128.3 (aryl-CH), 127.6 (aryl-Cq), 155.5 (Cq), 155.9 (Cq), 170.2 (Cq), 172.5 (Cq).

3. **Cbz-deprotection** of **5** to obtain amine **6**; **coupling** of **6** with **7** to obtain **8**

Compound **5** (1.0 g, 2.2 mmol) and Pd/C (100 mg, 10%) were suspended in methanol (100 mL) under a hydrogen atmosphere and stirred at room temperature. The reaction was monitored by thin-layer chromatography (TLC). After TLC showed a complete consumption of the starting material, the reaction mixture was filtered through celite. The solvent was evaporated, which yielded compound **6** as a colorless oil. The compound was then submitted to the next step of synthesis without further purification. A solution of **7** [24] (0.75 g, 1.9 mmol, 1.0 eq.), PyBOP (1.1 g, 2.1 mmol, 1.1 eq., ≥97.0%), NMM (1.3 mL, 11.8 mmol, 6 eq., 99%), and DMAP (catalytic amount, ≥99%) was stirred in a mixture of DMF and DCM (1:2, 150 mL) at room temperature for 30 min. A solution of amine **6** (0.60 g, 1.9 mmol, 1.0 eq.) in DCM (50 mL) was added, followed by NMM (0.5 mL). The reaction mixture was stirred overnight at room temperature. The solvents were removed under vacuum, and the crude was dissolved in 50 mL methanol. The methanol solution of the crude was then mixed with 1 L of water and stirred until the formation of a precipitate. The precipitate was extracted with ethyl acetate (3x200 mL), and the organic phase was dried over magnesium sulfate. The solvent was removed under reduced pressure. The crude was purified by column chromatography (mplc/RP18; methanol/water) yielding **8** as a colorless solid (0.24 g, 0.40 mmol, 18%). 

**^1^H NMR** (300 MHz, [D_6_]DMSO, 25 °C): δ/ppm = 1.25–1.48 (m, 9H + 9H + 4H, Boc-CH_3_ + CH_2_), 1.53–1.81 (m, 2H, CH_2_), 2.79–2.96 (m, 2H, CH_2_), 3.61 (s, 3H, CH_3_), 3.74–3.95 (m, 2H, CH_2_), 4.33–4.52 (m, 1H, CH), 6.54–7.01 (m, 2H + 1H, pyrrole-CH + Boc-NH), 8.19–8.50 (m, 1H + 1H, Gly-NH + Lys-NH), 8.55 (br.s, 1H, guanidino-NH), 9.32 (br.s, 1H, guanidino-NH), 10.86 (br.s, 1H, guanidino-NH), 11.56 (br.s, 1H, pyrrole-NH); **^13^C NMR** (75 MHz, [D_6_]DMSO, 25 °C): δ/ppm = 22.8 (CH_2_), 27.7 (Boc-CH_3_), 28.2 (Boc-CH_3_), 29.2 (CH_2_), 31.6 (CH_2_), 39.6 (CH_2_), 40.5 (CH_2_), 51.6 (CH_3_), 52.5 (CH), 77.3 (Boc-Cq), 112.9 (pyrrole-CH), 113.5 (pyrrole-CH), 152.9 (Cq), 155.5 (Cq), 158.4 (Cq), 159.4 (Cq), 170.2 (Cq), 172.3 (Cq); **HRMS** (ESI): *m*/*z* = calc. for C_26_H_41_N_7_O_9_: 596.3039 [M+H]^+^; found 596.3230 [M+H]^+^.

4. **Methyl ester deprotection** of **8** to obtain acid **1a**

A solution of **8** (0.23 g, 0.39 mmol, 1.0 eq.) and lithium hydroxide (0.10 g, 2.4 mmol, 6 eq.) in a mixture of THF and water (4:1, 150 mL) was stirred overnight at room temperature (Figure 11). After removing THF from the solvent mixture, a concentrated solution of citric acid was added to the remaining water solution, which caused the formation of a precipitate. The precipitate was extracted with ethyl acetate (3 × 100 mL). The organic phase was washed with water and dried over magnesium sulfate. The solvent was removed under reduced pressure, and the residue was dried, yielding HOOC-Gly-Lys-GCP-Boc (**1a**) as a colorless solid (0.16 g, 0.28 mmol, 71%). 

**^1^H NMR** (300 MHz, [D_6_]DMSO, 25 °C): δ/ppm = 1.18–1.78 (m, 9H + 9H + 6H, Boc-CH_3_ + CH_2_), 2.79–2.97 (m, 2H, CH_2_), 3.81–4.03 (m, 2H, CH_2_), 4.11–4.27 (m, 1H, CH), 6.63–6.94 (m, 2H + 1H, pyrrole-CH + Boc-NH), 8.20 (t, 1H, Lys-NH), 8.33–8.89 (m, 1H, Gly-NH), 8.60–9.32 (2br.s, 1H, guanidino-NH), 11.02–12.74 (m, 2H + 1H + 1H, guanidino-NH + pyrrole-NH + COOH); **^13^C NMR** (100 MHz, [D_6_]DMSO, 25 °C): δ/ppm = 22.9 (CH_2_), 27.8 (Boc-CH_3_), 28.3 (Boc-CH_3_), 29.3 (CH_2_), 31.7 (CH_2_), 39.8 (CH_2_), 40.7 (Gly-CH_2_), 52.6 (CH), 77.4 (Boc-Cq), 113.0 (pyrrole-CH), 113.7 (pyrrole-CH), 125.5 (pyrrole-Cq), 130.2 (pyrrole-Cq), 155.6 (Cq), 158.5 (Cq), 159.2 (Cq), 159.5 (Cq), 161.7 (Cq), 171.2 (Cq), 172.1 (Cq), 172.2 (Cq), 172.3 (Cq); **HRMS** (ESI): *m*/*z* = calc. for C_25_H_39_N_7_O_9_: 580.2736 [M-H]^−^; found 580.3790 [M-H]^−^. 

#### 3.1.2. Synthesis of HOOC-Lys-Gly-GCP-Boc (**1b**)

1. **Protection** of **3** to obtain methyl ester **9**

Compound **3** (1.55 g, 4.0 mmol, 1.0 eq.) and potassium carbonate (5.5 g, 40 mmol, 10 eq.) were suspended in dry DMF (40 mL) and stirred at 10 °C. Then, methyl iodide (11.4 g, 80 mmol, 20 eq.) was slowly added, and the stirring of the reaction mixture was continued overnight at room temperature. After completion of the reaction, the solvent was removed under reduced pressure. The crude was dissolved in water and extracted with ethyl acetate (2 × 200 mL). The organic phase was dried over magnesium sulfate, the solvent was removed in vacuo, and the residue was purified by column chromatography (ethyl acetate/cyclohexane = 5/5), yielding **9** as a colorless oil (1.29 g, 3.2 mmol, 80%). 

**^1^H NMR** (300 MHz, [D_6_]DMSO, 25 °C): δ/ppm = 1.19–1.43 (m, 9H + 4H, Boc-CH_3_ + CH_2_), 1.44–1.73 (m, 2H, CH_2_), 2.75–2.95 (m, 2H, CH_2_), 3.60 (s, 3H, CH_3_), 3.88–4.08 (m, 1H, CH), 5.02 (s, 2H, Cbz-CH_2_), 6.52–6.91 (d, 1H, Boc-NH), 7.13–7.44 (m, 5H, aryl-CH), 7.48–7.79 (d, 1H, Cbz-NH); **^13^C NMR** (75 MHz, [D_6_]DMSO, 25 °C): δ/ppm = 22.7 (CH_2_), 28.2 (Boc-CH_3_), 28.9 (CH_2_), 30.3 (CH_2_), 39.4 (CH_2_), 51.7 (CH_3_), 53.8 (CH), 65.5 (Cbz-CH_2_), 77.3 (Boc-Cq), 127.7 (aryl-CH), 127.8 (aryl-CH), 128.3 (aryl-CH), 136.9 (aryl-Cq), 155.5 (Cq), 156.1 (Cq), 172.9 (Cq).

2. **Cbz-deprotection** of **9** to obtain amine **10**

Compound **9** (1.25 g, 3.1 mmol) was suspended in methanol (100 mL) under a hydrogen atmosphere and Pd/C (150 mg, 10%) was added. The reaction mixture was stirred at room temperature until TLC revealed complete consumption of the starting material. The reaction mixture was then filtered through celite to remove the catalyst. The solvent was removed under reduced pressure to yield **10** as a colorless oil (0.80 g, 3.0 mmol, 97%). 

**^1^H NMR** (300 MHz, [D_6_]DMSO, 25 °C): δ/ppm = 1.15–1.60 (m, 9H + 6H, Boc-CH_3_ + CH_2_), 1.62–2.15 (br.s, 2H, NH_2_), 2.78–2.95 (m, 2H, CH_2_), 3.19–3.32 (m, 1H, CH), 3.60 (s, 3H, CH_3_), 6.55–6.82 (m, 1H, Boc-NH); **^13^C NMR** (75 MHz, [D_6_]DMSO, 25 °C): δ/ppm = 22.4 (CH_2_), 28.1 (Boc-CH_3_), 29.2 (CH_2_), 34.2 (CH_2_), 39.6 (CH_2_), 51.3 (CH_3_), 53.9 (CH), 77.1 (Boc-Cq), 155.5 (Cq), 176.2 (Cq).

3. **Coupling** of **10** with **11** to obtain peptide **12**

Compound **11** (0.70 g, 3.3 mmol, 1.1 eq.) was dissolved in a mixture of DMF and DCM (1:2, 100 mL) and PyBOP (2.0 g, 3.8 mmol, 1.2 eq., ≥97.0%), and then NMM (2.0 mL, 18.2 mmol, 6 eq., 99%) and DMAP (catalytic amount, ≥99%) were added. The reaction was stirred for 30 min at room temperature. Then, a solution of compound **10** (0.80 g, 3.0 mmol, 1.0 eq.) in DCM (50 mL) was added, followed by NMM (0.5 mL). The reaction mixture was stirred overnight at room temperature. The solvents were removed under vacuum, and the residue was dissolved in 50 mL methanol. The obtained methanol solution was mixed with 1 L of water and stirred until a precipitate was noticed. The precipitate was then extracted with ethyl acetate (3x100 mL). The organic phase was dried over magnesium sulfate. The solvent was removed under reduced pressure, and the residue was purified by column chromatography (mplc/RP18; methanol/water) yielding **12** as a colorless solid (0.63 g, 1.4 mmol, 46%).

**^1^H NMR** (300 MHz, [D_6_]DMSO, 25 °C): δ/ppm = 1.15–1.45 (m, 9H + 4H, Boc-CH_3_ + CH_2_), 2.75–2.96 (m, 2H, CH_2_), 3.49–3.73 (m, 3H + 2H, CH_3_ + CH_2_), 4.13–4.31 (m, 1H, CH), 5.02 (s, 2H, Cbz-CH_2_), 6.75 (t, 1H, Boc-NH), 7.13–7.55 (m, 5H + 1H, aryl-CH + Gly-NH), 8.20 (d, 1H, Lys-NH); **HRMS** (ESI): *m*/*z* = calc. for C_22_H_33_N_3_O_7_: 474.2211 [M+Na]^+^; found 474.2241 [M+Na]^+^.

4. **Cbz-deprotection** of **12** to obtain amine **13**; **coupling** of **13** with **7** to obtain **14**

To a suspension of **12** (0.62 g, 1.37 mmol) in methanol (100 mL), Pd/C (100 mg, 10%) (100 mL) was added. The reaction mixture was stirred at room temperature, under a hydrogen atmosphere until TLC indicated a complete consumption of the starting compound. Then, the reaction mixture was filtered through celite and washed with methanol (2 × 50 mL). The solvent was removed in vacuo to yield **13** as a colorless oil. The compound was then submitted to the next step of synthesis without further purification. A solution of **7** [24] (0.55 g, 1.38 mmol, 1.1 eq.), HCTU (0.7 g, 1.7 mmol, 1.4 eq., ≥98%), NMM (1.0 mL, 9.1 mmol, 7 eq., 99%), and DMAP (catalytic amount, ≥99%) was stirred for 30 min in a mixture of DMF and DCM (1:2, 100 mL) at room temperature. A solution of amine **13** (0.39 g, 1.22 mmol, 1.0 eq.) in DCM (50 mL) was added, followed by NMM (0.2 mL), and stirred overnight at room temperature. After the solvent was evaporated, the residue was dissolved in methanol (50 mL) and subsequently mixed with water (500 mL). The mixture was stirred until the formation of a precipitate. The precipitate was extracted with ethyl acetate (2 × 200 mL), and the organic phase was dried over magnesium sulfate. The solvent was removed in vacuo, and the crude was purified by column chromatography (mplc/RP18; methanol/water), yielding **14** as a colorless solid (0.198 g, 0.33 mmol, 24%).

**^1^H NMR** (300 MHz, [D_6_]DMSO, 25 °C): δ/ppm = 1.20–1.51 (m, 9H + 9H + 4H, Boc-CH_3_ + CH_2_), 1.51–1.78 (m, 2H, CH_2_), 2.78–2.97 (m, 2H, CH_2_), 3.62 (s, 3H, CH_3_), 3.77–4.05 (m, 2H, CH_2_), 4.16–4.32 (m, 1H, CH), 6.53–7.07 (m, 2H + 1H, pyrrole-CH + Boc-NH), 8.34 (d, 1H, Lys-NH), 8.57 (br.s, 1H, guanidino-NH), 8.62 (t, 1H, Gly-NH), 9.31 (br.s, 1H, guanidino-NH), 10.85 (br.s, 1H, guanidino-NH), 11.34 (br.s, 1H, pyrrole-NH); **^13^C NMR** (75 MHz, [D_6_]DMSO, 25 °C): δ/ppm = 22.5 (CH_2_), 27.7 (Boc-CH_3_), 28.1 (Boc-CH_3_), 28.9 (CH_2_), 30.5 (CH_2_), 39.5 (CH_2_), 41.4 (CH_2_), 51.7 (CH_3_), 51.9 (CH), 77.3 (Boc-Cq), 112.1 (pyrrole-CH), 113.4 (pyrrole-CH), 151.7 (Cq), 155.5 (Cq), 158.4 (Cq), 159.8 (Cq), 169.0 (Cq), 172.5 (Cq); **HRMS** (ESI): *m*/*z* = calc. for C_26_H_41_N_7_O_9_: 618.2858 [M+Na]^+^; found 618.3327 [M+Na]^+^.

5. **Methyl ester deprotection** of **14** to obtain acid **1b**

Compound **14** (0.19 g, 0.32 mmol, 1.0 eq.) and lithium hydroxide (0.10 g, 2.4 mmol, 7 eq.) were dissolved in a mixture of THF and water (4:1, 150 mL) and stirred overnight at room temperature (Figure 11). After removing THF from the solvent mixture, a concentrated solution of citric acid solution was added to the aqueous solution until a colorless precipitate was noticed. The formed precipitate was extracted with ethyl acetate (3x100 mL). The organic phase was washed with water thoroughly and dried over magnesium sulfate. The solvent was then removed in vacuo to yield HOOC-Lys-Gly-GCP-Boc (**1b**) as a colorless solid (0.16 g, 0.28 mmol, 86%). 

**^1^H NMR** (300 MHz, [D_6_]DMSO, 25 °C): δ/ppm = 1.11–1.76 (m, 9H + 9H + 6H, Boc-CH_3_ + CH_2_), 2.74–2.97 (m, 2H, CH_2_), 3.77–4.01 (m, 2H, CH_2_), 4.1–4.26 (m, 1H, CH), 6.58–6.96 (m, 2H + 1H, pyrrole-CH + Boc-NH), 8.19 (t, 1H, Lys-NH), 8.36.8.83 (m, 1H, Gly-NH), 8.59–9.32 (2br.s, 1H, guanidino-NH), 11.0–12.91 (m, 2H + 1H + 1H, guanidino-NH + pyrrole-NH + COOH); **^13^C NMR** (100 MHz, [D_6_]DMSO, 25 °C): δ/ppm = 22.7 (CH_2_), 27.8 (Boc-CH_3_), 28.3 (Boc-CH_3_), 29.1 (CH_2_), 30.9 (CH_2_), 39.7 (CH_2_), 41.7 (Gly-CH_2_), 51.9 (CH), 77.4 (Boc-Cq), 112.2 (pyrrole-CH), 113.8 (pyrrole-CH), 125.6 (pyrrole-Cq), 130.2 (pyrrole-Cq), 155.6 (Cq), 158.4 (Cq), 159.6 (Cq), 159.9 (Cq), 161.6 (Cq), 168.8 (Cq), 168.9 (Cq), 172.1 (Cq), 173.6 (Cq); **HRMS** (ESI): *m*/*z* = calc. for C_25_H_39_N_7_O_9_: 580.2736 [M-H]^−^; found 580.3529 [M-H]^−^. 

#### 3.1.3. General Procedure for Peptide Coupling—Synthesis of **16**, **17**, **18**, **20**, and **21**

**Figure 12:**
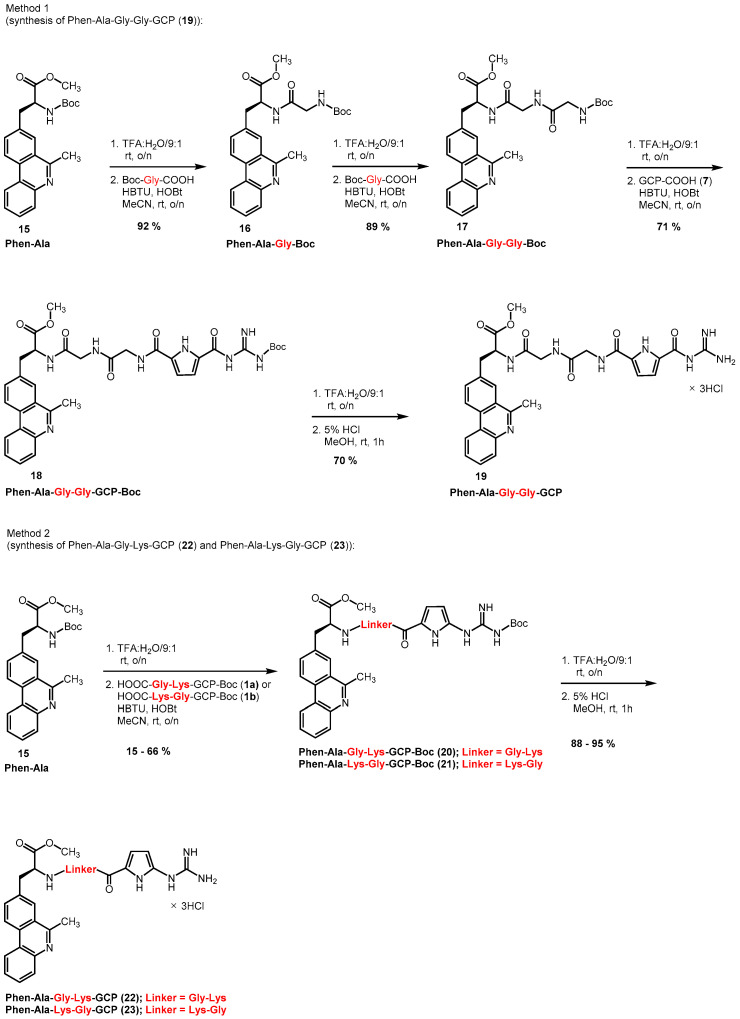
Synthesis of compounds **19**, **22**, and **23**.

Peptide derivatives **16**, **17**, **18**, **20**, and **21** were prepared by a known method of peptide coupling [18], with slight modification (Figure 12). Briefly, to a solution of the corresponding Boc-protected amino acid derivative in DCM (2 mL) was added a mixture of TFA and water (TFA:H_2_O/9:1, 1 mL). The reaction mixture was stirred overnight at room temperature. The removal of the solvent under reduced pressure afforded a triflouroacetate salt of the corresponding amine as a yellow oil. The obtained compound was dissolved in dry MeCN under argon, and an equimolar amount of the corresponding carboxylic acid, HBTU, and HOBt was added to the solution, followed by Et_3_N (4–8 molar equivalents). The reaction mixture was stirred overnight at room temperature. The desired product was isolated by preparative thin-layer chromatography (DCM:MeOH/9:1).

1. Synthesis of Phen-Ala-Gly-Boc (**16**):

Phen-Ala ((**15)**, 11.8 mg, 0.03 mmol), Boc-Gly-COOH (5.3 mg, 0.03 mmol), HBTU (11.6 mg, 0.03 mmol, 98%), HOBt (4.2 mg, 0.03 mmol, 97%), Et_3_N (16.8 µL, 0.12 mmol), and MeCN (1 mL) were used according to the general procedure for peptide coupling. Preparative thin layer chromatography afforded Phen-Ala-Gly-Boc (**16**) as a light-yellow solid (12.4 mg, 92%).

M.p. = 63–65 °C; R*_f_* = 0.6 (CH_2_Cl_2_:MeOH/9:1); ^1^H NMR (CDCl_3_) δ/ppm: 8.59–8.42 (m, 2H, Phen), 8.07 (d, *J* = 8.0 Hz, 1H, Phen), 7.93 (s, 1H, Phen), 7.75–7.54 (m, 3H, Phen), 6.83 (bd, *J* = 6,7 Hz, 1H, NH), 5.19 (bs, 1H, NH), 5.08–4.95 (m, 1H, CH), 3.92–3.68 (m, 5H, OCH_3_, CH_2_), 3.48–3.28 (m, 2H, CH_2_), 3.01 (s, 3H, CH_3_), 1.36 (s, 9H, CH_3_-Boc); ^13^C NMR (CDCl_3_) δ/ppm: 171.60 (C_q_), 169.37 (C_q_), 158.51 (C_q_), 143.59 (C_q_), 135.07 (C_q_), 131.70 (CH), 131.58 (C_q_), 129.30 (CH), 128.61 (CH), 126.88 (CH), 126.40 (CH), 125.97 (C_q_), 123.54 (C_q_), 122.74 (CH), 121.87 (CH), 80.39 (C(CH_3_)_3_-Boc), 53.22 (CH or OCH_3_), 52.50 (CH orOCH_3_), 44.33 (CH_2_), 38.12 (t, CH_2_), 28.15 (CH_3_-Boc), 23.36 (CH_3_); M_w_ = 451,51; ESI-MS: *m*/*z* 452.4 [M+H]^+^.

2. Synthesis of Phen-Ala-Gly-Gly-Boc (**17**):

Phen-Ala-Gly-Boc ((**16**), 22.9 mg, 0.05 mmol), Boc-Gly-COOH (10.5 mg, 0.06 mmol), HBTU (19.3 mg, 0.05 mmol, 98%), HOBt (7.0 mg, 0.05 mmol, 97%), Et_3_N (27.9 µL, 0.20 mmol), and MeCN (2 mL) were used according to the general procedure for peptide coupling. Preparative thin-layer chromatography afforded Phen-Ala-Gly-Gly-Boc (**17**) as a light-yellow solid (22.7 mg, 89%).

M.p. = 94–95 °C; R*_f_* = 0.4 (CH_2_Cl_2_:MeOH/9:1); ^1^H NMR (CDCl_3_) δ/ppm: 8.51 (d, *J* = 8.5 Hz, 1H, Phen), 8.46 (d, *J* = 8.1 Hz, 1H, Phen), 8.05 (d, *J* = 8.1 Hz, 1H, Phen), 7.95 (s, 1H, Phen), 7.70–7.64 (m, 1H, Phen), 7.63–7.59 (m, 2H, Phen), 7.14 (bd, *J* = 7,4 Hz, 1H, NH), 7.03 (bs, 1H, NH), 5.31 (s, 1H, NH), 4.99–4.92 (m, 1H, CH), 3.99–3.92 (m, 1H, CH_2_), 3.90–3.83 (m, 1H, CH_2_), 3.74–3.67 (m, 5H, OCH_3_, CH_2_), 3.42–3.35 (m, 1H, CH_2_), 3.31–3.24 (m, 1H, CH_2_), 2.99 (s, 3H, CH_3_), 1.41 (s, 9H, CH_3_-Boc); ^13^C NMR (CDCl_3_) δ/ppm: 171.18 (C_q_), 169.67 (C_q_), 168.19 (C_q_), 158.03 (C_q_), 143.04 (C_q_), 134.80 (C_q_), 131.19 (CH), 131.04 (C_q_), 128.74 (CH), 128.12 (CH), 126.35 (CH), 125.93 (CH), 125.43 (C_q_), 123.02 (C_q_), 122.21 (CH), 121.41 (CH), 79.96 (C_q_-Boc), 53.04 (CH or OCH_3_), 52.00 (CH or OCH_3_), 43.76 (CH_2_), 42.37 (CH_2_), 37.47 (CH_2_), 27.76 (CH_3_-Boc), 22.79 (CH_3_); M_w_ = 508.57; ESI-MS: *m*/*z* 509.2 [M+H]^+^.

3. Synthesis of Phen-Ala-Gly-Gly-GCP-Boc (**18**):

Phen-Ala-Gly-Gly-Boc ((**17**), 22.7 mg, 0.04 mmol), Boc-GCP-COOH ((**7**), 15.9 mg, 0.04 mmol), HBTU (15.5 mg, 0.04 mmol, 98%), HOBt (5.6 mg, 0.04 mmol, 97%), Et_3_N (44.6 µL, 0.32 mmol), and MeCN (2 mL) were used according to the general procedure for peptide coupling. Preparative thin-layer chromatography afforded Phen-Ala-Gly-Gly-GCP-Boc (**18**) as a light-yellow solid (21.7 mg, 71%).

M.p. = 160–163 °C; R*_f_* = 0.4 (CH_2_Cl_2_:MeOH/9:1); ^1^H NMR (CDCl_3_) δ/ppm: 10.57 (bs, 1H, NH), 9.06 (bs, 2H, NH), 8.62–8.17 (m, 3H, NH, Phen), 8.11–7.36 (m, 8H, NH, Phen), 6.76–6.42 (m, 2H, Pyrr), 4.92 (s, 1H, CH), 4.15–3.50 (m, 7H, CH_2_, OCH_3_), 3.39–3.10 (m, 2H, CH_2_), 2.87 (s, 3H, CH_3_), 1.44 (s, 9H, CH_3_-Boc); ^13^C NMR (CDCl_3_) δ/ppm: 158.02 (C_q_), 142.86 (C_q_), 134.84 (C_q_), 131.12 (CH), 130.82 (C_q_), 128.49 (CH), 127.96 (CH), 126.19 (CH), 125.78 (CH), 125.24 (C_q_), 122.94 (C_q_), 122.03 (CH), 121.38 (CH), 78.21 (C_q_-Boc), 53.09 (CH), 52.01 (OCH_3_), 42.67 (CH_2_), 42.39 (CH_2_), 37.23 (CH_2_), 27.47 (CH_3_-Boc), 22,59 (CH_3_); M_w_ = 686.71; ESI-MS: *m*/*z* 687.3 [M+H]^+^.

4. Synthesis of Phen-Ala-Gly-Lys-GCP-Boc (**20**):

Phen-Ala ((**15)**, 3.4 mg, 0.01 mmol), HOOC-Gly-Lys-GCP-Boc ((**1a**), 5.0 mg, 0.01 mmol), HBTU (3.3 mg, 0.01 mmol, 98%), HOBt (1.6 mg, 0.01 mmol, 97%), Et_3_N (10.0 µL, 0.07 mmol), and MeCN (1 mL) were used according to the general procedure for peptide coupling. Preparative thin-layer chromatography afforded Phen-Ala-Gly-Lys-GCP-Boc (**20**) as a light-yellow solid (4.9 mg, 66%).

M.p. = 122–123 °C; R*_f_* = 0.4 (CH_2_Cl_2_:MeOH/9:1); ^1^H NMR (DMSO-*d*_6_) δ/ppm: 11.59 (bs, 1H, NH), 10.83 (bs, 1H, NH), 9.31 (bs, 1H, NH), 8.78–8.62 (m, 2H, H10, H1), 8.61–8.27 (m, 3H), 8.26–8.05 (m, 2H), 7.98 (d, *J* = 7.9 Hz, 1H), 7.85–7.61 (m, 3H), 6.93—6.63 (m, 3H), 4.76–4.61 (m, 1H, CH-Ala), 4.34 (s, 1H, CH-Lys), 3.84–3.53 (m, 5H, CH_2_-Gly, OCH_3_), 3.47–3.16 (m, 1H, CH_2_-Ala), 3.02–2.81 (m, 6H, CH_2_-Ala, CH_2_-Lys, CH_3_), 1.66 (bs, 2H, CH_2_-Lys), 1.55–1.05 (m, 22H, CH_2_-Lys, CH_3_-Boc); ^13^C NMR (DMSO-*d*_6_) δ/ppm: 172.12 (C_q_), 172.01 (C_q_), 171.71 (C_q_), 171.69 (C_q_), 168.88 (C_q_), 168.83 (C_q_), 159.71 (C_q_), 158.57 (C_q_), 158.46 (C_q_), 158.41 (C_q_), 155.52 (C_q_), 143.07 (C_q_), 136.85 (C_q_), 132.16 (Phen), 130.47 (C_q_), 128.87 (Phen), 128.46 (Phen), 126.91 (Phen), 126.38 (Phen), 125.24 (C_q_), 123.19 (C_q_), 122.63 (Phen), 122.46 (Phen), 113.12 (Pyrr), 77.29 (C_q_-Boc), 55.75 (CH or OCH_3_), 53.33 (CH or OCH_3_), 51.98 (CH or OCH_3_), 41.61 (CH_2_), 36.73 (CH_2_), 31.26 (CH_2_), 29.20 (CH_2_), 28.23 (CH_3_-Boc), 27.76 (CH_3_-Boc), 23.02 (CH_3_), 22.88 (CH_2_); M_w_ = 857.95; ESI-MS: *m*/*z* 858.6 [M+H]^+^.

5. Synthesis of Phen-Ala-Lys-Gly-GCP-Boc (**21**):

Phen-Ala ((**15**), 10.0 mg, 0.03 mmol), HOOC-Lys-Gly-GCP-Boc ((**1b**), 14.7 mg, 0.03 mmol), HBTU (9.8 mg, 0.03 mmol, 98%), HOBt (3.5 mg, 0.03 mmol, 97%), Et_3_N (21.2 µL, 0.15 mmol), and MeCN (2 mL) were used according to the general procedure for peptide coupling. Preparative thin-layer chromatography afforded Phen-Ala-Lys-Gly-GCP-Boc (**21**) as a light-yellow solid (3.2 mg, 15%).

M.p. = 137–138 °C; R*_f_* = 0.4 (CH_2_Cl_2_:MeOH/9:1); ^1^H NMR (DMSO-*d*_6_) δ/ppm: 9.25 (bs, 1H), 8.80–8.37 (m, 5H, H10, Phen, NH), 8.16 (s, 1H, Phen), 8.04–7.89 (m, 2H), 7.88–7.52 (m, 4H), 6.83–6.56 (m, 3H), 4.75–4.56 (m, 1H, CH-Ala), 4.36–4.21 (m, 1H, CH-Lys), 3.90–3.76 (m, 2H, CH_2_), 3.68–3.55 (m, 4H, CH_2_, OCH_3_), 2.94 (s, 3H, CH_3_), 2.86–2.75 (m, 2H, CH_2_-Lys), 1.73–0.70 (m, 24H, CH_2_-Lys, CH_3_-Boc); ^13^C NMR (DMSO-*d*_6_) δ/ppm: 171.73 (C_q_), 171.64 (C_q_), 158.70 (C_q_), 158.52 (C_q_), 155.49 (C_q_), 143.05 (C_q_), 136.97 (C_q_), 132.31 (Phen), 130.49 (C_q_), 128.84 (Phen), 128.42 (Phen), 126.82 (Phen), 126.36 (Phen), 125.27 (C_q_), 123.21 (C_q_), 122.60 (Phen), 122.51 (Phen), 113.56 (Pyrr), 112.59 (Pyrr), 77.29 (C_q_-Boc), 55.75 (CH or OCH_3_), 53.47 (CH or OCH_3_), 51.99 (CH or OCH_3_), 42.10 (CH_2_), 36.54 (CH_2_), 31.69 (CH_2_), 29.22 (CH_2_), 28.25 (CH_3_-Boc), 27.91 (CH_3_-Boc), 23.03 (CH_3_), 22.40 (CH_2_); M_w_ = 857.95; ESI-MS: *m*/*z* 858.5 [M+H]^+^.

#### 3.1.4. General Procedure for –Boc Deprotection and Synthesis of Hydrochloride Salts **19**, **22**, and **23** (Figure 12)

To the solution of the corresponding Boc-protected amino acid derivative in DCM (2 mL) was added a mixture of TFA and water (TFA:H_2_O/9:1, 1 mL). The reaction mixture was stirred overnight at room temperature. The removal of the solvent under reduced pressure afforded a triflouroacetate salt of the corresponding amine, which was purified by preparative thin-layer chromatography (DCM:MeOH/9:1) and obtained as a colorless oil. The obtained compound was dissolved in MeOH (2 mL) to which was added 5% HCl (2 mL) followed by stirring at room temperature for 1h. Evaporation of the solvent afforded hydrochloride salt of a desired compound as a light-yellow solid.

1. Synthesis of Phen-Ala-Gly-Gly-GCP (**19**):

Phen-Ala-Gly-Gly-GCP-Boc ((**18**), 21.7 mg, 0.03 mmol) was treated according to the general procedure for –Boc deprotection and synthesis of hydrochloride salts, to afford Phen-Ala-Gly-Gly-GCP (**19**) as a yellow oil (14.4 mg, 70%).

IR (KBr) *ν*_max_/cm^−1^: 3499 (s), 2980 (w), 2922 (w), 1701 (s), 1624 (s), 1660 (m), 1475 (w), 1447 (w), 1410 (w), 1366 (vw), 1286 (m), 1254 (m), 1203 (w), 1090 (w), 854 (vw), 812 (vw), 762 (w), 515 (m); ^1^H NMR (DMSO-*d*_6_) δ/ppm: 12.36 (s, 1H, NH), 11.88 (s, 1H, NH), 8.96–8.78 (m, 3H), 8.66–8.21 (m, 7H), 8.13 (s, 1H), 7.98–7.82 (m, 2H), 7.44 (s, 1H, Pyrr), 6.85 (s, 1H, Pyrr), 4.77–4.67 (s, 1H, CH), 3.86 (s, 2H, CH_2_), 3.75–3.61 (m, 5H, OCH_3_, CH_2_), 3.31–3.17 (m, 5H, CH_3_, CH_2_); ^13^C NMR (DMSO-*d*_6_) δ/ppm: 171.45 (Cq), 168.97 (Cq), 168.82 (Cq), 159.50 (Cq), 155.27 (Cq), 149.11 (Cq), 132.23 (Cq), 125.47 (Cq), 124.10 (Cq), 123.62 (Cq), 123.38 (Phen), 123.18 (Phen), 115.70 (Pyrr), 112.81 (Pyrr), 53.02 (CH or OCH_3_), 52.02 (CH or OCH_3_), 42.13 (CH_2_), 41.58 (CH_2_), 36.23 (CH_2_); HRMS: *m*/*z*: 587.2366 [*M*+H]^+^; calculated for C_29_H_30_N_8_O_6_^+^: 587.2367.

2. Synthesis of Phen-Ala-Gly-Lys-GCP (**22**):

Phen-Ala-Gly-Lys-GCP-Boc ((**20**), 19.6 mg, 0.02 mmol) was treated according to the general procedure for –Boc deprotection and synthesis of hydrochloride salts, to afford Phen-Ala-Gly-Lys-GCP (**22**) as a yellow solid (15.4 mg, 88%).

M.p. > 300 °C; IR (KBr) *ν*_max_/cm^−1^: 3423 (s), 2974 (w), 2922 (w), 2858 (w), 1641 (s), 1626 (s), 1555 (m), 1474 (w), 1439 (w), 1377 (w), 1352 (w), 1271 (w), 1254 (w), 1134 (m), 1076 (m), 1051 (m), 922 (w), 889 (w), 856 (vw), 816 (w), 768 (w), 725 (m), 631 (w), 617 (w), 555 (w), 471 (m); ^1^H NMR (DMSO-*d*_6_) δ/ppm: 12.54–12.40 (m, 1H, NH-Pyrr), 12.06 (s, 1H, NH), 8.90 (s, 2H), 8.78–8.61 (m, 3H), 8.57–8.28 (m, 6H), 8.13 (bs, 1H), 8.01–7.79 (m, 5H), 7.57–7.45 (m, 1H, Pyrr), 6.91–6.78 (m, 1H, Pyrr), 4.80–4.55 (m, 1H, CH-Ala), 4.45–4.20 (m, 1H, CH-Lys), 3.83–3.56 (m, 6H, CH_2_-Ala, CH_2_-Gly, OCH_3_), 3.25 (s, 3H), 2.74 (s, 2H, CH_2_-Lys), 1.82–1.10 (m, 6H, CH_2_-Lys); ^13^C NMR (DMSO-*d*_6_) δ/ppm: 172.56 (C_q_), 172.00 (C_q_), 171.94 (C_q_), 171.59 (C_q_), 169.34 (C_q_), 169.00 (C_q_), 168.83 (C_q_), 159.71 (C_q_), 159.24 (C_q_), 155.48 (C_q_), 132.12 (C_q_), 130.51 (Phen), 125.62 (C_q_), 124.16 (C_q_), 123.75 (C_q_), 123.56 (Phen), 123.31 (Phen), 115.70 (Pyrr), 113.77 (Pyrr), 53.19 (CH or OCH_3_), 52.99 (CH or OCH_3_), 52.19 (CH or OCH_3_), 41.67 (CH_2_), 38.57 (CH_2_), 36.25 (CH_2_), 30.86 (CH_2_), 26.59 (CH_2_), 22.48 (CH_2_); HRMS: *m*/*z*: 658.3099 [*M*+H]^+^; calculated for C_33_H_39_N_9_O_6_^+^: 658.3102.

3. Synthesis of Phen-Ala-Gly-Gly-GCP (**23**):

Phen-Ala-Lys-Gly-GCP-Boc ((**21**), 11.6 mg, 0.01 mmol) was treated according to the general procedure for –Boc deprotection and synthesis of hydrochloride salts, to afford Phen-Ala-Lys-Gly-GCP (**23**) as a yellow solid (7.2 mg, 95%).

M.p. > 300 °C; IR (KBr) *ν*_max_/cm^−1^: 3429 (s), 2966 (w), 2922 (w), 2858 (w), 1641 (s), 1626 (s), 1560 (m), 1474 (w), 1439 (w), 1377 (w), 1352 (w), 1290 (w), 1134 (m), 1076 (m), 922 (w), 887 (w), 856 (w), 816 (w), 756 (w), 725 (m), 631 (m), 613 (m), 559 (m); ^1^H NMR (DMSO-*d*_6_) δ/ppm: 12.33 (s, 1H, NH-Pyrr), 12.09 (s, 1H, NH), 8.99–8.86 (m, 2H), 8.85–8.62 (m, 4H), 8.60–8.45 (m, 3H), 8.38 (bs, 1H), 8.23–8.10 (m, 8.1 Hz, 2H), 8.02–7.80 (m, 5H), 7.51 (s, 1H, Pyrr), 6.80 (s, 1H, Pyrr), 4.77–4.54 (m, 1H, CH-Ala), 4.35–4.16 (m, 1H, CH-Lys), 3.88–3.66 (m, 4H, CH_2_), 3.65 (s, 3H, OCH_3_), 3.27 (m, 3H, CH_3_), 2.71 (s, 2H, CH_2_-Lys), 1.71–0.70 (m, 6H); ^13^C NMR (DMSO-*d*_6_) δ/ppm: 172.52 (C_q_), 171.80 (C_q_), 171.66 (C_q_), 171.55 (C_q_), 169.26 (C_q_), 168.63 (C_q_), 159.59 (C_q_), 159.41 (C_q_), 155.52 (C_q_), 132.26 (C_q)_, 131.59 (Phen), 125.57 (C_q_), 123.74 (C_q_), 123.55 (Phen), 123.29 (Phen), 115.95 (Pyrr), 112.81 (Pyrr), 53.23 (CH or OCH_3_), 52.88 (CH or OCH_3_), 52.12 (CH or OCH_3_), 42.07 (CH_2_), 38.47 (CH_2_), 36.47 (CH_2_), 31.11 (CH_2_), 26.47 (CH_2_), 22,10 (CH_2_); HRMS: *m*/*z*: 658.3097 [*M*+H]^+^; calculated for C_33_H_39_N_9_O_6_^+^: 658.3102.

### 3.2. Spectrophotometric Measurements

All materials were obtained from commercial suppliers and were used without purification unless noted otherwise. ^1^H NMR and ^13^C NMR spectra were recorded on a Bruker Avance 300 or Bruker Avance 600 (at 300 and 600 MHz) (Bruker Corporation, Billerica, MA, USA) at 25 °C. Chemical shifts (d) were provided in parts per million (ppm) with respect to tetramethylsilane used as an internal standard, while coupling constants (J) were expressed in hertz. The ^1^H NMR spectra display the splitting patterns represented as follows: s (singlet), brs (broad singlet), d (doublet), t (triplet), and m (multiplet). Melting points were measured using a Kofler melting points apparatus from Reichert, Austria, and remained uncorrected. Infrared spectra were measured on an ABB Bomem MB102 single-beam spectrometer manufactured in Quebec City, Canada. The spectral bands are expressed in “wave numbers” with the unit cm^−1^. A MALDI TOF/TOF 4800 plus analyzer, Applied Biosystems (Mundelein, IL, USA) was used to record mass spectra. A Varian Cary 100 Bio spectrophotometer (Agilent, Santa Clara, CA, USA), a JASCO J815 spectrophotometer (ABL&E Handels GmbH, Wien, Austria), and a Varian Cary Eclipse spectrophotometer (Agilent, Santa Clara, CA, USA) were used to record the absorption, CD, and fluorescence spectra, respectively. Measurements were performed at 25 °C using 1cm path quartz cuvettes. 

Polynucleotides were purchased as noted: poly dA, poly dT, poly A-poly U, calf thymus (ct)-DNA, poly(dGdC)_2_, poly(dAdT)_2_, poly dA-poly dT (Sigma-Aldrich, St. Louis, MO, USA, ≥98.0%). 

The ATT triplex formation involved mixing the three constituent strands in sodium cacodylate buffer (*I* = 0.05 mol dm^−3^, pH 7.0), supplemented with NaCl (0.05 M) and 1 mM EDTA (≥99.0%). The mixture was then heated to 90 °C for 15 min and gradually cooled to 10 °C [38,40].

Polynucleotides were prepared in Na–cacodylate buffer, *I* = 0.05 mol dm^−3^, pH 7.0. The calf thymus DNA underwent additional sonication and was filtered through a 0.45 mm filter [55]. The concentration of polynucleotides was determined spectroscopically [56,57] based on the phosphate concentration. Spectrophotometric titrations were conducted at both pH 7.0 and 5.0 (*I* = 0.05 mol dm^−3^, sodium cacodylate buffer). For fluorimetric experiments, portions of polynucleotide solution were added to the solution containing the studied compound. For CD experiments, portions of the compound’s stock solution were added to the polynucleotide solution.

Fluorimetric experiments utilized an excitation wavelength of λ_exc_ = 300 nm to prevent the inner filter effect resulting from the rising absorbance of the polynucleotide. Emission was recorded within the range of λ_em_ = 350–600 nm. The majority of *K*_a_ values, derived by analyzing titration data through the Scatchard [34,35] equation (Table 2), had satisfactory correlation coefficients (>0.99). Thermal melting curves for DNA, RNA, and their complexes with the studied compounds were generated by monitoring the absorption change at 260 nm with respect to temperature [36]. The absorbance of the ligands was subtracted from each curve, and the absorbance scale was normalized. *T*_m_ values represent the midpoints of the transition curves, determined from the peak of the first derivative and validated graphically using the tangent method. The Δ*T*_m_ values were calculated by subtracting the *T*_m_ of the free nucleic acid from the *T*_m_ of the complex. Every Δ*T*_m_ value here reported was the average of at least two measurements. The error in Δ*T*_m_ is ±0.5 °C. 

#### Mass Spectrometry Experimental for Section 2.2.2

The solvents used for the spectroscopic measurements were HPLC or spectroscopic-grade (Sigma-Aldrich Chemie GmbH, Steinheim, Germany) and were used without further purification. 

The self-assembly of compounds **19**, **22**, and **23** was studied by ESI-MS/MS. The spectra were obtained at the same concentrations and conditions in positive ion (ES^+^) mode. Certain fragmentations occurred at higher collision energies. The fragmentation pathways for all analyzed compounds were proposed based on MS/MS spectra of protonated molecular ions [M+H]^+^ and sodium adducts.

The mass spectral data were acquired using an Agilent 6550 Series Accurate-Mass-Quadrupole Time-of-Flight (Q-TOF) equipped with an electrospray ionization interface operated in the ES^+^ (Agilent Technologies, Palo Alto, CA, USA). The samples were prepared in methanol (MeOH), H_2_O, D_2_O, and acetonitrile to a concentration of about 0.05 mg/mL. The infusion into the mass spectrometer was performed at a flow rate of 0.4 mL/min using MeOH as the mobile phase. Nitrogen was used as an auxiliary (35 psi) and sheath gas (350 °C and 11 L min^−1^). The other parameters were as follows: capillary voltage (VCap) 3000V and 4000; nozzle voltage = 0 V; and the drying gas temperature was set at 200 °C. The full mass spectra were acquired over the mass range *m*/*z* 10-3000. For data analysis, Mass Hunter Qualitative Analysis Navigator B 08.00 was used. A parent ion window of typically 2 amu (i.e., parent mass ± 1 amu) was chosen to perform further MS/MS experiments.

### 3.3. MTT Assay

#### 3.3.1. Cell Line

The human cervical carcinoma HeLa cell line was obtained from a cell culture bank (GIBCO BRL, Invitrogen, Carlsbad, CA, USA). For the cytotoxicity assay, cells at 35 and 40 population doublings were employed. These cells were cultured as a monolayer culture in Dulbecco’s modified Eagle’s medium (DMEM; Sigma-Aldrich, St. Louis, MO, USA), supplemented with 10% fetal bovine serum (FBS; Sigma-Aldrich). The cultures were maintained in a humidified atmosphere containing 5% CO_2_ at 37 °C, and subculturing was performed every 3–4 days.

#### 3.3.2. Cytotoxicity Assay

HeLa cells were seeded in 96-well plates. Twenty-four hours later, the cells were treated, in quadruplicate, with different concentrations of compounds (dissolved in DMSO and kept at 4 °C). Following 72 h of incubation at 37 °C, the medium was aspired and a modified 3-(4,5-dimethylthiazol-2-yl)-2,5-diphenyltetrazolium bromide (MTT) assay [58] was used to determine the cytotoxic effect of tested compounds. Three hours later, formazan crystals were dissolved in DMSO, the plates were mechanically agitated for 5 min, and the optical density at 545 nm was determined on a microtiter plate reader (Awareness Technology Inc., Palm City, FL, USA). The cytotoxic effect of the compounds on MES-OV cells was measured by the alamarBlue assay (ThermoFischer Scientific, Waltham, MA, USA). The experiments were repeated at least three times.

### 3.4. Computational Details

To parameterize all investigated derivatives for classical molecular dynamics (MD) simulations, their geometries were first optimized with the Gaussian 16 program [59] at the (SMD)/B3LYP/6–31+G(d) level in water, while their RESP atomic charges were subsequently calculated by the (SMD)/HF/6–31+G(d) model. Such obtained geometries were solvated in a truncated octahedral box with TIP3P waters spanning 30 Å thick buffers. Each system was neutralized with the appropriate number of Cl^−^ ions to maintain the net zero charge and allow for simulations under periodic boundary conditions. These were distributed around the system using the Monte Carlo method as implemented within the CHARMM-GUI server. The prepared simulation boxes were submitted to geometry optimization in the GROMACS 2022.5 program [60]. Optimized systems were gradually heated from 0 to 300 K and equilibrated during 250 ps using NVT conditions and then submitted to 300 ns of productive and unconstrained MD simulations employing a time step of 2 fs at a constant pressure (1 atm) and temperature (300 K). The long-range electrostatic interactions were calculated by the Particle Mesh Ewald method [61] and were updated in every second step, while the non-bonded interactions were truncated at 10.0 Å. The obtained structures in the matching trajectories were clustered utilizing the pertinent modules within the GROMACS software suite. The idea behind this computational strategy was to investigate whether the intrinsic dynamical features of the investigated systems both affect and can explain their tendency to recognize polynucleotides, which also avoids difficulties and inaccuracies linked with the computational prediction of single-stranded polynucleotide structures, as recently highlighted by Jeddi and Saiz [62]. The mentioned approach turned out to be very useful in interpreting the affinities of several conjugates towards single-stranded RNA systems [18,53,54].

To verify that our clustering analysis correctly identified the most representative structures of each derivative at both considered pH values, we proceeded by computing energies of the excited states determining the experimental UV/Vis spectra for isolated conjugates in water. To do so, we used the most prevailing structure of each conjugate and optimized its geometry with the (SMD)/B3LYP/6–31+G(d) model in water. This was followed by TD-DFT computations at the same level of theory considering the 64 lowest singlet electronic excitations. The choice of this setup was prompted by its recent success in modeling UV/Vis spectra of organic heterocycles in various solvents [63,64].

## 4. Conclusions

Three new phenanthridine tetrapeptides (two with lysine and one without it) were prepared to find new spectrophotometric probes for DNA and RNA. In the synthesis of Phen-GCP tetrapeptides, we used two pathways, either functionalization of Phen-Ala with Gly-Gly linker and subsequent amide coupling with GCP-carboxylic acid or amide coupling of Phen-Ala with a preconstructed guanidinocarbonylpyrrole building block (Gly-Lys-GCP or Lys -Gly-GCP). Similar to the first generation (phenanthridine tripeptides), which did not contain lysine in the chain, and based on the results (hypochromic effect and the rise in absorption after the temperature increase at the maximum of 300 nm, the bisignate shape of CD spectra) and structure (sufficiently long and flexible bridge between phenanthridine and pyrrole in **19**, **22**, and **23**), the existence of the interaction between the GCP unit and phenanthridine can be assumed in this series, most likely between the phenanthridine and pyrrole chromophores. The intramolecular interactions were further confirmed by mass spectroscopy and computational analysis. Even though the probability of forming hydrophobic aromatic interactions is lower in the gas phase, we found a signal indicating the formation of a dimer of **23**.

DNA and RNA studies suggested that the interaction of new compounds was influenced by (a) the type and arrangement of amino acids in the peptide linker and (b) the solution pH value, which determines the protonation state of molecules and the number of positive charges. Also, the composition of polynucleotide base pairs affected the strength and mode of small molecule binding. The substitution of one glycine by lysine in **22** and **23** enabled stronger binding interactions to DNA and RNA relative to compound **19** with two glycines. The binding constants were slightly lower in weakly acidic conditions, while polynucleotide stabilization was mainly better under the same conditions. Regioisomer **23** (with lysine closer to the phenanthridine ring) showed a dual and selective fluorimetric response only in interaction with non-alternating AT (pdApdT) and ATT polynucleotides at pH 5.0. Additionally, this dual binding mode of **23** was confirmed by the induction of triplex formation from double-stranded poly dA-poly dT, both under neutral and weakly acidic conditions. It is worth noting that compound **23** caused fluorescence enhancement only when interacting with non-alternating AT base pairs. At pH 5.0, compound **23** demonstrated a selective and stronger stabilization of the ATT triplex compared with the AT duplex. 

Moreover, **23** exhibited the capability to differentiate between the binding sites of ds-DNA and ds-RNA by detecting distinct intensities of the ICD signal at 285 nm. This signal was prominent for DNA-induced CD bands and insignificant for RNA-ICD bands. 

In addition, **23** increased the intensity of the polynucleotide maximum at 275 nm exclusively with poly(dGdC)_2_ and GC-containing polynucleotide (ctDNA). Hence, isomer **23** could be utilized as a straightforward small molecule chiral probe for GC-rich DNA. All the studied substances did not show in vitro toxicity (Figure 10), which is an advantageous quality for potential spectrophotometric probes.

Computational analysis revealed the strong tendency of all derivatives to engage in favorable π–π interactions involving phenanthridine and pyrrole fragments, thus assuming bent conformations. This agrees with the temperature-dependent UV/Vis measurements that also support the existence of intramolecular stacking interactions between these two chromophores. In line with our earlier work and results reported here, the dominance of such bent geometries hinders efficient sensing of polynucleotides as it lowers the overall availability of phenanthridine for interacting with the latter. In **19**, the uncharged Gly-Gly linker allows high flexibility of the system, which promotes favorable bent conformations that are almost exclusively present at both pH conditions, which is why no activities towards polynucleotides were observed. Given its bulkier size and charged nature, the lysine residue within the linker reduces the system flexibility and hinders stacking contacts, thereby allowing its phenanthridine to become more exposed and likelier to recognize polynucleotides in solution. The position of lysine within conjugate **23** appears more optimal than in **22**, as seen in a larger percentage of non-stacked conformations at both pH values. This promotes the former as the most efficient polynucleotide sensor evaluated here, thus confirming experimental observations. The validity of computational results is demonstrated by accurately reproducing the UV/Vis spectra of isolated conjugates while underlying only the modest sensitivity of these electronic transitions towards changes in open-to-bent conformations. 

## Figures and Tables

**Table 1:** Electronic absorption maxima and corresponding molar extinction coefficients ^a,b^ and fluorescence maxima.

UV/Vis			Fluorescence
λ_max_ (nm)/ε × 10^3^ (dm^3^ mol^−1^ cm^−1^)			Emission
	pH 5.0	pH 7.0	λ_max_ (nm)
**22**	252/43.6 298/21.4	251/48.3 300/23.3	397 ^a^/372 ^b^
**23**	252/46.7 297/24.1	251/44.1 297/22.5	397 ^a^/372 ^b^
**Phen-AA**	251/49.5 300/4.8	250/50.6 300/4.5	402 ^a^/370 ^b^
**Phen-Gly**	251/51.9 300/5.1	250/53.7 300/4.8	403 ^a^/370 ^b^
**GCP**	250/4.0 297/27.3	250/6.1 297/28.4	-

^a^ Buffer pH 5.0 ^b^ Buffer pH 7.0 (sodium cacodylate buffer, *I* = 0.05 moldm^−3^).

**Table 2:** Binding constants (log *K*_a_) ^a^ and ratios *n* ^a^ ([bound compound]/[polynucleotide phosphate]) calculated from the fluorescence titrations of **22** and **23** with ds-polynucleotides at pH 7.0 and pH 5.0 (buffer sodium cacodylate, *I* = 0.05 mol dm^−3^).

pH		ctDNA			p(dAdT)_2_			p(dGdC)_2_			pApU			pdApdT		
		log *K*_a_	*n*	I/I_0_ ^b^	log *K*_a_	*n*	I/I_0_ ^b^	log *K*_a_	*n*	I/I_0_ ^b^	log *K*_a_	*n*	I/I_0_ ^b^	log *K*_a_	*n*	I/I_0_ ^b^
7.0	**22**	- ^c^	- ^c^	- ^c^	6.7	0.3	0.9	6.1	0.8	0.9	- ^c^	- ^c^	- ^c^	- ^c^	- ^c^	- ^c^
	**23**	7.0	0.5	0.8	6.7	0.2	0.9	5.8	0.4	0.6	7.1	0.3	0.8	6.6	0.3	0.9
5.0	**22**	5.7	0.3	0.7	5.3	0.5	0.6	5.3	0.4	0.3	5.5	0.5	0.8	- ^c^	- ^c^	- ^c^
	**23**	5.5	0.5	0.8	6.0	0.2	0.6	5.3	0.3	0.3	5.8	0.2	0.8	6.3 ^d^ 7.4 ^e^	0.5 0.4	0.8 2.1

^a^ Analysis of measured data using the Scatchard equation [34,35] yielded values of the ratio *n*[bound ligand]/[polynucleotide]; the correlation coefficients were >0.99 for the majority of calculated *K*_a_; ^b^ *I*_0_—initial emission intensity of **22** and **23**; *I*—emission intensity of **22**, **23**/polynucleotide complex calculated by the Scatchard equation. ^c^ Small emission change or insufficient data points in the non-linear part of the curve hindered the calculation of the association constant. ^d^ Data calculated from the part of the titration experiment (λ_max_ = 406 nm) with poly dA-poly dT in which the starting fluorescence of **23** was quenched. ^e^ Data calculated from the part of the titration experiment (λ_max_ = 453 nm) with poly dA-poly dT in which the fluorescence of the primarily formed complex was enhanced by the formation of the secondary complex.

**Table 3:** The ^a^ Δ*T*_m_ values (°C) of studied ds-polynucleotides upon addition of ratio ^b^ **r** = 0.3 of **19**, **22**, and **23** at pH 7.0 and 5.0 (sodium cacodylate buffer, *I* = 0.05 mol dm^−3^).

	^b^ r = 0.3	ctDNA	pApU	pdApdT	p(dAdT)_2_
pH 7.0	**19**	0.6	0	0.7	1.2
	**22**	0.9	0	1.5	2.6
	**23**	0.7	2.5	-/2.1 ^c^	1.0
pH 5.0	**19**	1.3	1.1/1.0 ^d^	0.5	1.3
	**22**	1.2	0/0.8 ^d^	1.3	3.9
	**23**	1.3	0/0.8 ^d^	-/0.7 ^c^	3.0

^a^ Error in ΔT_m_: ±0.5 °C; ^b^ **r** = [compound]/[polynucleotide]; ^c^ biphasic transitions; ^d^ biphasic transitions: the first transition at *T*_m_ = 47 °C relates to the melting of poly A-poly Um and the second transition at *T*_m_ = 61 °C relates to the melting of poly AH^+^-poly AH^+^ since poly A at pH 5.0 is mostly protonated and constitutes the ds-polynucleotide [37].

**Table 4:** The ^a^ *T*_m_ values and Δ*T*_m_ values (°C) of pdApdT duplex and dAdTdT triplex upon addition of ratio ^b^ **r** = 0.3 of **23** at pH 7.0 and pH 5.0 (sodium cacodylate buffer, *I* = 0.1 mol dm^−3^).

^b^ r = 0.3	pdApdT DUPLEX		ATT TRIPLEX	
	*T*_m_	Δ*T*_m_	*T*_m_	Δ*T*_m_
pH 5.0	70.1	-	23.8/70.3 ^c^	-
	27.3/70.2	-/0.1	29.3/70.0	5.5/0.3
pH 7.0	70.8	-	25.1/71.3 ^c^	-
	71.3	0.5	26.2/71.1	1.1/−0.2

^a^ Error in ΔT_m_: ±0.5 °C; ^b^ **r** = [compound]/[nucleotide phosphate]; c(polynucleotide) = 25.0 μM; c(**23**) = 7.5 μM; ^c^ biphasic transitions: the first transition at *T*_m_ = 23.8 and 25.1 °C at pH 5.0 and 7.0, respectively, refers to the melting of the dAdTdT triplex (ATT), and the second transition at *T*_m_ = 70.3 and 71.4 °C refers to the melting of pdApdT.

## Data Availability

The original contributions presented in the study are included in the article/Supplementary Materials, further inquiries can be directed to the corresponding author/s.

## Supplementary Materials

The following supporting information can be downloaded at: https://www.mdpi.com/article/10.3390/ijms25094866/s1.

## References

[1] Boutorine A.S., Novopashina D.S., Krasheninina O.A., Nozeret K., Venyaminova A.G. (2013). Fluorescent Probes for Nucleic Acid Visualization in Fixed and Live Cells. Molecules.

[2] Bao G., Rhee W.J., Tsourkas A. (2009). Fluorescent Probes for Live-Cell RNA Detection. Annu. Rev. Biomed. Eng..

[3] Shah A., Khan A., Qureshi R., Ansari F., Nazar M., Shah S. (2008). Redox Behavior of Anticancer Chalcone on a Glassy Carbon Electrode and Evaluation of its Interaction Parameters with DNA. Int. J. Mol. Sci..

[4] Dembska A., Juskowiak B. (2015). Pyrene functionalized molecular beacon with pH-sensitive i-motif in a loop. Spectrochim. Acta Part A: Mol. Biomol. Spectrosc..

[5] Fang M.X., Adhikari R., Bi J.H., Mazi W., Dorh N., Wang J.B., Conner N., Ainsley J., Karabencheva-Christova T.G., Luo F.T. (2017). Fluorescent probes for sensitive and selective detection of pH changes in live cells in visible and near-infrared channels. J. Mater. Chem. B.

[6] Michel B.Y., Dziuba D., Benhida R., Demchenko A.P., Burger A. (2020). Probing of Nucleic Acid Structures, Dynamics, and Interactions With Environment-Sensitive Fluorescent Labels. Front. Chem..

[7] Yang Y.F., Gao F.C., Wang Y.D., Li H., Zhang J., Sun Z.W., Jiang Y.Y. (2022). Fluorescent Organic Small Molecule Probes for Bioimaging and Detection Applications. Molecules.

[8] Tumir L.M., Stojkovic M.R., Piantanida I. (2014). Come-back of phenanthridine and phenanthridinium derivatives in the 21st century. Beilstein J. Org. Chem..

[9] Jothi D., Manickam S., Sawminathan S., Munusamy S., R S.K., Ashok Kumar S.K., Kulathu Iyer S. (2022). Phenanthridine-based fluorescence sensor for the “off-on” determination of thorium ion and its bio-imaging applications. Dye. Pigment..

[10] Sawminathan S., Munusamy S., Jothi D., Iyer S.K. (2021). Phenanthridine-Based Donor/Acceptor Fluorescent Dyes: Synthesis, Photophysical Properties and Fluorometric Sensing of Biogenic Primary Amines. ChemistrySelect.

[11] Dragan A.I., Bishop E.S., Strouse R.J., Casas-Finet J.R., Schenerman M.A., Geddes C.D. (2009). Metal-enhanced ethidium bromide emission: Application to dsDNA detection. Chem. Phys. Lett..

[12] Luedtke N.W., Liu Q., Tor Y. (2003). Synthesis, photophysical properties, and nucleic acid binding of phenanthridinium derivatives based on ethidium. Bioorg. Med. Chem..

[13] Luedtke N.W., Liu Q., Tor Y. (2005). On the electronic structure of ethidium. Chemistry.

[14] Kubař T., Hanus M., Ryjáček F., Hobza P. (2005). Binding of Cationic and Neutral Phenanthridine Intercalators to a DNA Oligomer Is Controlled by Dispersion Energy: Quantum Chemical Calculations and Molecular Mechanics Simulations. Chem. Eur. J..

[15] Parenty A.D.C., Smith L.V., Guthrie K.M., Long D.-L., Plumb J., Brown R., Cronin L. (2005). Highly Stable Phenanthridinium Frameworks as a New Class of Tunable DNA Binding Agents with Cytotoxic Properties. J. Med. Chem..

[16] Rangarajan S., Friedman S.H. (2007). Design, synthesis, and evaluation of phenanthridine derivatives targeting the telomerase RNA/DNA heteroduplex. Bioorg. Med. Chem. Lett..

[17] Tajmir-Riahi H.-A., Rájecký M., Šebrlová K., Mravec F., Táborský P. (2015). Influence of Solvent Polarity and DNA-Binding on Spectral Properties of Quaternary Benzo[c]phenanthridine Alkaloids. PLoS ONE.

[18] Matic J., Supljika F., Tandaric T., Duksi M., Piotrowski P., Vianello R., Brozovic A., Piantanida I., Schmuck C., Stojkovic M.R. (2019). DNA/RNA recognition controlled by the glycine linker and the guanidine moiety of phenanthridine peptides. Int. J. Biol. Macromol..

[19] Scholl M.T. (2007). Synthesis, Structural Characterization and Applications of Hyperbranched Polymers Based on L-Lysine.

[20] Arnon Z.A., Kreiser T., Yakimov B., Brown N., Aizen R., Shaham-Niv S., Makam P., Qaisrani M.N., Poli E., Ruggiero A. (2021). On-off transition and ultrafast decay of amino acid luminescence driven by modulation of supramolecular packing. iScience.

[21] Handula M., Chapeau D., Seimbille Y. (2023). Orthogonal synthesis of a versatile building block for dual functionalization of targeting vectors. Open Chem..

[22] Albert A., Goldacre R., Phillips J. (1948). 455. The strength of heterocyclic bases. J. Chem. Soc. (Resumed).

[23] Brandão-Lima L.C., Silva F.C., Costa P.V.C.G., Alves-Júnior E.A., Viseras C., Osajima J.A., Bezerra L.R., de Moura J.F.P., de A. Silva A.G., Fonseca M.G. (2021). Clay Mineral Minerals as a Strategy for Biomolecule Incorporation: Amino Acids Approach. Materials.

[24] Schmuck C., Bickert V., Merschky M., Geiger L., Rupprecht D., Dudaczek J., Wich P., Rehm T., Machon U. (2007). A Facile and Efficient Multi-Gram Synthesis of *N*-Protected 5-(Guanidinocarbonyl)-1*H*-pyrrole-2-carboxylic Acids. Eur. J. Org. Chem..

[25] Berova N. (1997). Koji Nakanishi’s enchanting journey in the world of chirality. Chirality.

[26] Berova N., Bari L.D., Pescitelli G. (2007). Application of electronic circular dichroism in configurational and conformational analysis of organic compounds. Chem. Soc. Rev..

[27] Gargiulo D., Derguini F., Berova N., Nakanishi K., Harada N. (2002). Unique ultraviolet-visible and circular dichroism behavior due to exciton coupling in a biscyanine dye. J. Am. Chem. Soc..

[28] Baytekin B., Baytekin H.T., Schalley C.A. (2006). Mass spectrometric studies of non-covalent compounds: Why supramolecular chemistry in the gas phase?. Org. Biomol. Chem..

[29] Schalley C.A. (2000). Supramolecular chemistry goes gas phase: The mass spectrometric examination of noncovalent interactions in host-guest chemistry and molecular recognition. Int. J. Mass Spectrom..

[30] Schalley C.A. (2001). Molecular recognition and supramolecular chemistry in the gas phase. Mass Spectrom. Rev..

[31] Springer A., Schalley C.A. (2009). Mass Spectrometry and Gas-Phase Chemistry of Non-Covalent Complexes.

[32] Saenger W. (1984). Principles of Nucleic Acid Structure.

[33] Wilson W.D., Wang Y.H., Krishnamoorthy C.R., Smith J.C. (2002). Poly(dA).cntdot.poly(dT) exists in an unusual conformation under physiological conditions: Propidium binding to poly(dA).cntdot.poly(dT) and poly[d(A-T)].cntdot.poly[d(A-T)]. Biochemistry.

[34] McGhee J.D., von Hippel P.H. (1974). Theoretical aspects of DNA-protein interactions: Co-operative and non-co-operative binding of large ligands to a one-dimensional homogeneous lattice. J. Mol. Biol..

[35] Scatchard G. (2006). The Attractions of Proteins for Small Molecules and Ions. Ann. N. Y. Acad. Sci..

[36] Mergny J.-L., Lacroix L. (2003). Analysis of Thermal Melting Curves. Oligonucleotides.

[37] Cantor C.R., Schimmel P.R. (1980). Biophysical Chemistry.

[38] Arya D.P., Coffee R.L., Willis B., Abramovitch A.I. (2001). Aminoglycoside–Nucleic Acid Interactions:  Remarkable Stabilization of DNA and RNA Triple Helices by Neomycin. J. Am. Chem. Soc..

[39] Arya D.P. (2010). New Approaches Toward Recognition of Nucleic Acid Triple Helices. Acc. Chem. Res..

[40] Zonjić I., Kurutos A., Mihovilović P., Crnolatac I., Tumir L.-M., Paić A.T., Kralj J., Horvat L., Brozovic A., Stojković R. (2022). Formation of triplex rA/dA-containing nucleic acid helices induced by new thiazole orange analogues and their biological evaluation. H-aggregate formation conditioned by rA sequence. Dye. Pigment..

[41] Pilch D.S., Kirolos M.A., Breslauer K.J. (2002). Berenil binding to higher ordered nucleic acid structures: Complexation with a DNA and RNA triple helix. Biochemistry.

[42] Sinha R., Kumar G.S. (2009). Interaction of Isoquinoline Alkaloids with an RNA Triplex: Structural and Thermodynamic Studies of Berberine, Palmatine, and Coralyne Binding to Poly(U).Poly(A)*Poly(U). J. Phys. Chem. B.

[43] Dalla Pozza M., Abdullrahman A., Cardin C.J., Gasser G., Hall J.P. (2022). Three’s a crowd—Stabilisation, structure, and applications of DNA triplexes. Chem. Sci..

[44] Jain A., Wang G., Vasquez K.M. (2008). DNA triple helices: Biological consequences and therapeutic potential. Biochimie.

[45] Watson J.D., Baker T.A., Bell S.P., Gann A.A.F., Levine M., Losick R.M. (2013). Molecular Biology of the Gene.

[46] Rodger A., Nordén B. (1997). Circular Dichroism and Linear Dichroism.

[47] Šmidlehner T., Piantanida I., Pescitelli G. (2018). Polarization spectroscopy methods in the determination of interactions of small molecules with nucleic acids—Tutorial. Beilstein J. Org. Chem..

[48] Zafar M.N., Perveen F., Nazar M.F., Mughal E.U., Rafique H., Tahir M.N., Akbar M.S., Zahra S. (2017). Synthesis, characterization, DNA-Binding, enzyme inhibition and antioxidant studies of new N -methylated derivatives of pyridinium amine. J. Mol. Struct..

[49] Berova N., Nakanishi K., Woody R.W. (2000). Circular dichroism Principles and Applications.

[50] Eriksson M., Nordén B. (2001). Linear and circular dichroism of drug-nucleic acid complexes. Methods in Enzymology.

[51] Bernardi G. (2000). Isochores and the evolutionary genomics of vertebrates. Gene.

[52] Alvarez-Valin F., Lamolle G., Bernardi G. (2002). Isochores, GC 3 and mutation biases in the human genome. Gene.

[53] Ban Ž., Žinić B., Vianello R., Schmuck C., Piantanida I. (2017). Nucleobase–Guanidiniocarbonyl-Pyrrole Conjugates as Novel Fluorimetric Sensors for Single Stranded RNA. Molecules.

[54] Matić J., Tandarić T., Radić Stojković M., Šupljika F., Karačić Z., Tomašić Paić A., Horvat L., Vianello R., Tumir L.-M. (2023). Phenanthridine–pyrene conjugates as fluorescent probes for DNA/RNA and an inactive mutant of dipeptidyl peptidase enzyme. Beilstein J. Org. Chem..

[55] Chaires J.B., Dattagupta N., Crothers D.M. (2002). Studies on interaction of anthracycline antibiotics and deoxyribonucleic acid: Equilibrium binding studies on the interaction of daunomycin with deoxyribonucleic acid. Biochemistry.

[56] Bresloff J.L., Crothers D.M. (2002). Equilibrium studies of ethidium-polynucleotide interactions. Biochemistry.

[57] Chalikian T.V., Völker J., Plum G.E., Breslauer K.J. (1999). A more unified picture for the thermodynamics of nucleic acid duplex melting: A characterization by calorimetric and volumetric techniques. Proc. Natl. Acad. Sci. USA.

[58] Mickisch G., Fajta S., Keilhauer G., Schlick E., Tschada R., Alken P. (1990). Chemosensitivity testing of primary human renal cell carcinoma by a tetrazolium based microculture assay (MTT). Urol. Res..

[59] Frisch M.J., Trucks G.W., Schlegel H.B., Scuseria G.E., Robb M.A., Cheeseman J.R., Scalmani G., Barone V., Petersson G.A., Nakatsuji H. (2016). Gaussian 16, Revision C.01.

[60] Bauer P., Hess B., Lindahl E. (2023). GROMACS 2022.5 Manual (2022.5).

[61] Darden T., York D., Pedersen L. (1993). Particle mesh Ewald: An N⋅log(N) method for Ewald sums in large systems. J. Chem. Phys..

[62] Jeddi I., Saiz L. (2017). Three-dimensional modeling of single stranded DNA hairpins for aptamer-based biosensors. Sci. Rep..

[63] Beč A., Vianello R., Hranjec M. (2023). Synthesis and spectroscopic characterization of multifunctional D-π—A benzimidazole derivatives as potential pH sensors. J. Mol. Liq..

[64] Boček I., Hranjec M., Vianello R. (2022). Imidazo[4,5-b]pyridine derived iminocoumarins as potential pH probes: Synthesis, spectroscopic and computational studies of their protonation equilibria. J. Mol. Liq..

