# Supplementary material for: Distinctive Nucleic Acid Recognition by Lysine-Embedded Phenanthridine Peptides
Source: Int J Mol Sci. 2024 Apr 29;25(9):4866. doi: 10.3390/ijms25094866 (PMC11084427; doi:10.3390/ijms25094866)
Supplement: Supplementary file 1 [file ijms-25-04866-s001.zip › ijms-2963137-supplementary.pdf]

# Supporting Information

## Distinctive Nucleic Acid Recognition by Lysine-Embedded Phenanthridine Peptides

Josipa Matić [1], Patryciusz Piotrowski [2], Lucija Vrban [3], Renata Kobetić [1], Robert Vianello [3], Ivona Jurić [1], Ivana Fabijanić [1], Margareta Pernar Kovač [4], Anamaria Brozovic [4], Ivo Piantanida [1], Carsten Schmuck [2,†] and Marijana Radić Stojković [1,*]

1 Laboratory for Biomolecular Interactions and Spectroscopy, Division of Organic Chemistry and Biochemistry, Ruđer Bošković Institute, Bijenička cesta 54, 10000 Zagreb, Croatia; josipa.matic@gmail.com (J.M.); renata.kobetic@irb.hr (R.K.); juric.ivona@student.pmf.hr (I.J.); i.s.fabijanic@gmail.com (I.F.); ivo.piantanida@irb.hr (I.P.)

2 Institute for Organic Chemistry, University of Duisburg-Essen, Universitätsstrasse 7, 45141 Essen, Germany; p.piotrowski@gmx.net (P.P.); carsten.schmuck@uni-due.de (C.S.)

3 Laboratory for the Computational Design and Synthesis of Functional Materials, Division of Organic Chemistry and Biochemistry, Ruđer Bošković Institute, Bijenička cesta 54, 10000 Zagreb, Croatia; lvrban@irb.hr (L.V.); robert.vianello@irb.hr (R.V.)

4 Division of Molecular Biology, Ruđer Bošković Institute, Bijenička cesta 54, 10000 Zagreb, Croatia; margareta.pernar@irb.hr (M.P.K.); anamaria.brozovic@irb.hr (A.B.)

* Correspondence: mradic@irb.hr; Tel.: +385-1-4571220; Fax: +385-1-4680195

† Deceased 1 August 2019.

## Contents

1. Spectroscopic characterization of phenanthridine peptides in aqueous solutions.

2. Interactions of phenanthridine peptides with polynucleotides in neutral and weakly acid medium (pH 7.0 and pH 5.0)

2.1. Fluorimetric titrations

2.2. Thermal melting experiments

2.3. Circular dichroism (CD) titrations

2.4. Mass spectra

3. Computational analysis of **19**, **22** and **23**

# 1. Spectroscopic characterization of phenanthridine peptides in aqueous solutions

*1.1. UV/Vis spectra at pH 7.0*

**19**

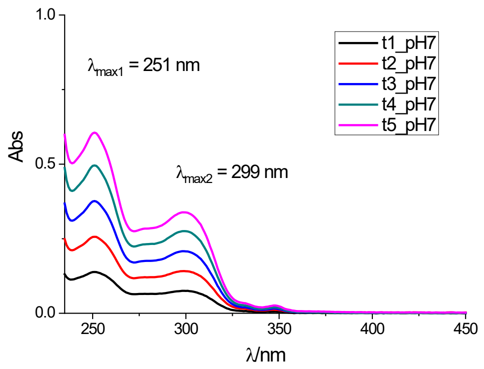


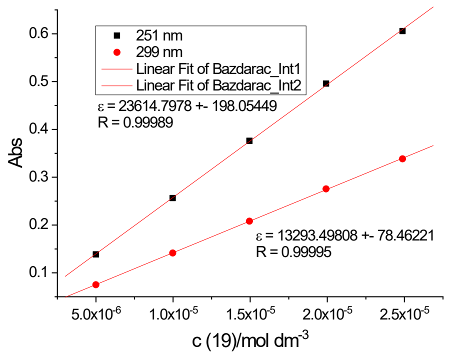


**22**

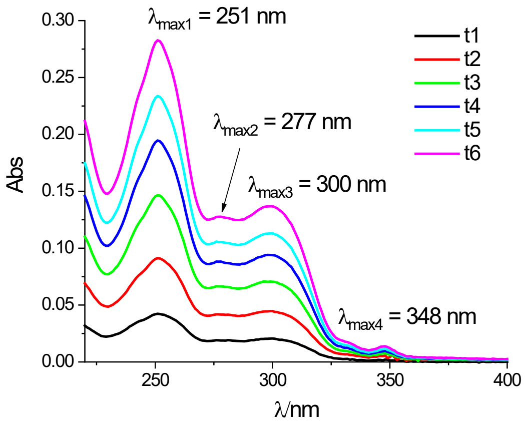


concentration range from 1 × $10^{-6}$- 6 × $10^{-6}$ mol $dm^{-3}$

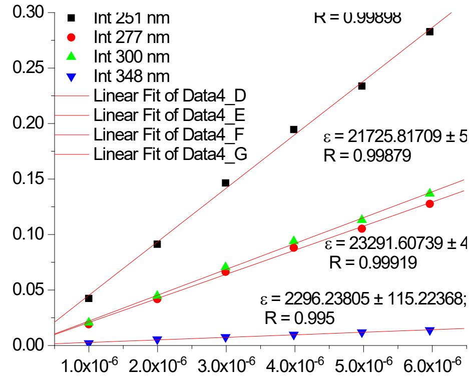


**22**

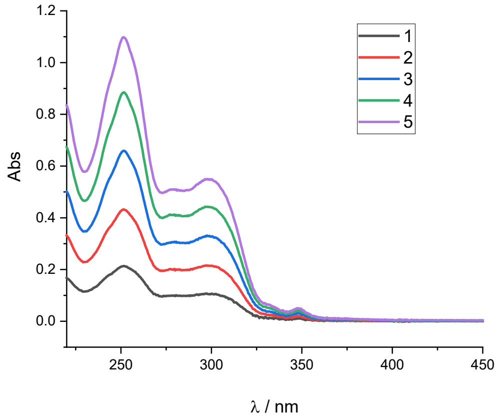


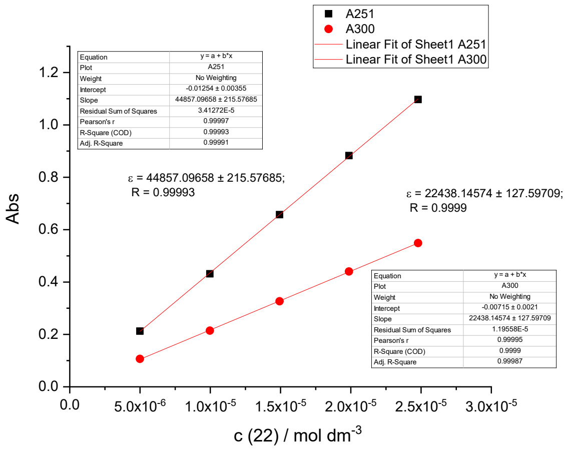

**23**

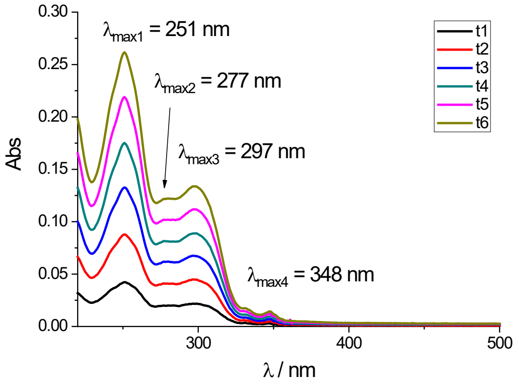


concentration range from 1 × 10$^{-6}$- 6 × 10$^{-6}$ mol dm$^{-3}$

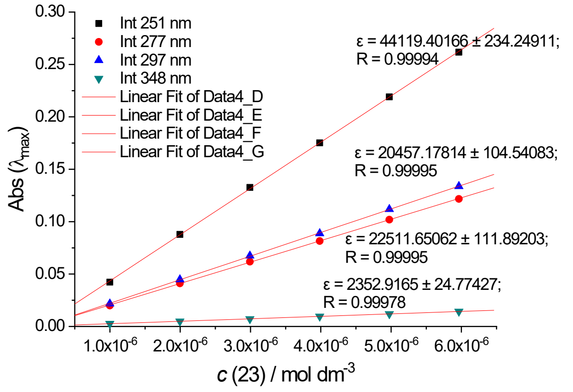


**23**

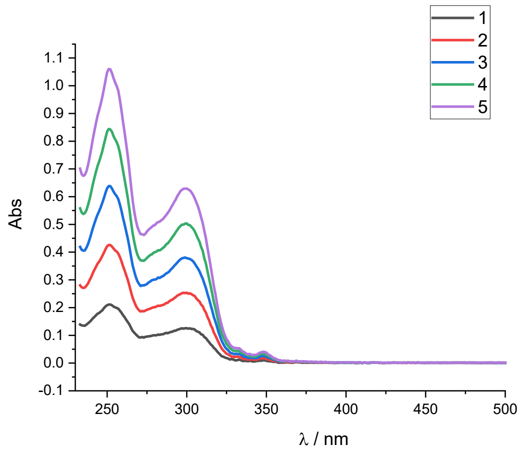


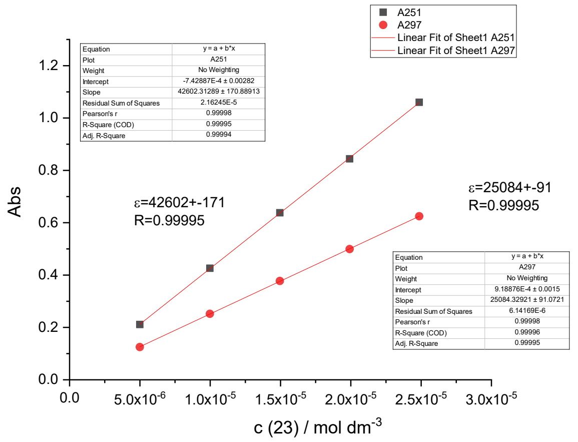


Figure S1. UV/Vis spectra changes of **19, 22, 23** at different concentrations (concentration range from 5 × 10$^{-6}$- 2.5 × 10$^{-5}$ mol dm$^{-3}$ ) at pH 7.0, sodium cacodylate buffer, *I*=0,05 M.

*1.2. UV/Vis spectra at pH 5.0*

**19**

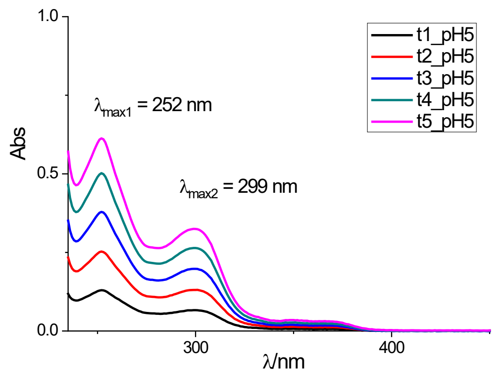


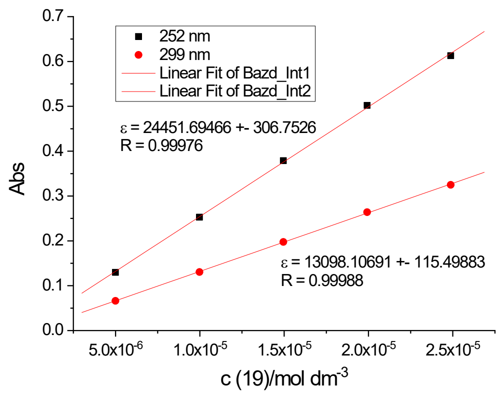

| | | |
|---|---|---|
| **22** | 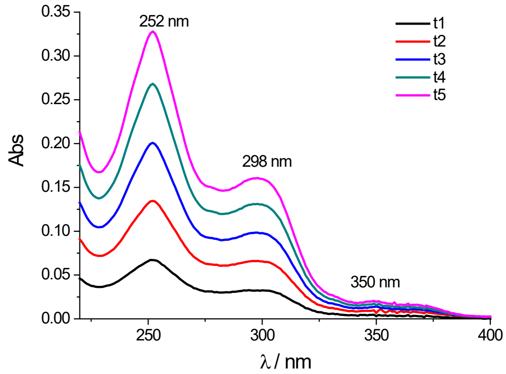<br>concentration range from 1 × $10^{-6}$- 6 × $10^{-6}$ mol $dm^{-3}$ | 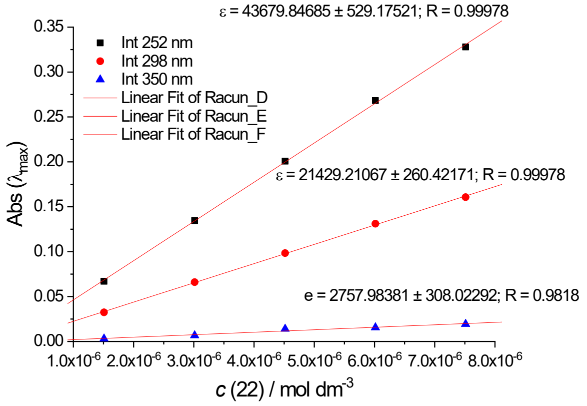 |
| **22** | 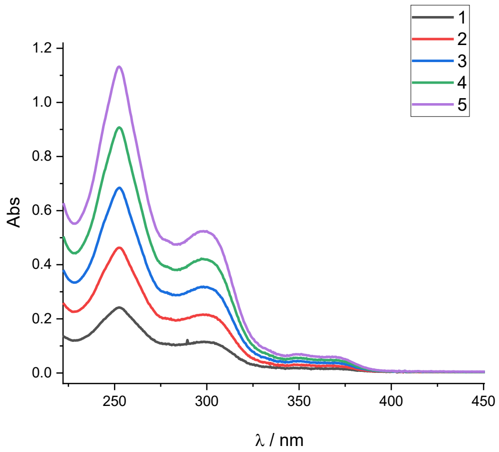 | 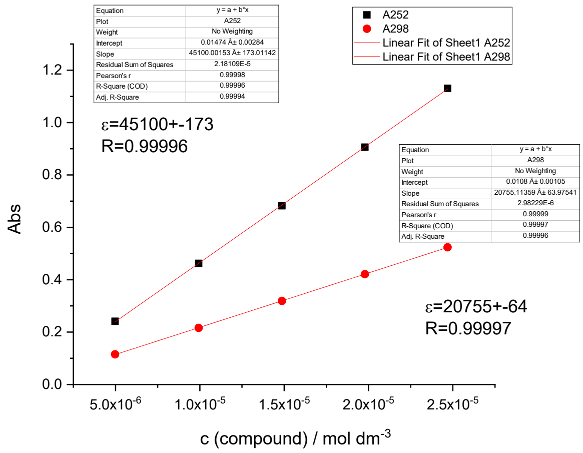 |
| **23** | 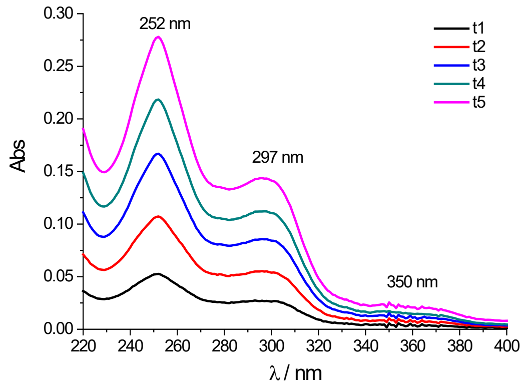<br>concentration range from 1 × $10^{-6}$- 6 × $10^{-6}$ mol $dm^{-3}$ | 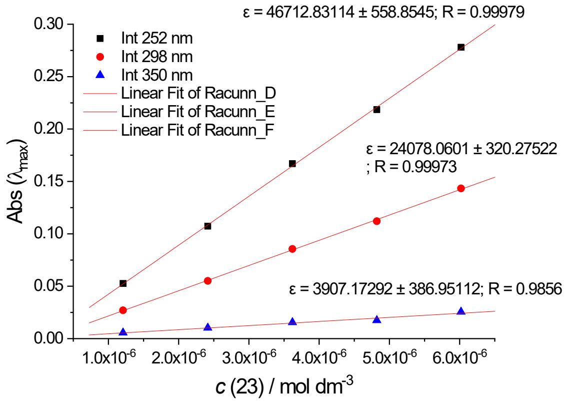 |

**23**

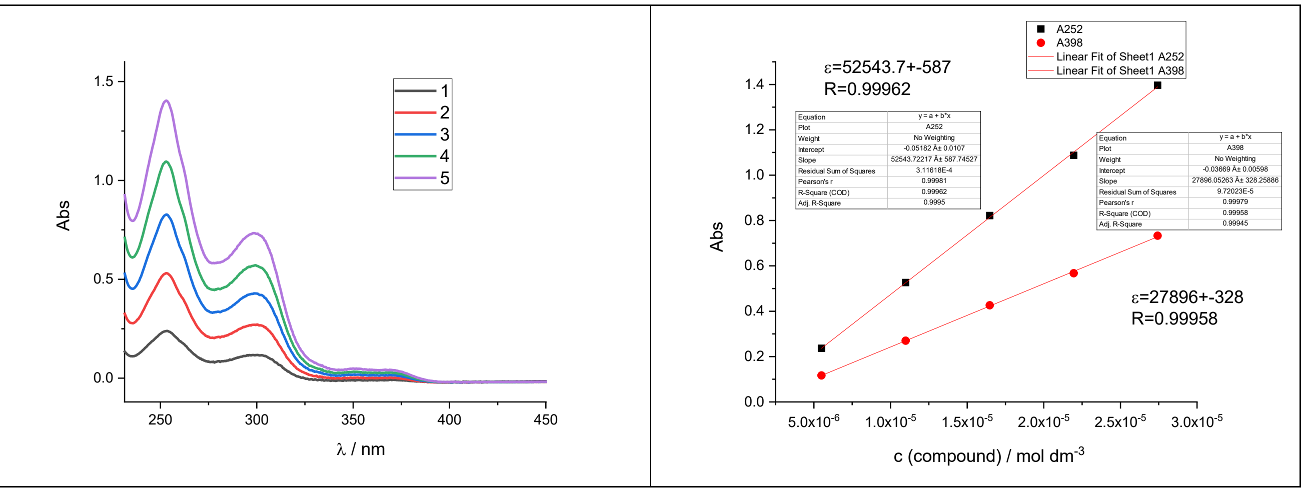


Figure S2. UV/Vis spectra changes of **19,22,23** at different concentrations (concentration range from 5 × 10$^{-6}$- 2.5 × 10$^{-5}$ mol dm$^{-3}$) at pH 5.0, sodium cacodylate buffer, *I*=0,05 M.

**A**

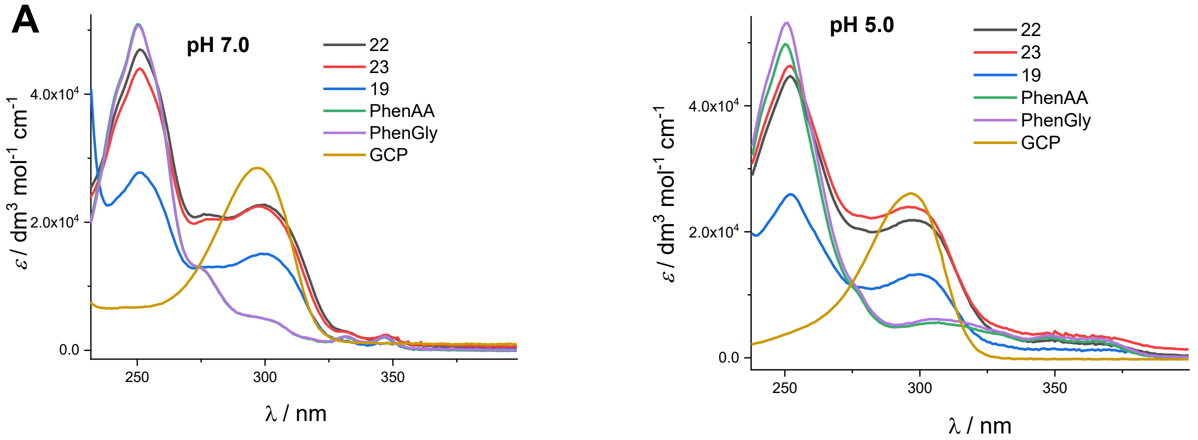


**B**

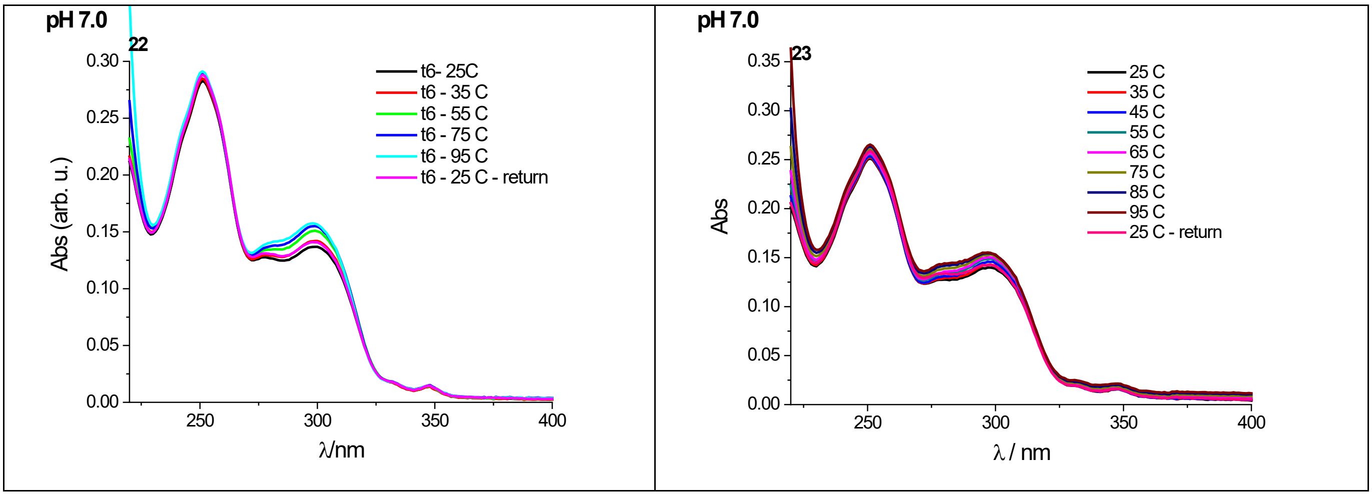

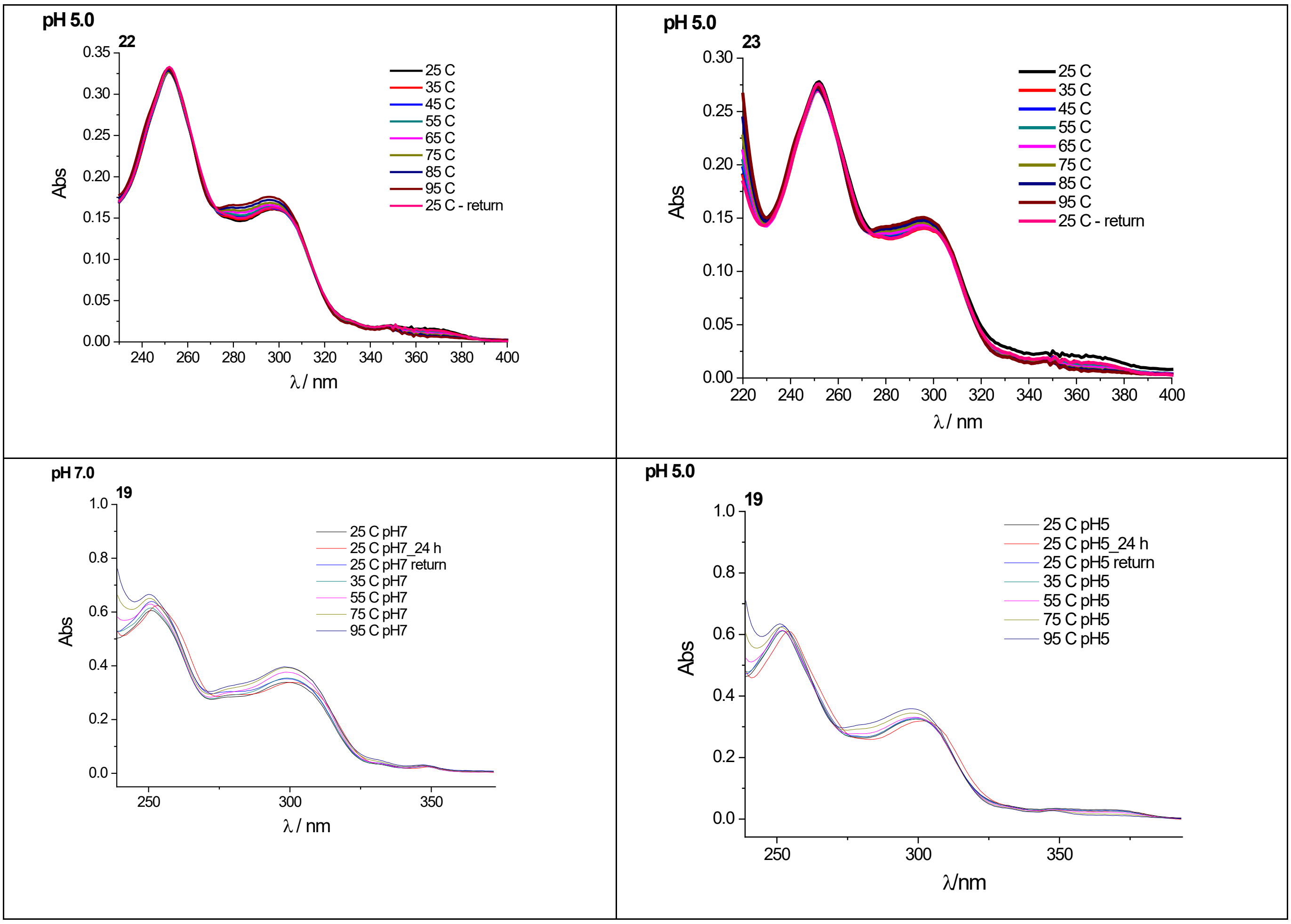


Figure S3. A) Overlapped UV spectra of **22, 23** and **19** with the reference compounds **Phen-AA**, **Phen-Gly** and **GCP**; showing hypochromicity of all chromophores; B) changes of UV spectra of **22, 23** and **19** upon temperature increase.

## *1.3. Fluorescence spectra at pH 7.0 and pH 5.0*

| | pH7.0 | pH5.0 |
|---|---|---|
| **19** | 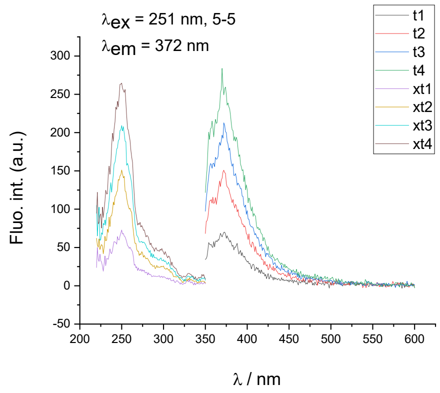  | 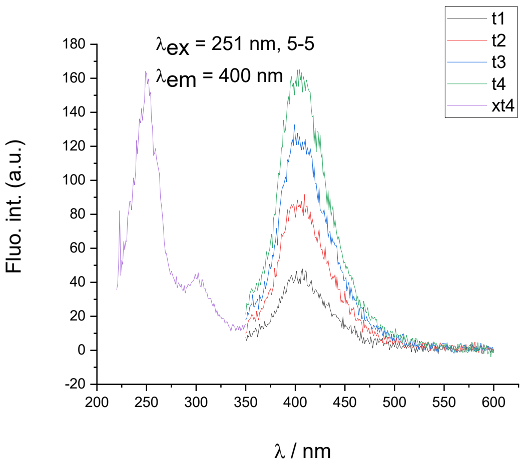  |

**22**

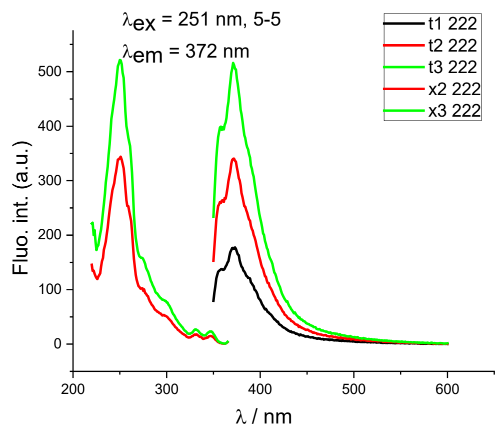


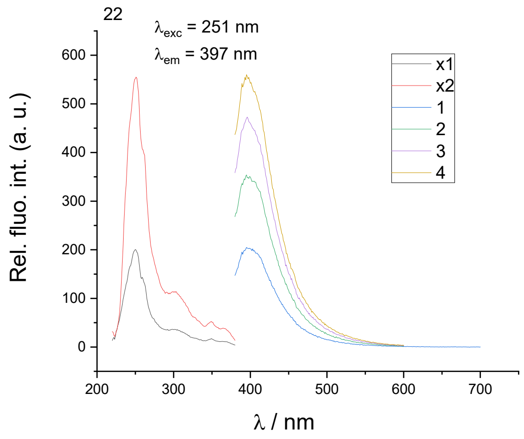


**23**

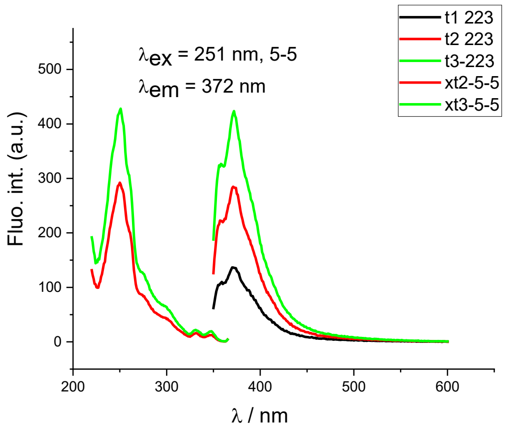


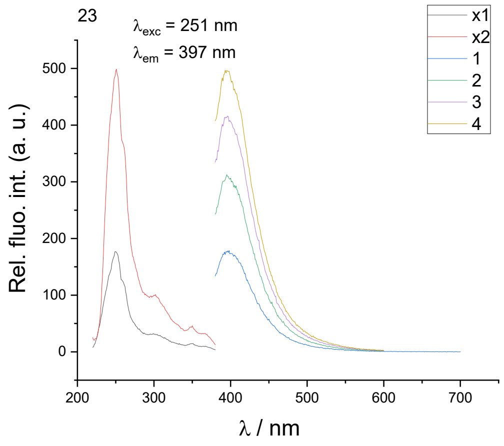


Figure S4. Emission and excitation spectra changes of **19, 22, 23** at different concentrations at $\lambda_{exc}$=251 nm (concentration range from $5 \times 10^{-7}$- $2 \times 10^{-6}$ mol $dm^{-3}$ ) at pH 7.0 and pH 5.0, Na cacodylate buffer, *I*=0.05 mol $dm^{-3}$.

### *1.4. CD spectra of **19, 22** and **23***

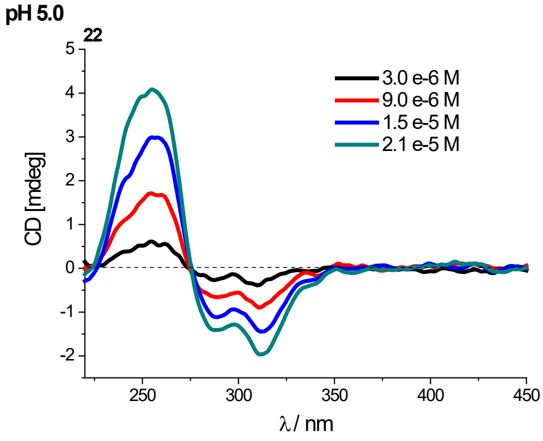


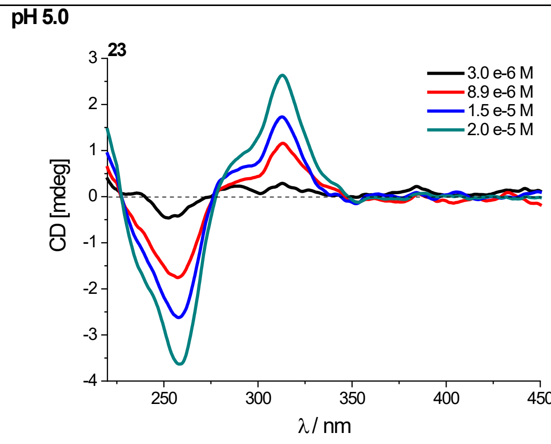

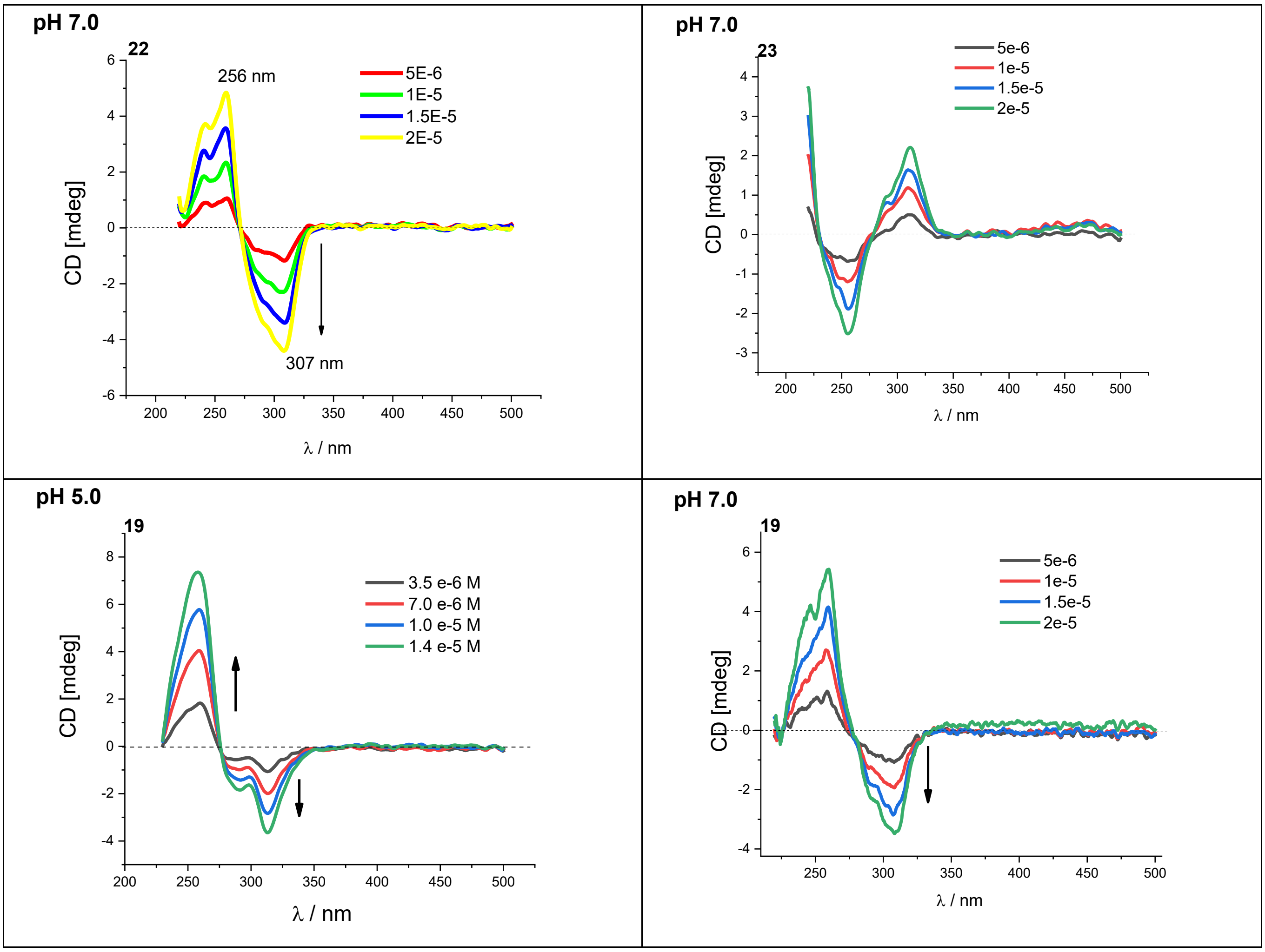


Figure S5. CD spectra changes of **19, 22** and **23** at different concentrations (concentration range from $3.0 \times 10^{-6}$- $6 \times 10^{-5}$ mol dm$^{-3}$) at pH = 7.0 and 5.0 (buffer sodium cacodylate, *I* = 0.05 mol dm$^{-3}$).

## 2. Interactions of phenanthridine peptides with polynucleotides in neutral and weakly acid medium (pH 7.0 and pH 5.0)

### 2.1. Fluorimetric titrations

#### *2.1.1. Fluorimetric titrations at* pH 7.0

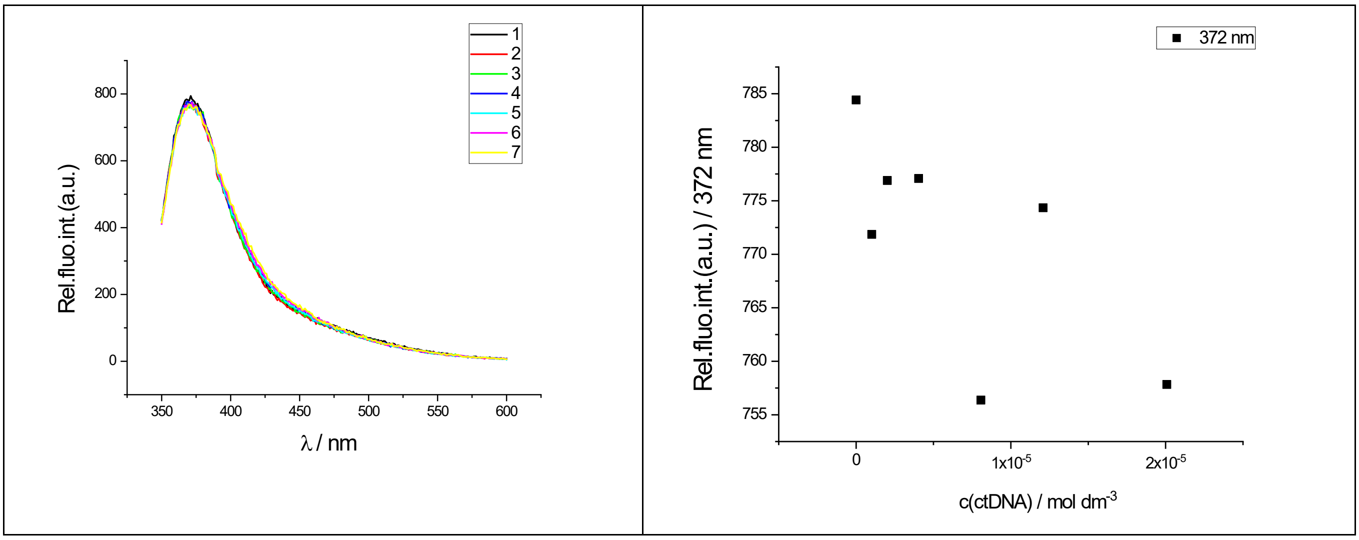


Figure S6. a) Changes in fluorescence spectrum of **19** ($c$= 1.0 × $10^{-6}$ mol $dm^{-3}$, $\lambda_{exc}$=300 nm) upon titration with ctDNA ($c$= 1.0 × $10^{-6}$ - 2 × $10^{-5}$ mol $dm^{-3}$); b) Dependence of **19** absorbance at $\lambda_{max}$ = 372 nm on c(ctDNA), at pH 7.0, sodium cacodylate buffer, $I$ = 0.05 mol $dm^{-3}$.

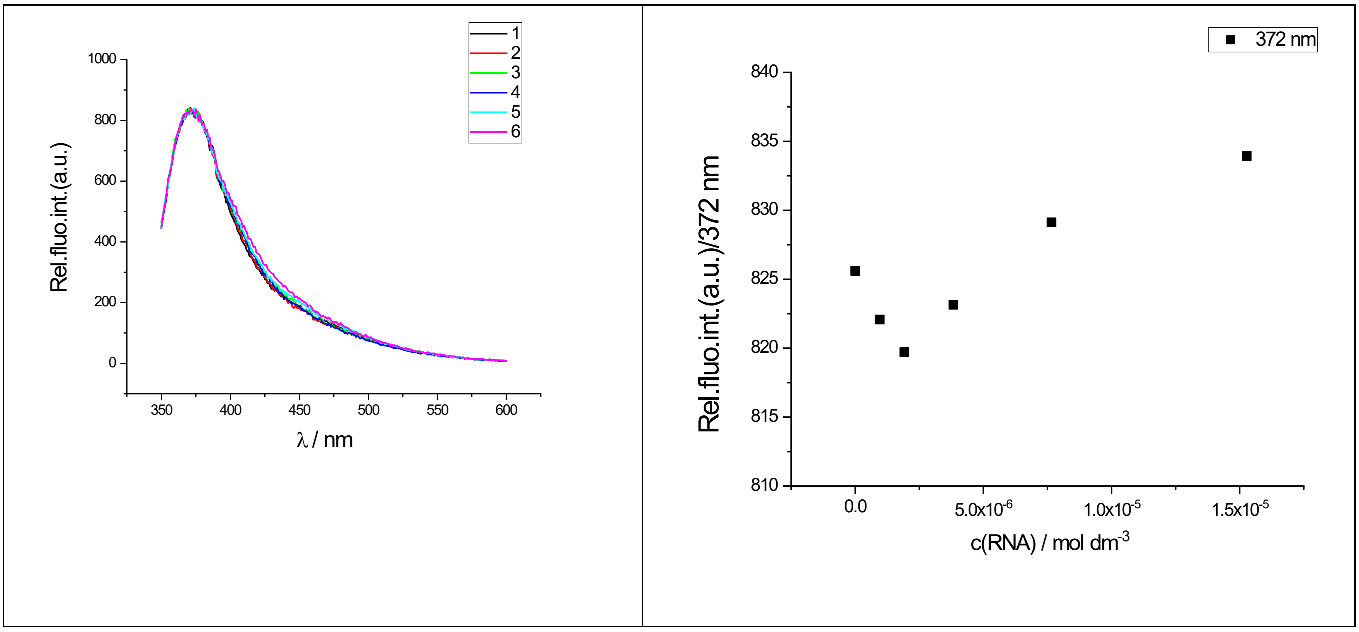


Figure S7. a) Changes in fluorescence spectrum of **19** ($c$= 1.0 × $10^{-6}$ mol $dm^{-3}$, $\lambda_{exc}$=300 nm) upon titration with poly A-poly U ($c$= 1.0 × $10^{-6}$ – 1.9 × $10^{-5}$ mol $dm^{-3}$); b) Dependence of **19** absorbance at $\lambda_{max}$ = 372 nm on c(poly A-poly U), at pH 7.0, sodium cacodylate buffer, $I$ = 0.05 mol $dm^{-3}$.

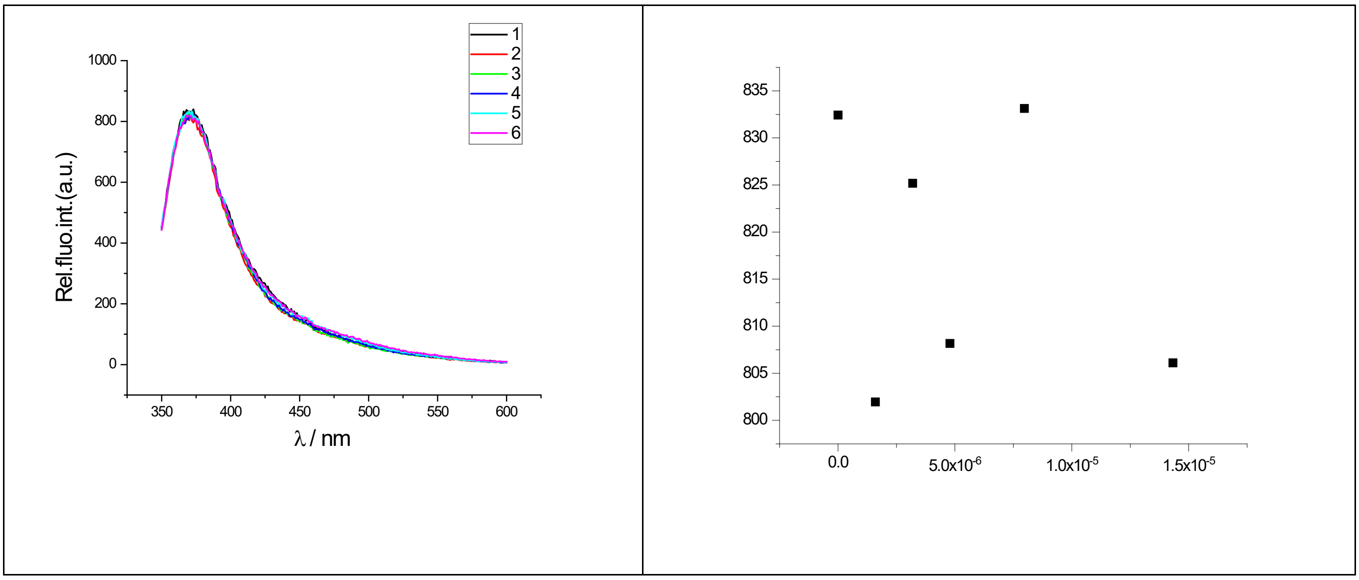


Figure S8. a) Changes in fluorescence spectrum of **19** ($c$= 1.0 × 10$^{-6}$ mol dm$^{-3}$, $\lambda_{exc}$=300 nm) upon titration with poly(dGdC)$_2$ ($c$= 1.6 × 10$^{-6}$ – 1.4 × 10$^{-5}$ mol dm$^{-3}$); b) Dependence of **19** absorbance at $\lambda_{max}$ = 372 nm on c(poly(dGdC)$_2$), at pH 7.0, sodium cacodylate buffer, $I$ = 0.05 mol dm$^{-3}$.

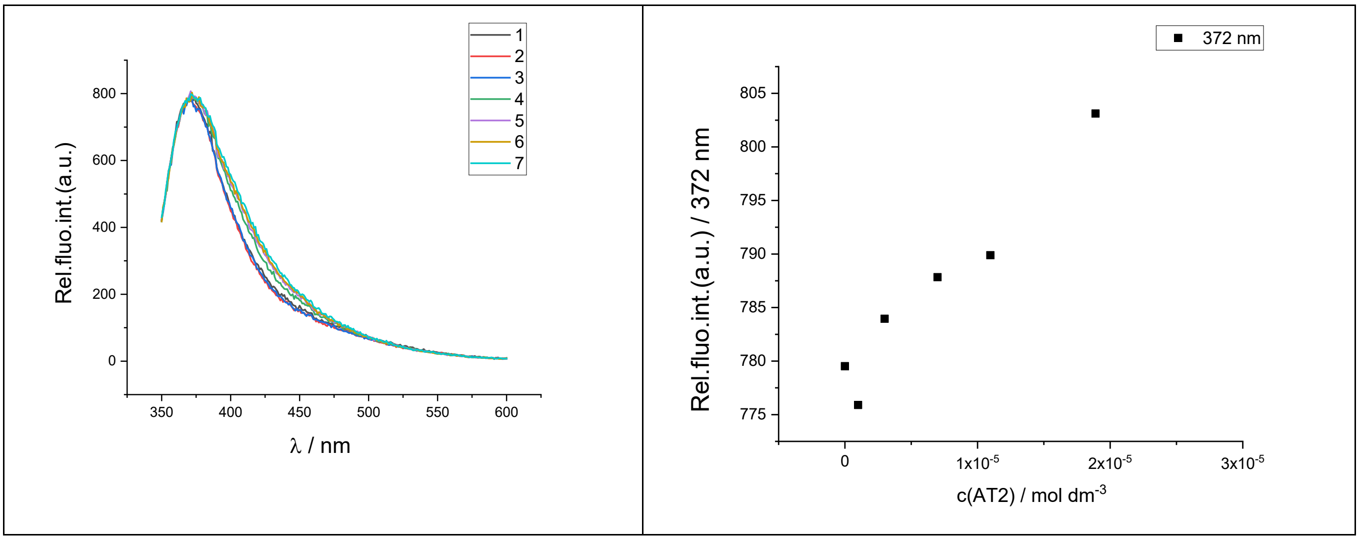


Figure S9. a) Changes in fluorescence spectrum of **19** ($c$= 1.0 × 10$^{-6}$ mol dm$^{-3}$, $\lambda_{exc}$=300 nm) upon titration with poly(dAdT)$_2$ ($c$= 1.0 × 10$^{-6}$ – 1.9 × 10$^{-5}$ mol dm$^{-3}$); b) Dependence of **19** absorbance at $\lambda_{max}$ = 372 nm on c(poly(dAdT)$_2$), at pH 7.0, sodium cacodylate buffer, $I$ = 0.05 mol dm$^{-3}$.

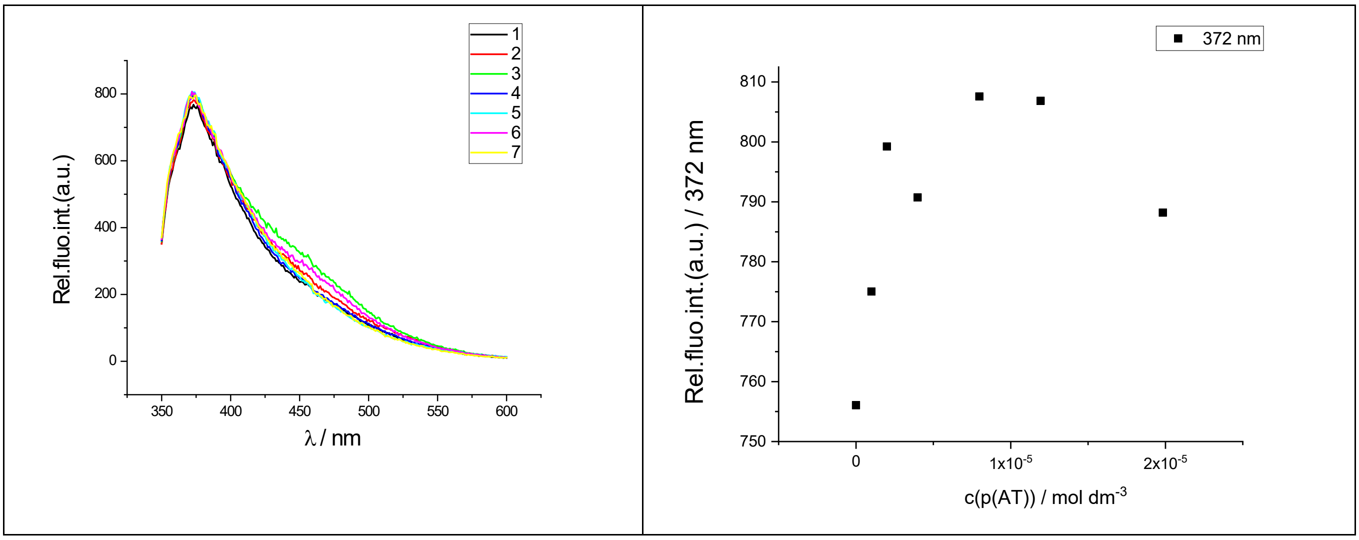


Figure S10. a) Changes in fluorescence spectrum of **19** ($c$= 1.0 × $10^{-6}$ mol $dm^{-3}$, $\lambda_{exc}$=300 nm) upon titration with poly dA-poly dT ($c$= 1.0 × $10^{-6}$ – 2.0 × $10^{-5}$ mol $dm^{-3}$); b) Dependence of **19** absorbance at $\lambda_{max}$ = 372 nm on c(poly dA-poly dT), at pH 7.0, sodium cacodylate buffer, $I$ = 0.05 mol $dm^{-3}$.

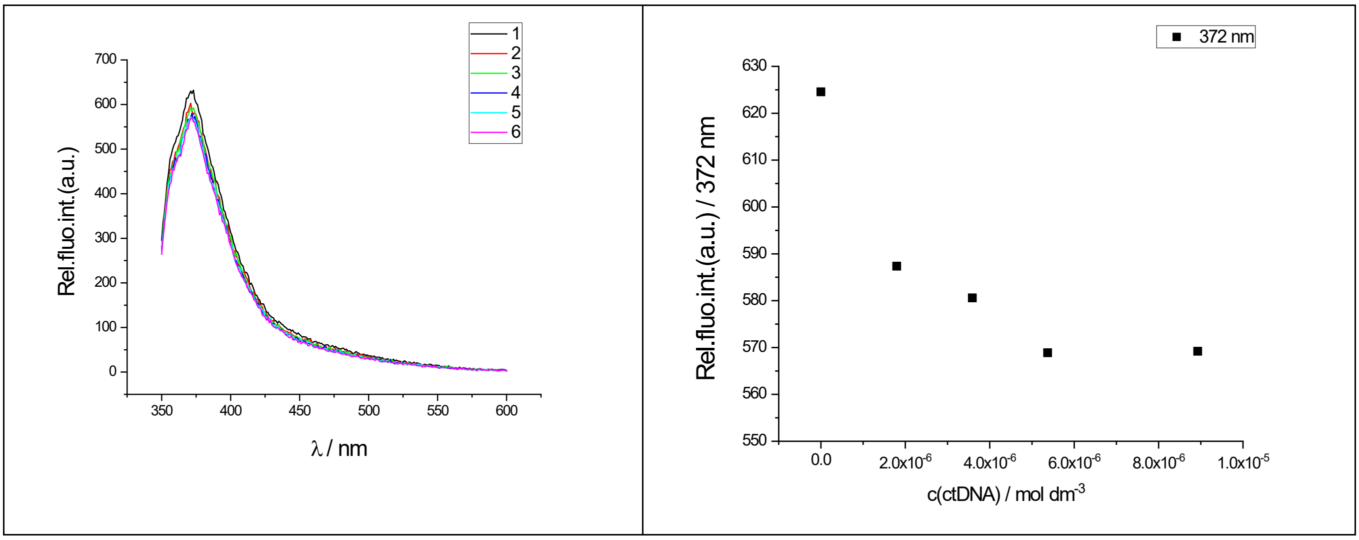


Figure S11. a) Changes in fluorescence spectrum of **22** ($c$= 1.0 × $10^{-6}$ mol $dm^{-3}$, $\lambda_{exc}$=300 nm) upon titration with ctDNA ($c$= 9.0 × $10^{-7}$ - 9 × $10^{-6}$ mol $dm^{-3}$); b) Dependence of **22** absorbance at $\lambda_{max}$ = 372 nm on c(ctDNA), at pH 7.0, sodium cacodylate buffer, $I$ = 0.05 mol $dm^{-3}$.

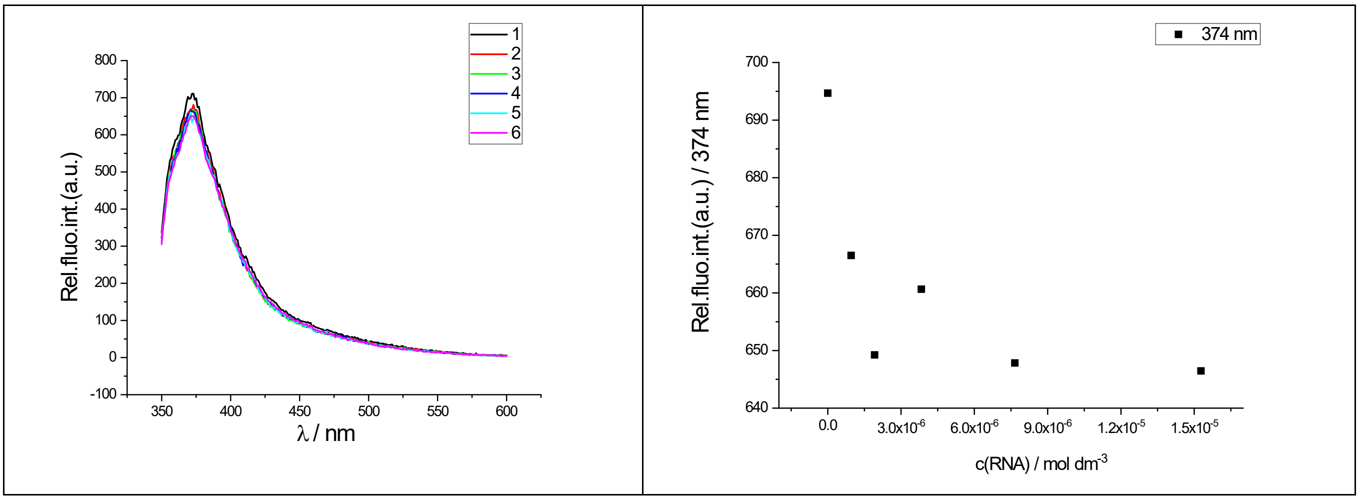


Figure S12. a) Changes in fluorescence spectrum of **22** ($c$= 1.0 × $10^{-6}$ mol $dm^{-3}$, $\lambda_{exc}$=300 nm) upon titration with poly A-poly U ($c$= 1.0 × $10^{-6}$ – 1.5 × $10^{-5}$ mol $dm^{-3}$); b) Dependence of **22** absorbance at $\lambda_{max}$ = 374 nm on c(poly A-poly U), at pH 7.0, sodium cacodylate buffer, $I$ = 0.05 mol $dm^{-3}$.

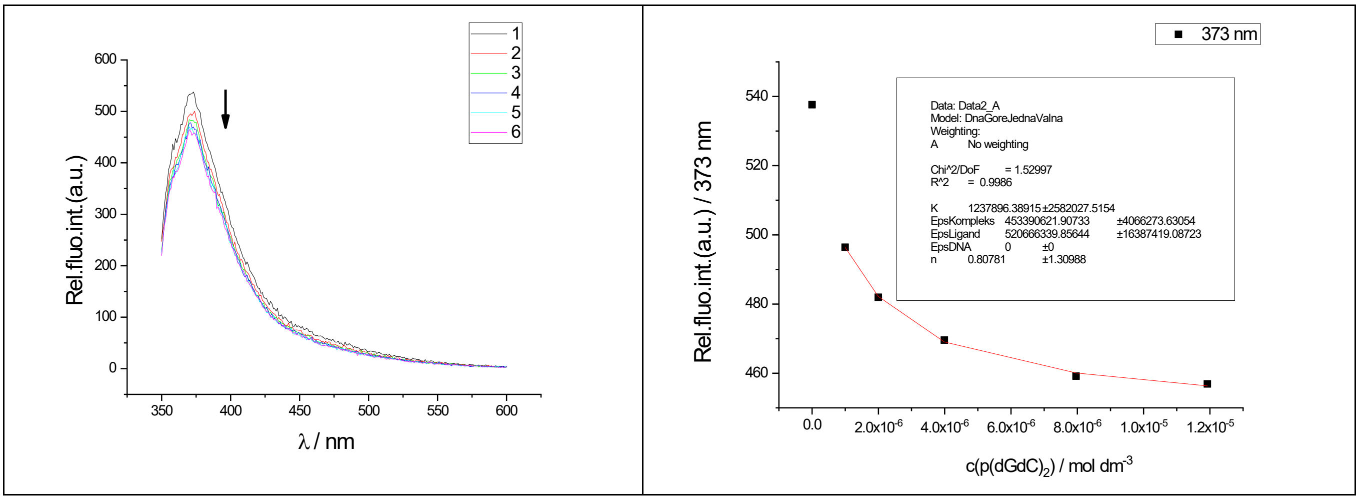


Figure S13. a) Changes in fluorescence spectrum of **22** ($c$= 1.0 × $10^{-6}$ mol $dm^{-3}$, $\lambda_{exc}$=300 nm) upon titration with poly(dGdC)$_2$ ($c$= 1.0 × $10^{-6}$ – 1.2 × $10^{-5}$ mol $dm^{-3}$); b) Dependence of **22** absorbance at $\lambda_{max}$ = 373 nm on c(poly(dGdC)$_2$), at pH 7.0, sodium cacodylate buffer, $I$ = 0.05 mol $dm^{-3}$.

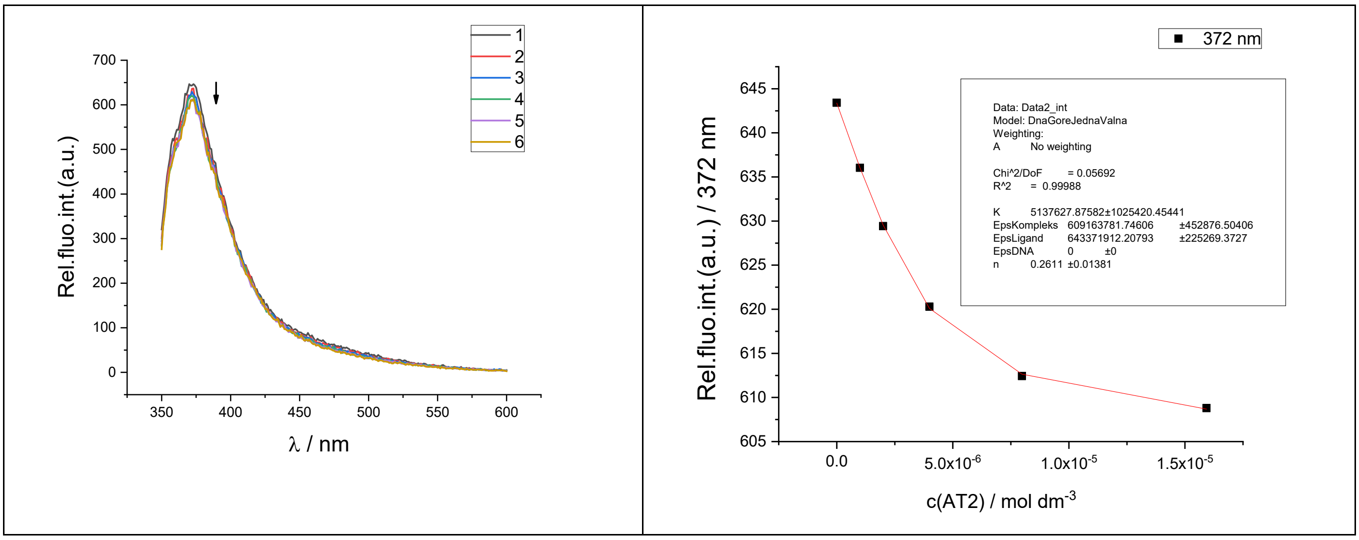

Figure S14. a) Changes in fluorescence spectrum of **22** ($c$= 1.0 × $10^{-6}$ mol $dm^{-3}$, $\lambda_{exc}$=300 nm) upon titration with poly(dAdT)$_2$ ($c$= 1.0 × $10^{-6}$ – 1.6 × $10^{-5}$ mol $dm^{-3}$); b) Dependence of **22** absorbance at $\lambda_{max}$ = 372 nm on c(poly(dAdT)$_2$), at pH 7.0, sodium cacodylate buffer, $I$ = 0.05 mol $dm^{-3}$.

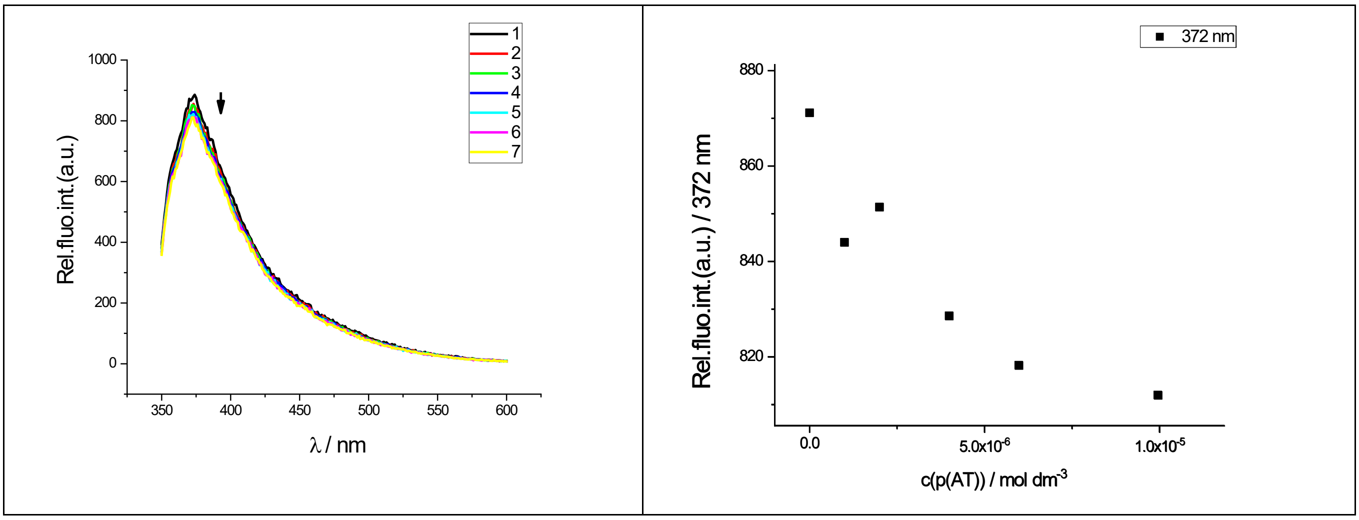


Figure S15. a) Changes in fluorescence spectrum of **22** ($c$= 1.0 × $10^{-6}$ mol $dm^{-3}$, $\lambda_{exc}$=300 nm) upon titration with poly dA-poly dT ($c$= 1.0 × $10^{-6}$ – 1.8 × $10^{-5}$ mol $dm^{-3}$); b) Dependence of **22** absorbance at $\lambda_{max}$ = 372 nm on c(poly dA-poly dT), at pH 7.0, sodium cacodylate buffer, $I$ = 0.05 mol $dm^{-3}$.

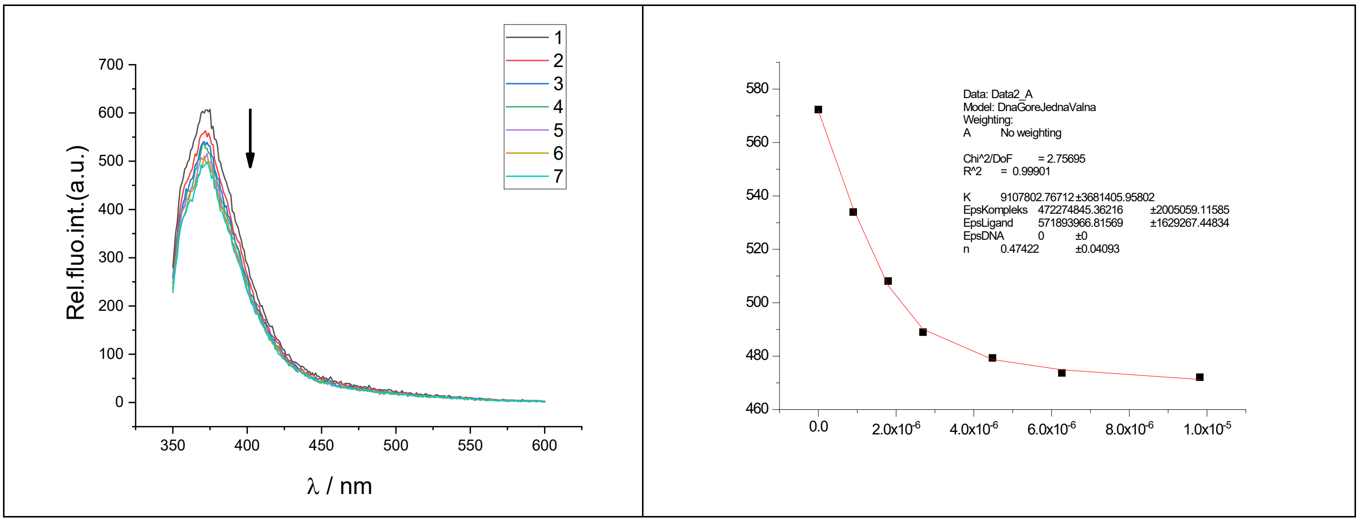


Figure S16. a) Changes in fluorescence spectrum of **23** ($c$= 1.0 × $10^{-6}$ mol $dm^{-3}$, $\lambda_{exc}$=300 nm) upon titration with ctDNA ($c$= 1.0 × $10^{-6}$ – 9.82 × $10^{-6}$ mol $dm^{-3}$); b) Dependence of **23** absorbance at $\lambda_{max}$ = 377 nm on c(ctDNA), at pH 7.0, sodium cacodylate buffer, $I$ = 0.05 mol $dm^{-3}$.

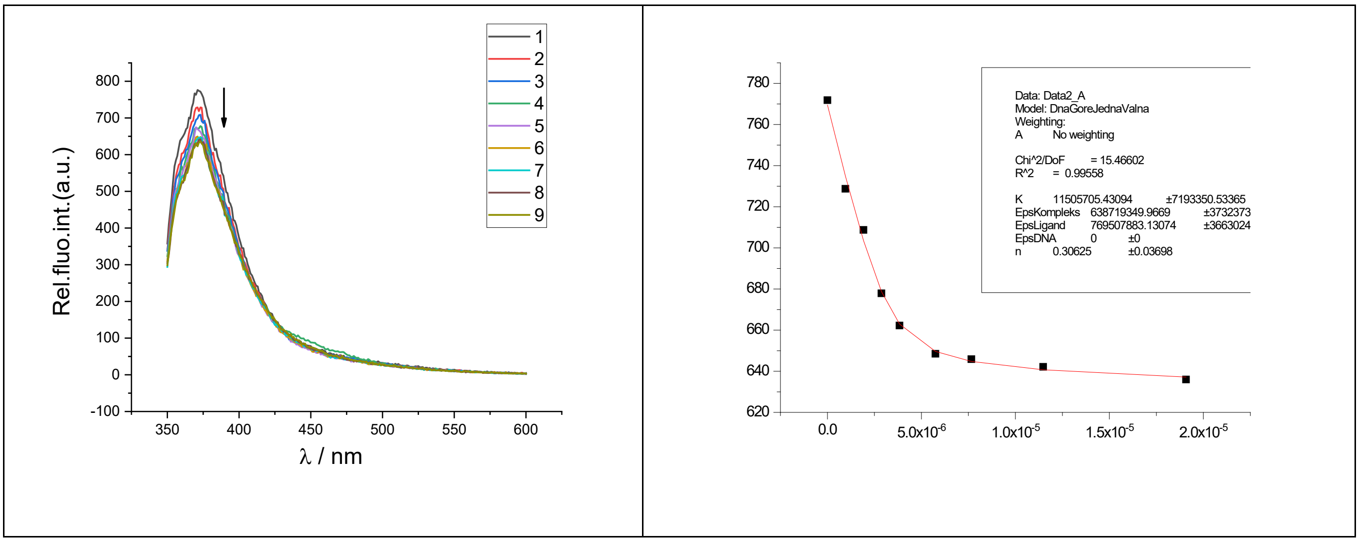


Figure S17. a) Changes in fluorescence spectrum of **23** ($c$= 1.0 × $10^{-6}$ mol $dm^{-3}$, $\lambda_{exc}$=300 nm) upon titration with poly A-poly U ($c$= 1.0 × $10^{-6}$ – 1.9 × $10^{-5}$ mol $dm^{-3}$); b) Dependence of **23** absorbance at $\lambda_{max}$ = 373 nm on c(poly A-poly U), at pH 7.0, sodium cacodylate buffer, $I$ = 0.05 mol $dm^{-3}$.

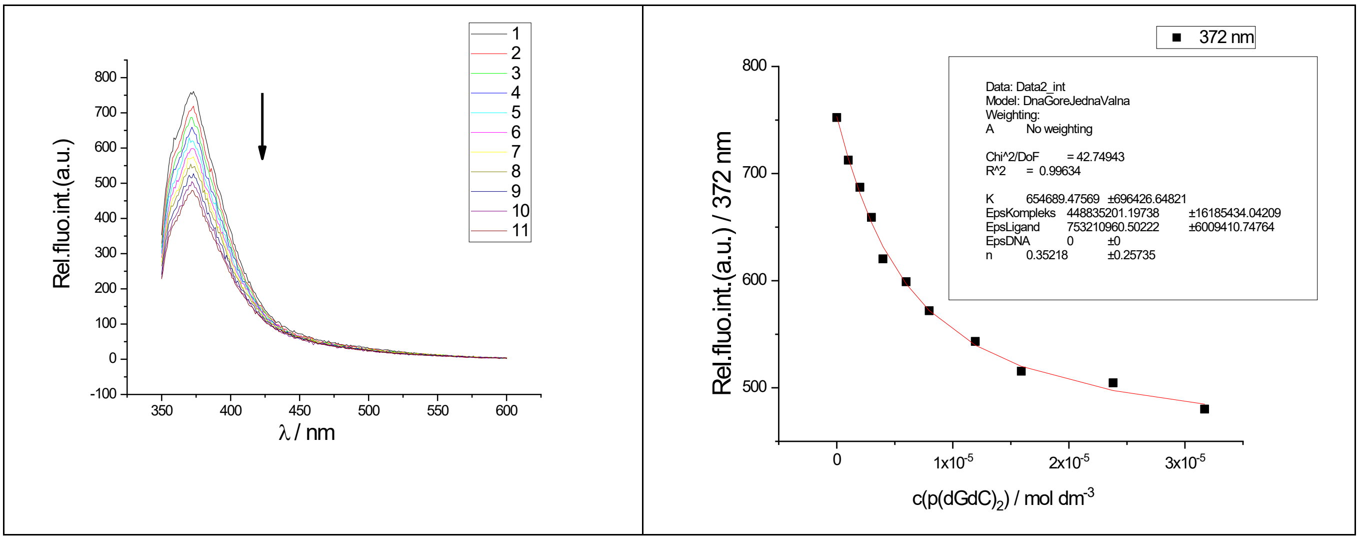


Figure S18. a) Changes in fluorescence spectrum of **23** ($c$= 1.0 × $10^{-6}$ mol $dm^{-3}$, $\lambda_{exc}$=300 nm) upon titration with poly(dGdC)$_2$ ($c$= 1.0 × $10^{-6}$ – 3.1 × $10^{-5}$ mol $dm^{-3}$); b) Dependence of **23** absorbance at $\lambda_{max}$ = 372 nm on c(poly(dGdC)$_2$), at pH 7.0, sodium cacodylate buffer, $I$ = 0.05 mol $dm^{-3}$.

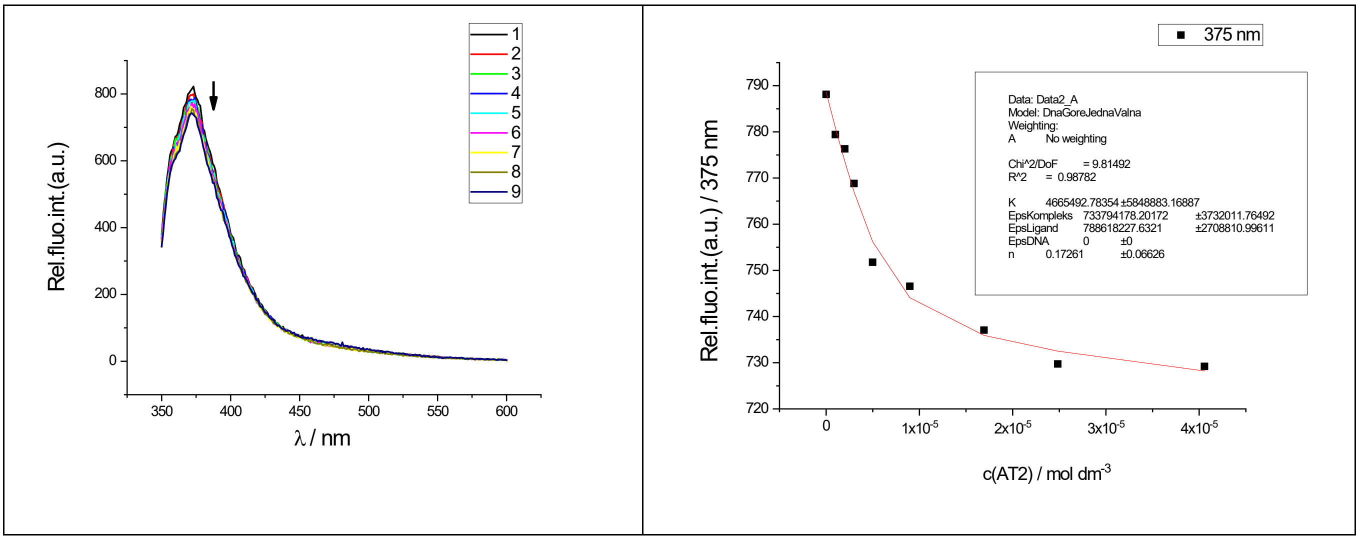


Figure S19. a) Changes in fluorescence spectrum of **23** ($c$= 1.0 × $10^{-6}$ mol $dm^{-3}$, $\lambda_{exc}$=300 nm) upon titration with poly$(dAdT)_2$ ($c$= 1.0 × $10^{-6}$ – 4.1 × $10^{-5}$ mol $dm^{-3}$); b) Dependence of **23** absorbance at $\lambda_{max}$ = 375 nm on c(poly$(dAdT)_2$), at pH 7.0, sodium cacodylate buffer, $I$ = 0.05 mol $dm^{-3}$.

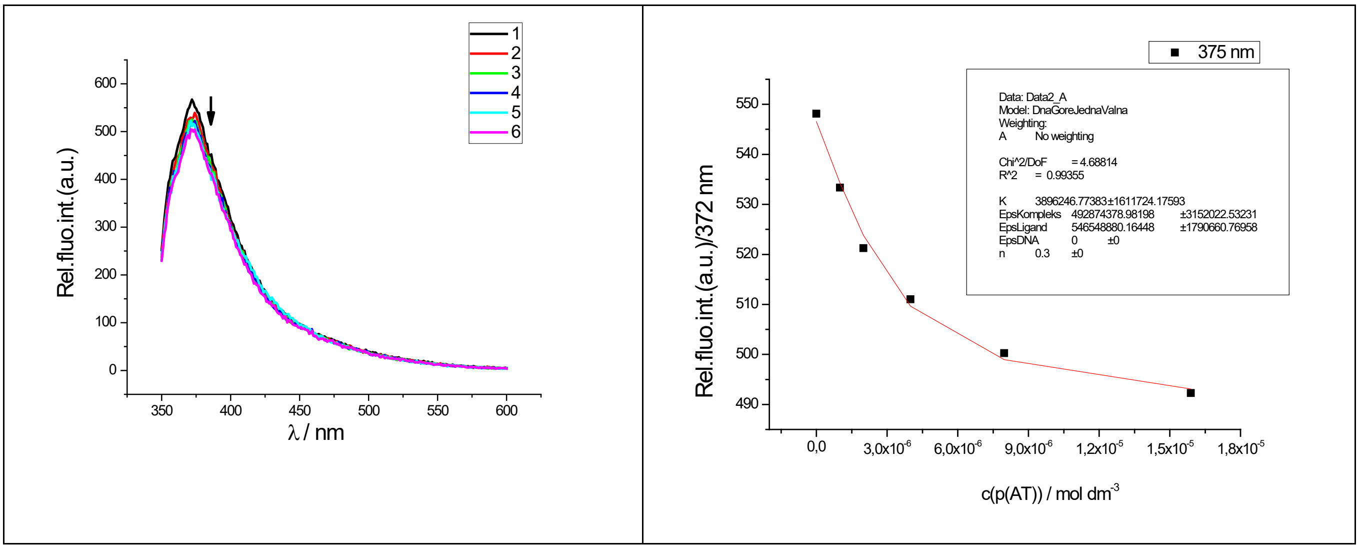


Figure S20. a) Changes in fluorescence spectrum of **23** ($c$= 1.0 × $10^{-6}$ mol $dm^{-3}$, $\lambda_{exc}$=300 nm) upon titration with poly dA-poly dT ($c$= 1.0 × $10^{-6}$ – 1.6 × $10^{-5}$ mol $dm^{-3}$); b) Dependence of **23** absorbance at $\lambda_{max}$ = 375 nm on c(poly dA-poly dT), at pH 7.0, sodium cacodylate buffer, $I$ = 0.05 mol $dm^{-3}$.

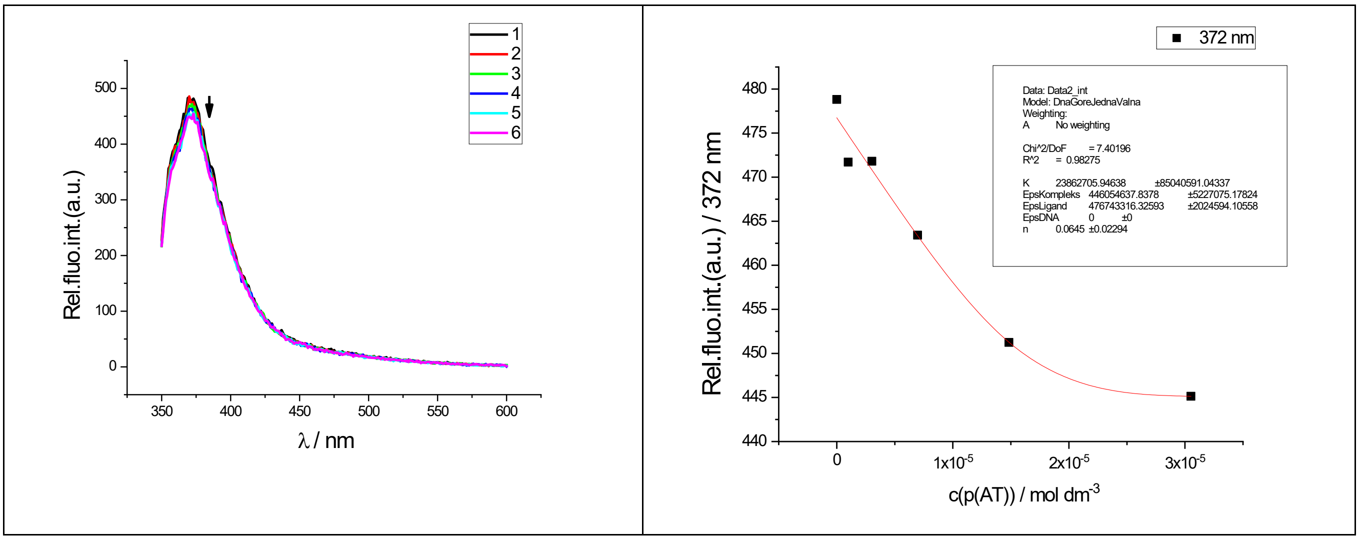


Figure S21. a) Changes in fluorescence spectrum of **23** (*c*= 1.0 × $10^{-6}$ mol $dm^{-3}$, $\lambda_{exc}$=300 nm) upon titration with ATT (*c*= 1.0 × $10^{-6}$ – 1.6 × $10^{-5}$ mol $dm^{-3}$); b) Dependence of **23** absorbance at $\lambda_{max}$ = 372 nm on c(ATT), at pH 7.0, sodium cacodylate buffer, *I* = 0.05 mol $dm^{-3}$.

### *2.1.2. Fluorimetric titrations at pH 5.0*

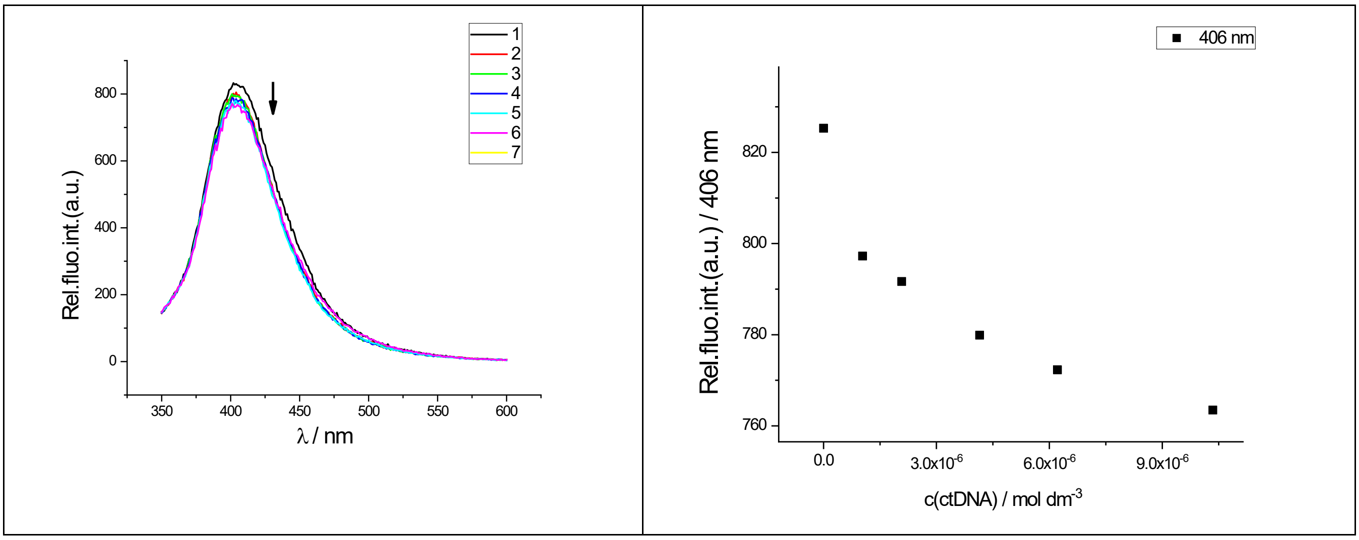


Figure S22. a) Changes in fluorescence spectrum of **19** (*c*= 1.0 × $10^{-6}$ mol $dm^{-3}$, $\lambda_{exc}$=300 nm) upon titration with ctDNA (*c*= 1.0 × $10^{-6}$ - 1 × $10^{-5}$ mol $dm^{-3}$); b) Dependence of **19** absorbance at $\lambda_{max}$ = 406 nm on c(ctDNA), at pH 5.0, sodium cacodylate buffer, *I* = 0.05 mol $dm^{-3}$.

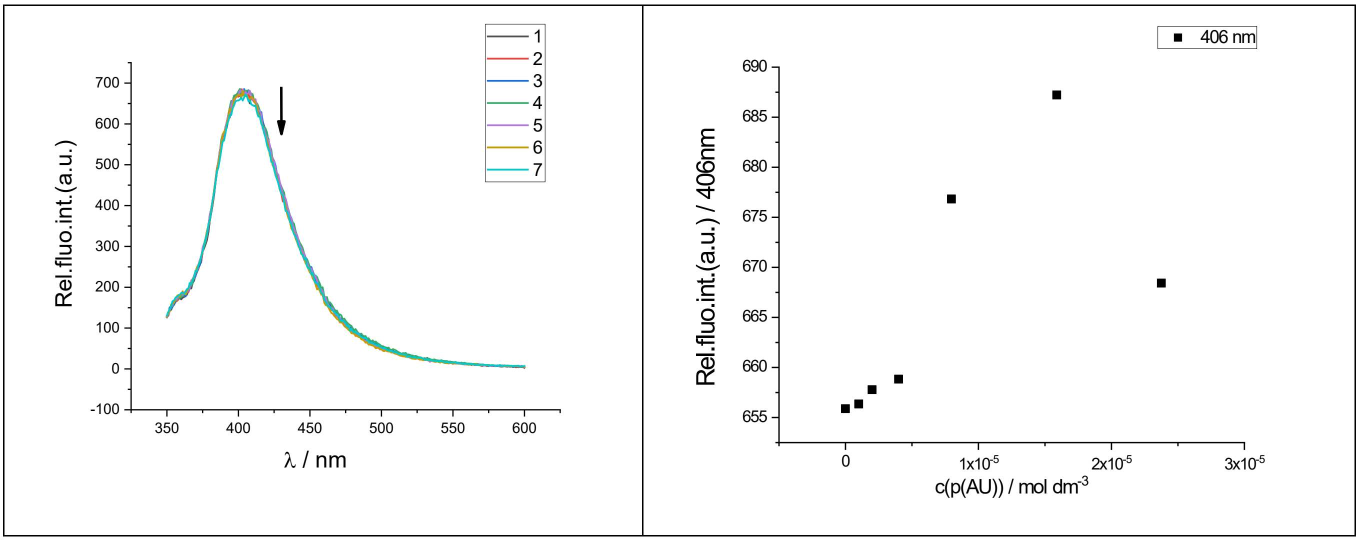


Figure S23. a) Changes in fluorescence spectrum of **19** ($c$= 1.0 × $10^{-6}$ mol $dm^{-3}$, $\lambda_{exc}$=300 nm) upon titration with poly A-poly U ($c$= 1.0 × $10^{-6}$ – 1.8 × $10^{-5}$ mol $dm^{-3}$); b) Dependence of **19** absorbance at $\lambda_{max}$ = 406 nm on c(poly A-poly U), at pH 5.0, sodium cacodylate buffer, $I$ = 0.05 mol $dm^{-3}$.

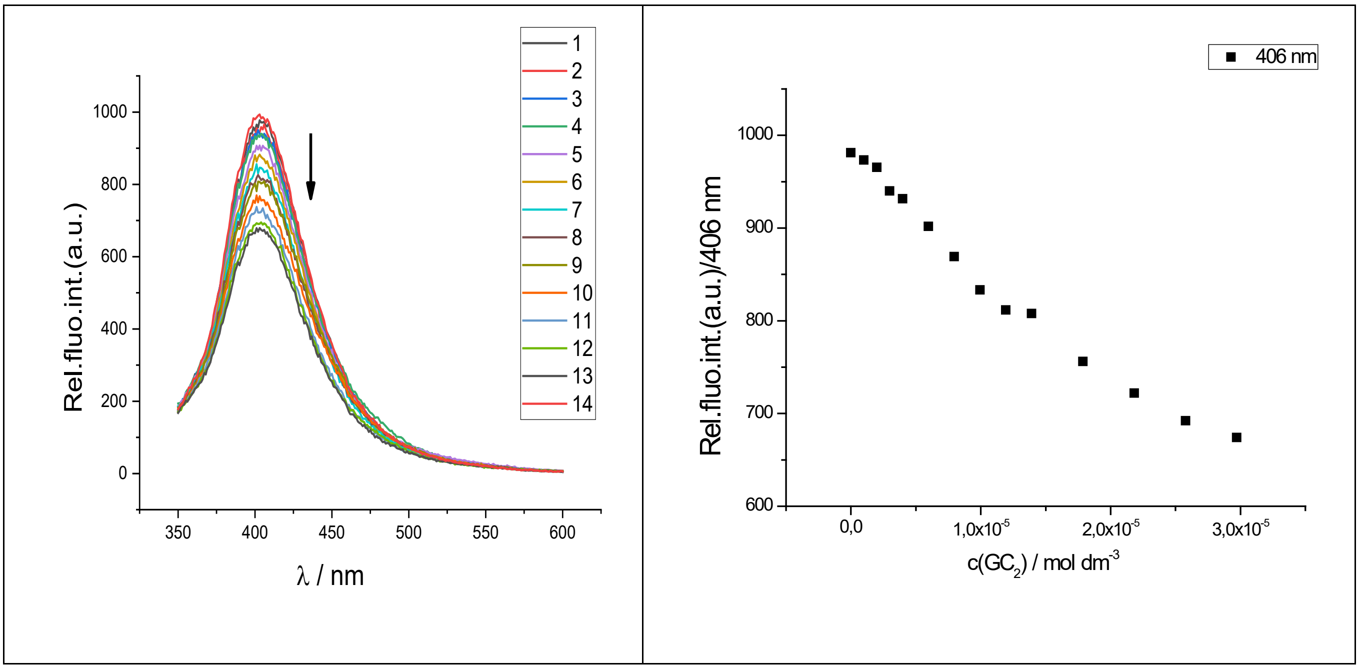


Figure S24. a) Changes in fluorescence spectrum of **19** ($c$= 1.0 × $10^{-6}$ mol $dm^{-3}$, $\lambda_{exc}$=300 nm) upon titration with poly(dGdC)$_2$ ($c$= 1.0 × $10^{-6}$ – 2.9 × $10^{-5}$ mol $dm^{-3}$); b) Dependence of **19** absorbance at $\lambda_{max}$ = 406 nm on c(poly(dGdC)$_2$), at pH 5.0, sodium cacodylate buffer, $I$ = 0.05 mol $dm^{-3}$.

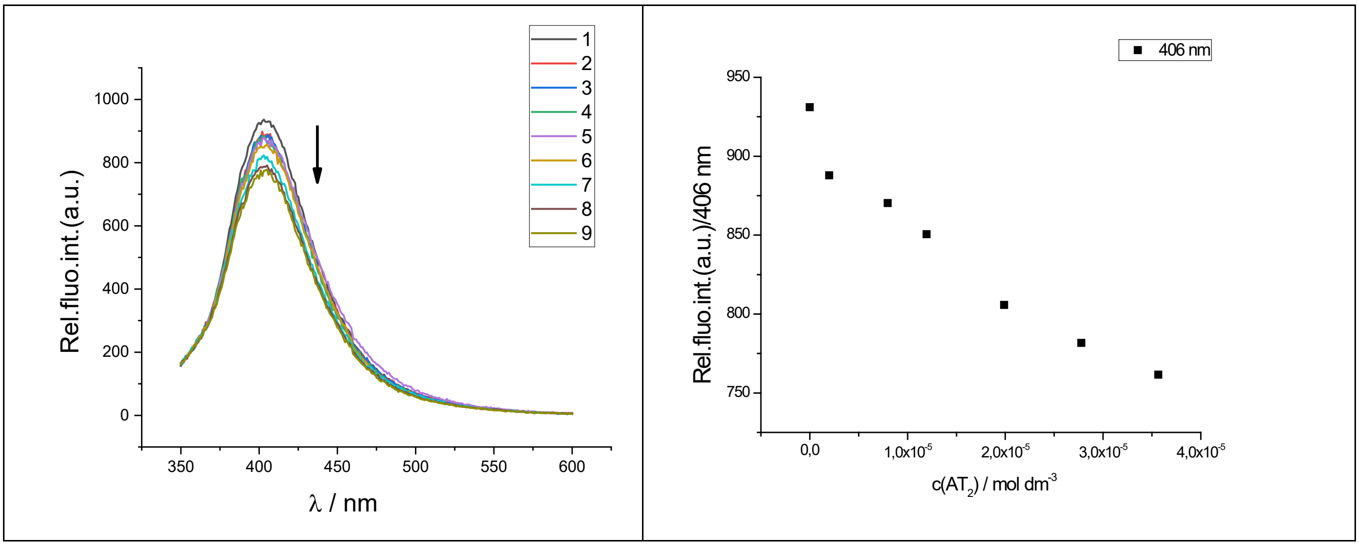


Figure S25. a) Changes in fluorescence spectrum of **19** ($c$= 1.0 × $10^{-6}$ mol $dm^{-3}$, $\lambda_{exc}$=300 nm) upon titration with poly$(dAdT)_2$ ($c$= 1.0 × $10^{-6}$ – 3.6 × $10^{-5}$ mol $dm^{-3}$); b) Dependence of **19** absorbance at $\lambda_{max}$ = 406 nm on c(poly$(dAdT)_2$), at pH 5.0, sodium cacodylate buffer, $I$ = 0.05 mol $dm^{-3}$.

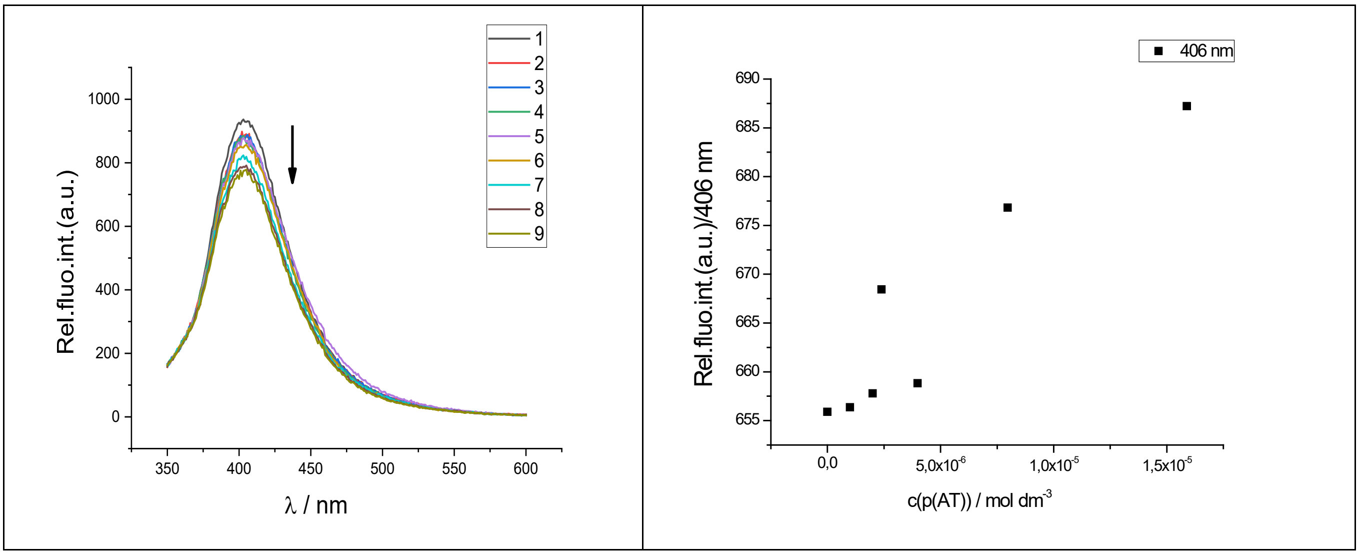


Figure S26. a) Changes in fluorescence spectrum of **19** ($c$= 1.0 × $10^{-6}$ mol $dm^{-3}$, $\lambda_{exc}$=300 nm) upon titration with poly dA-poly dT ($c$= 1.0 × $10^{-6}$ – 3.6 × $10^{-5}$ mol $dm^{-3}$); b) Dependence of **19** absorbance at $\lambda_{max}$ = 406 nm on c(poly dA-poly dT), at pH 5.0, sodium cacodylate buffer, $I$ = 0.05 mol $dm^{-3}$.

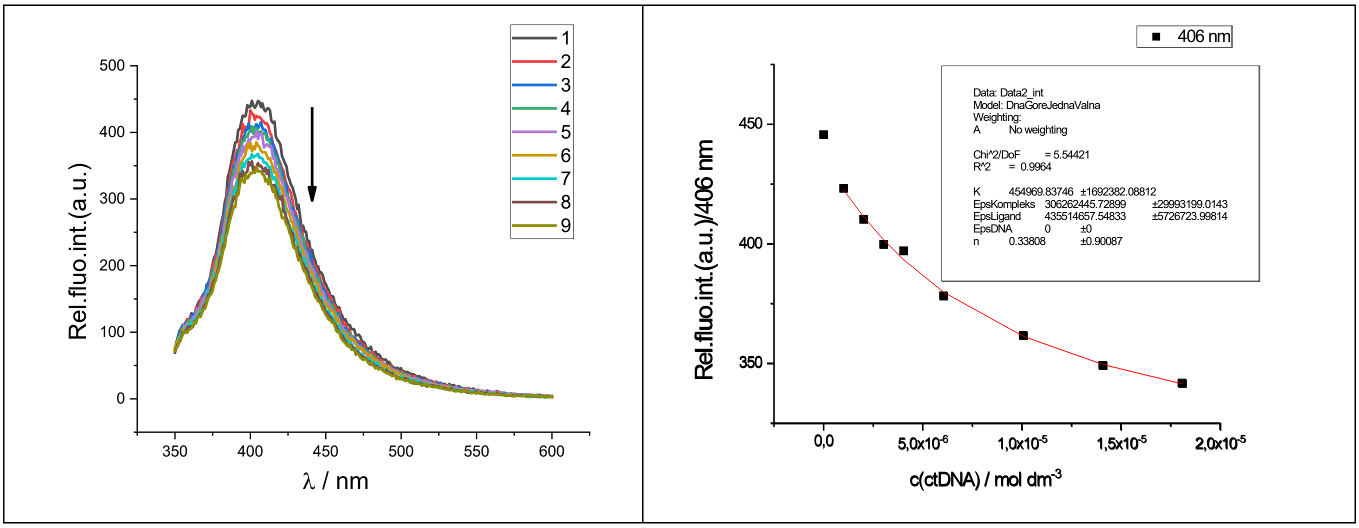


Figure S27. a) Changes in fluorescence spectrum of **22** ($c$= 1.0 × $10^{-6}$ mol $dm^{-3}$, $\lambda_{exc}$=300 nm) upon titration with ctDNA ($c$= 1.0 × $10^{-6}$ – 1.8 × $10^{-5}$ mol $dm^{-3}$); b) Dependence of **22** absorbance at $\lambda_{max}$ = 406 nm on c(ctDNA), at pH 5.0, sodium cacodylate buffer, $I$ = 0.05 mol $dm^{-3}$.

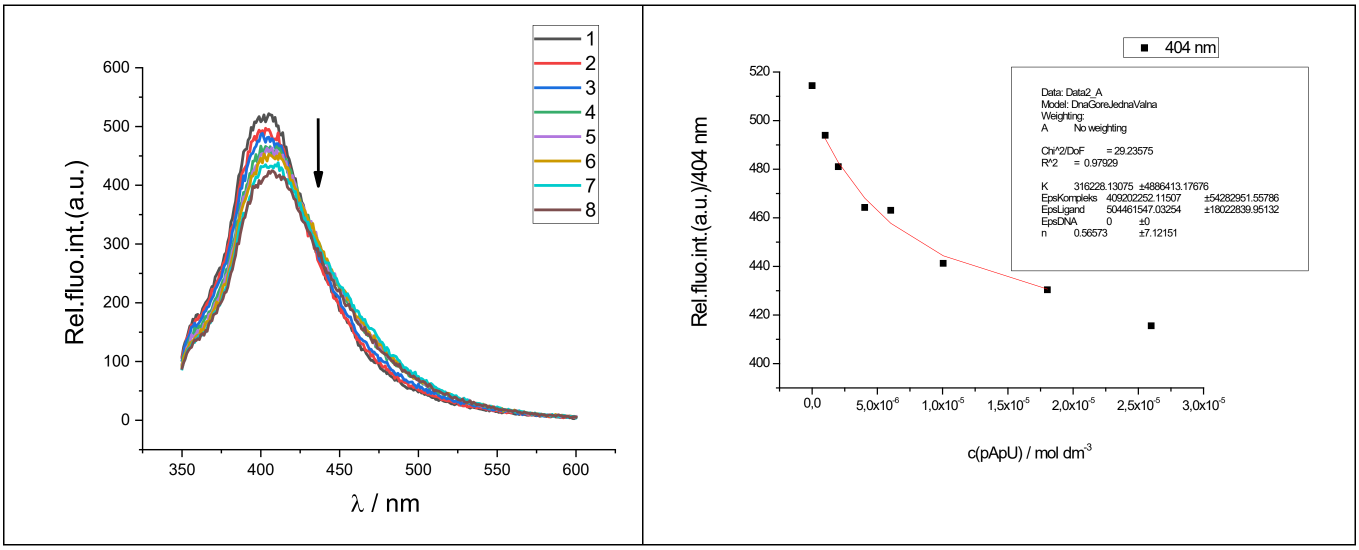


Figure S28. a) Changes in fluorescence spectrum of **22** ($c$= 1.0 × $10^{-6}$ mol $dm^{-3}$, $\lambda_{exc}$=300 nm) upon titration with poly A-poly U ($c$= 1.0 × $10^{-6}$ – 2.6 × $10^{-5}$ mol $dm^{-3}$); b) Dependence of **22** absorbance at $\lambda_{max}$ = 473 nm on c(poly A-poly U), at pH 5.0, sodium cacodylate buffer, $I$ = 0.05 mol $dm^{-3}$.

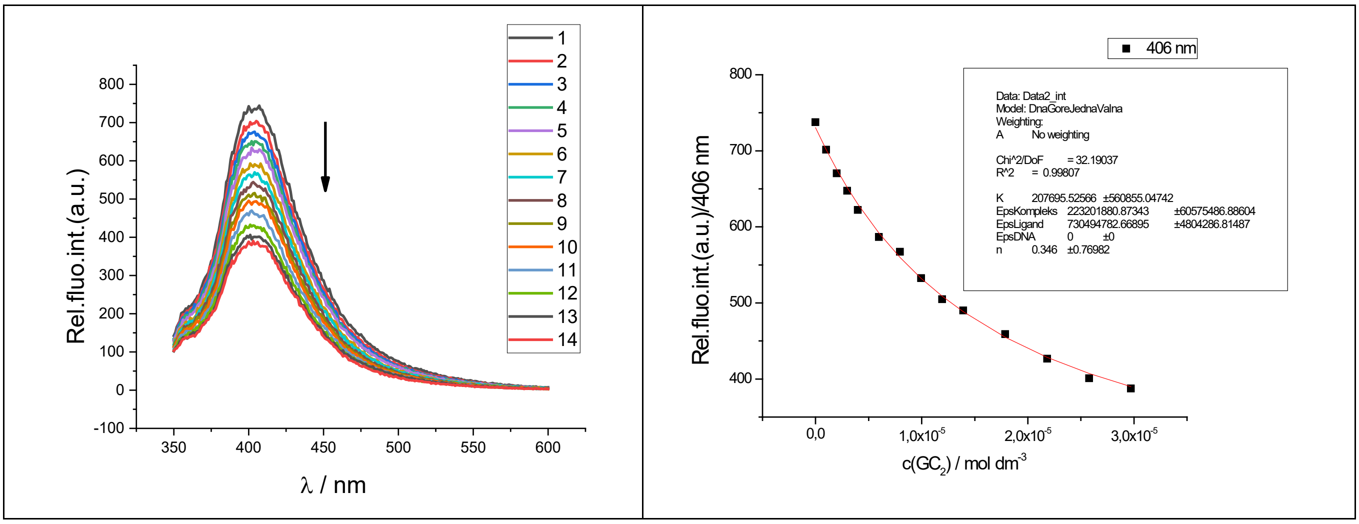


Figure S29. a) Changes in fluorescence spectrum of **22** ($c$= 1.0 × 10$^{-6}$ mol dm$^{-3}$, $\lambda_{exc}$=300 nm) upon titration with poly(dGdC)$_2$ ($c$= 9.9 × 10$^{-7}$ – 2.9 × 10$^{-5}$ mol dm$^{-3}$); b) Dependence of **22** absorbance at $\lambda_{max}$ = 406 nm on c(poly(dGdC)$_2$), at pH 5.0, sodium cacodylate buffer, $I$ = 0.05 mol dm$^{-3}$.

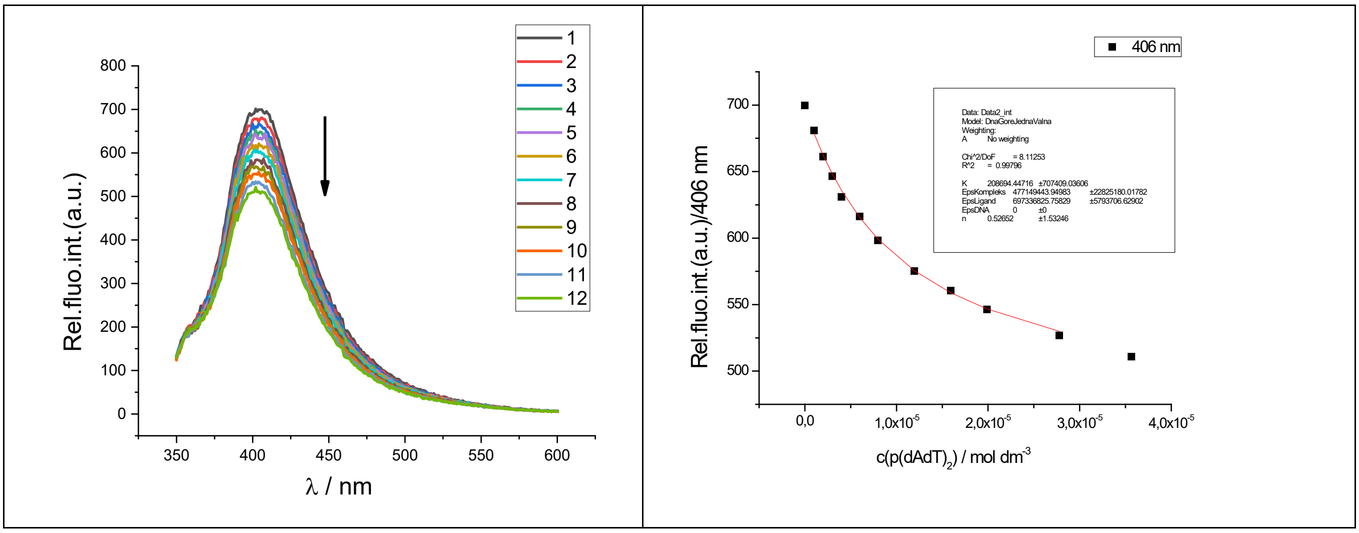


Figure S30. a) Changes in fluorescence spectrum of **22** ($c$= 1.0 × 10$^{-6}$ mol dm$^{-3}$, $\lambda_{exc}$=300 nm) upon titration with poly(dAdT)$_2$ ($c$= 1.0 × 10$^{-6}$ – 3.6 × 10$^{-5}$ mol dm$^{-3}$); b) Dependence of **22** absorbance at $\lambda_{max}$ = 406 nm on c(poly(dAdT)$_2$), at pH 5.0, sodium cacodylate buffer, $I$ = 0.05 mol dm$^{-3}$.

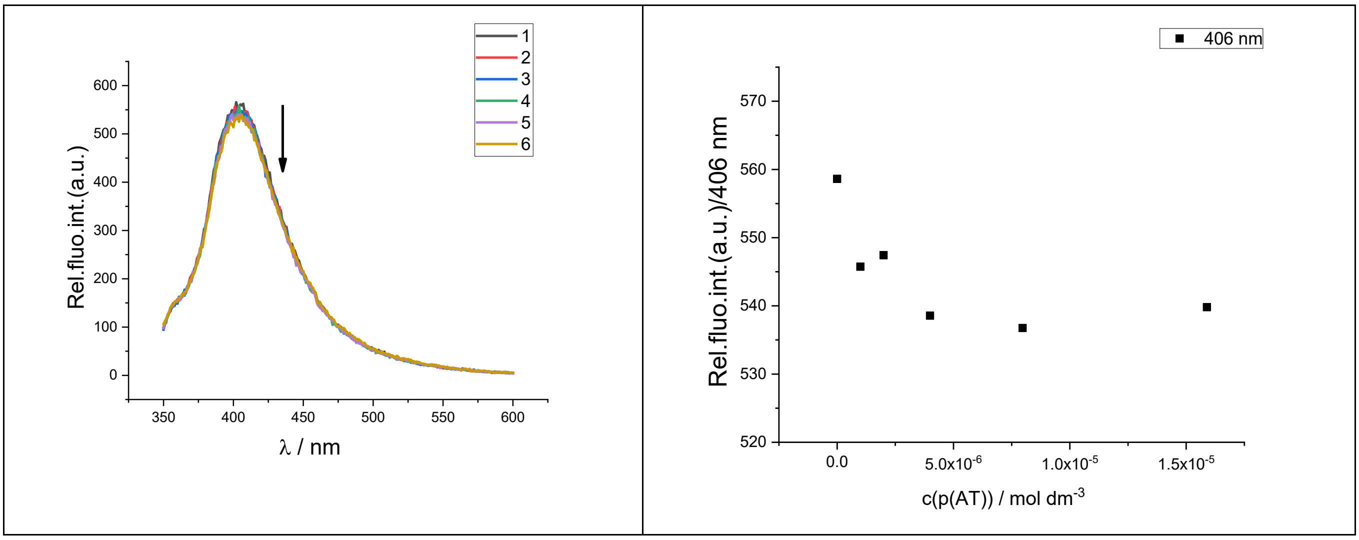


Figure S31. a) Changes in fluorescence spectrum of **22** ($c$= 1.0 × 10$^{-6}$ mol dm$^{-3}$, $\lambda_{exc}$=300 nm) upon titration with poly dA-poly dT ($c$= 1.0 × 10$^{-6}$ – 1.6 × 10$^{-5}$ mol dm$^{-3}$); b) Dependence of **22** absorbance at $\lambda_{max}$ = 406 nm on c(poly dA-poly dT), at pH 5.0, sodium cacodylate buffer, $I$ = 0.05 mol dm$^{-3}$.

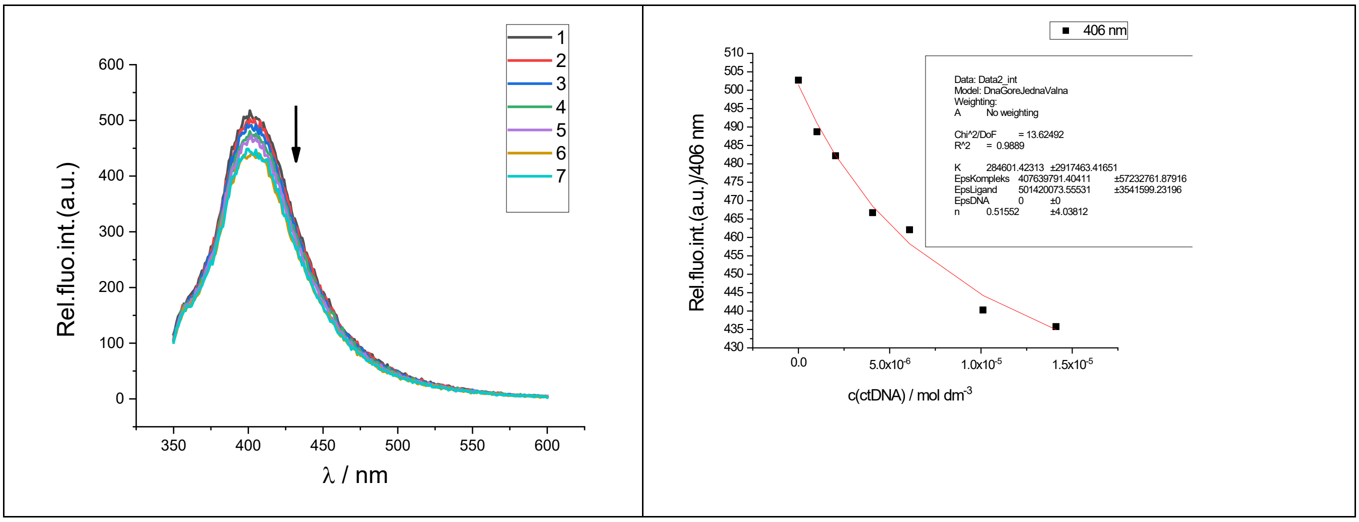


Figure S32. a) Changes in fluorescence spectrum of **23** ($c$= 1.0 × 10$^{-6}$ mol dm$^{-3}$, $\lambda_{exc}$=300 nm) upon titration with ctDNA ($c$= 1.0 × 10$^{-6}$ – 1.4 × 10$^{-5}$ mol dm$^{-3}$); b) Dependence of **23** absorbance at $\lambda_{max}$ = 406 nm on c(ctDNA), at pH 5.0, sodium cacodylate buffer, $I$ = 0.05 mol dm$^{-3}$.

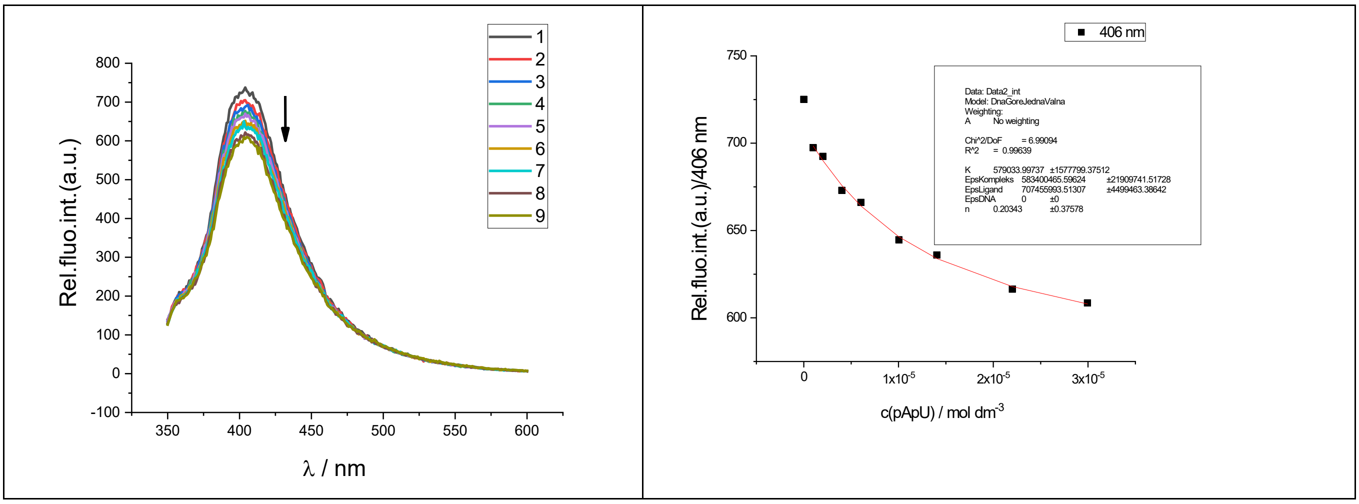


Figure S33. a) Changes in fluorescence spectrum of **23** ($c$= 1.0 × $10^{-6}$ mol $dm^{-3}$, $\lambda_{exc}$=300 nm) upon titration with poly A-poly U ($c$= 1.0 × $10^{-6}$ – 2.99 × $10^{-5}$ mol $dm^{-3}$); b) Dependence of **23** absorbance at $\lambda_{max}$ = 406 nm on c(poly A-poly U), at pH 5.0, sodium cacodylate buffer, $I$ = 0.05 mol $dm^{-3}$.

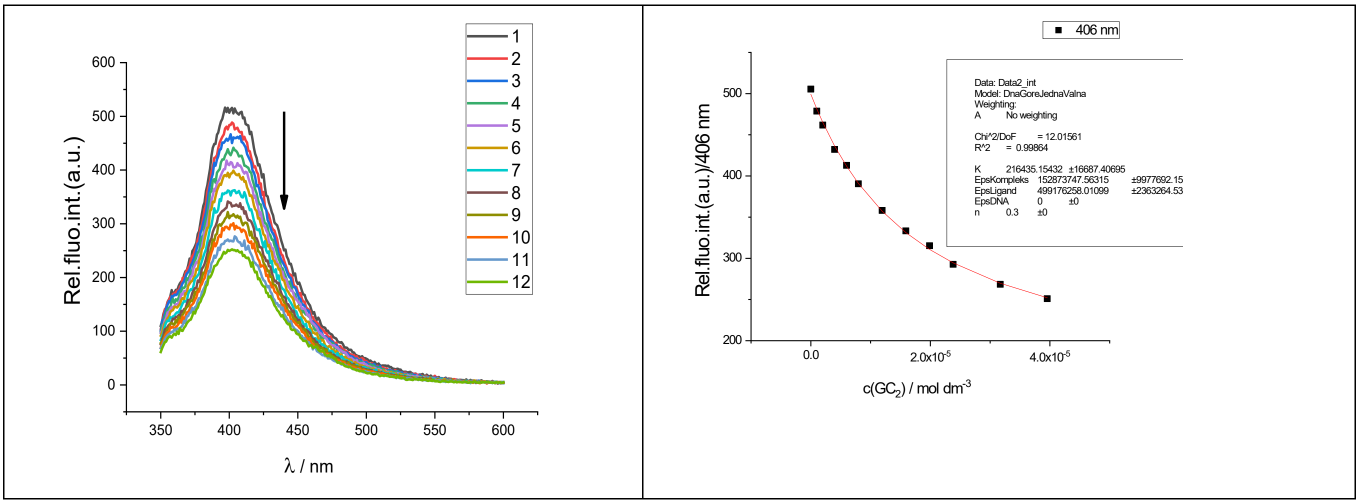


Figure S34. a) Changes in fluorescence spectrum of **23** ($c$= 1.0 × $10^{-6}$ mol $dm^{-3}$, $\lambda_{exc}$=300 nm) upon titration with $poly(dGdC)_2$ ($c$= 1.0 × $10^{-6}$ – 3.96 × $10^{-5}$ mol $dm^{-3}$); b) Dependence of **23** absorbance at $\lambda_{max}$ = 406 nm on c($poly(dGdC)_2$), at pH 5.0, sodium cacodylate buffer, $I$ = 0.05 mol $dm^{-3}$.

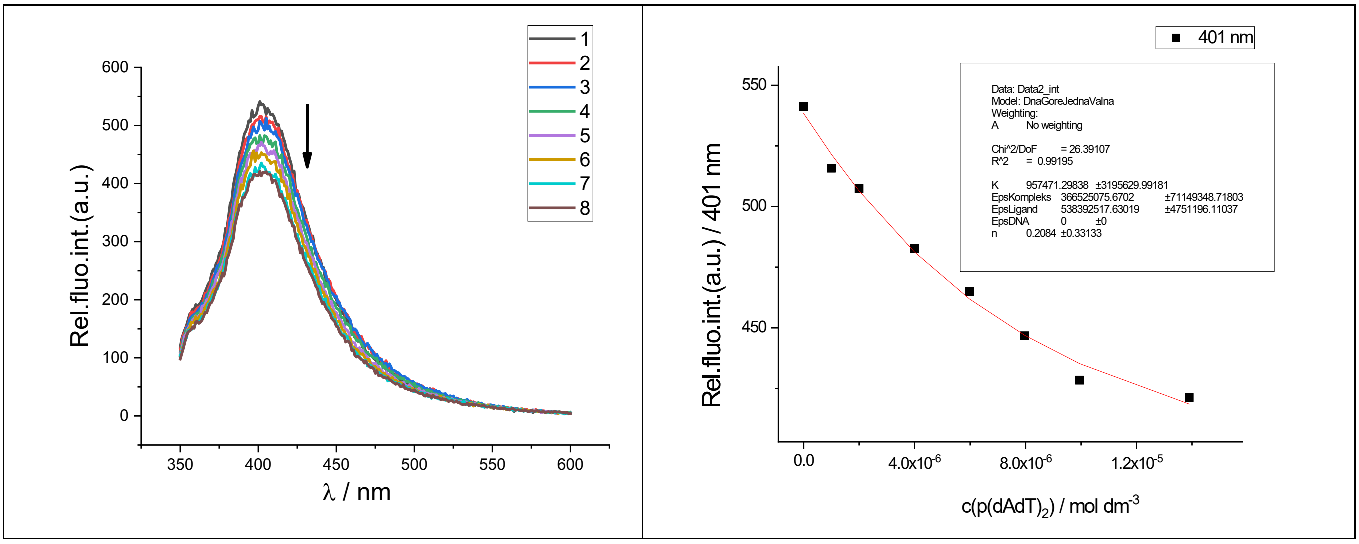


Figure S35. a) Changes in fluorescence spectrum of **23** ($c$= 1.0 × $10^{-6}$ mol $dm^{-3}$, $\lambda_{exc}$=300 nm) upon titration with poly(dAdT)$_2$ ($c$= 1.0 × $10^{-6}$ – 1.39 × $10^{-5}$ mol $dm^{-3}$); b) Dependence of **23** absorbance at $\lambda_{max}$ = 401 nm on c(poly(dAdT)$_2$), at pH 5.0, sodium cacodylate buffer, $I$ = 0.05 mol $dm^{-3}$.

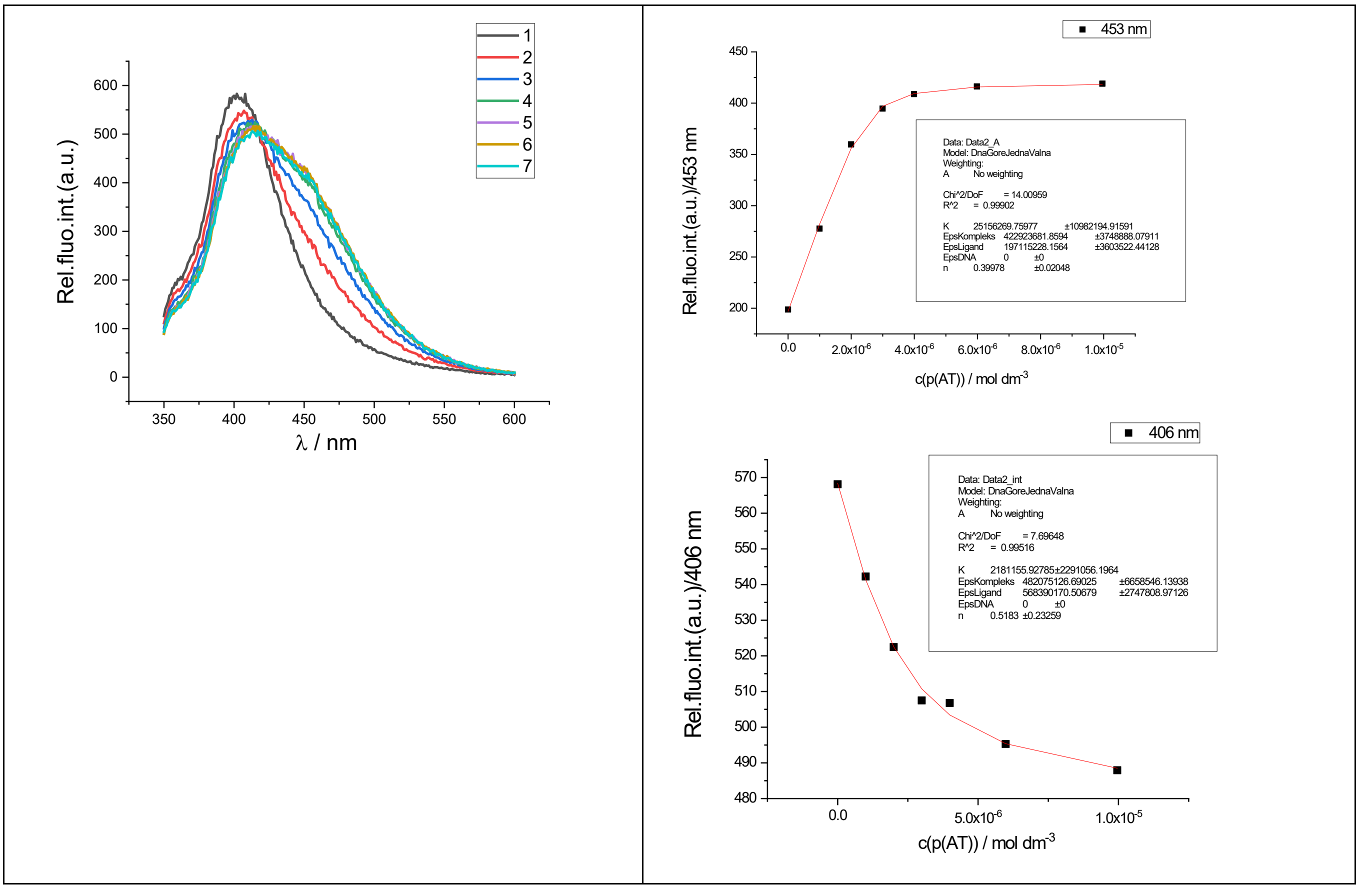


Figure S36. a) Changes in fluorescence spectrum of **23** ($c$= 1.0 × $10^{-6}$ mol $dm^{-3}$, $\lambda_{exc}$=300 nm) upon titration with poly dA-poly dT ($c$= 1.0 × $10^{-6}$ – 9.96 × $10^{-6}$ mol $dm^{-3}$); b) Dependence of **23** absorbance at $\lambda_{max}$ = 406 nm and 453 nm on c(poly dA-poly dT), at pH 5.0, sodium cacodylate buffer, $I$ = 0.05 mol $dm^{-3}$.

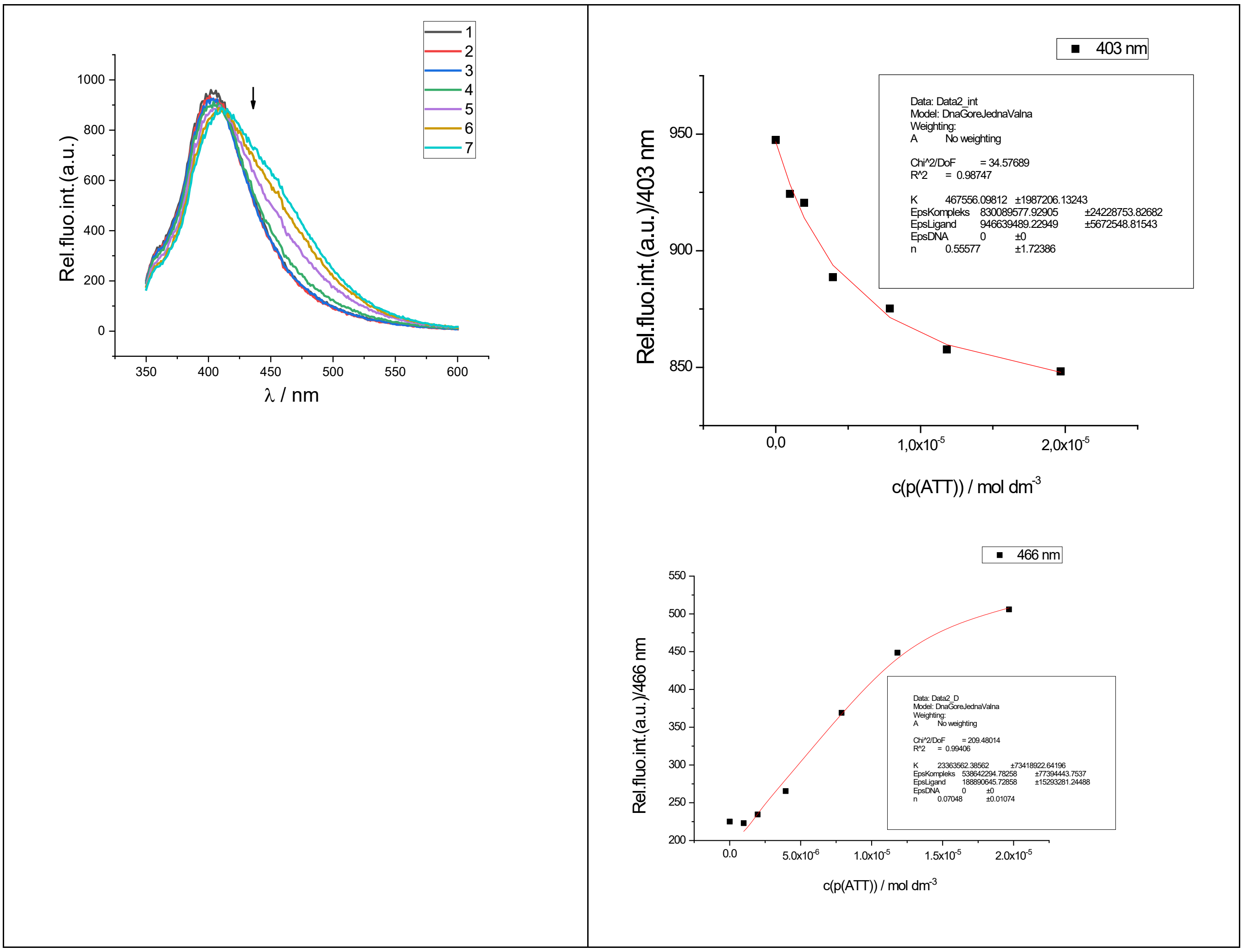


Figure S37. a) Changes in fluorescence spectrum of **23** ($c$= 1.0 × $10^{-6}$ mol $dm^{-3}$, $\lambda_{exc}$=300 nm) upon titration with ATT ($c$= 1.0 × $10^{-6}$ – 1.97 × $10^{-5}$ mol $dm^{-3}$); b) Dependence of **23** absorbance at $\lambda_{max}$ = 403 nm and 466 nm on c(ATT), at pH 5.0, sodium cacodylate buffer, $I$ = 0.05 mol $dm^{-3}$.

## 2.2. Thermal melting experiments

| pH 7.0 | pH 5.0 |
|---|---|
| 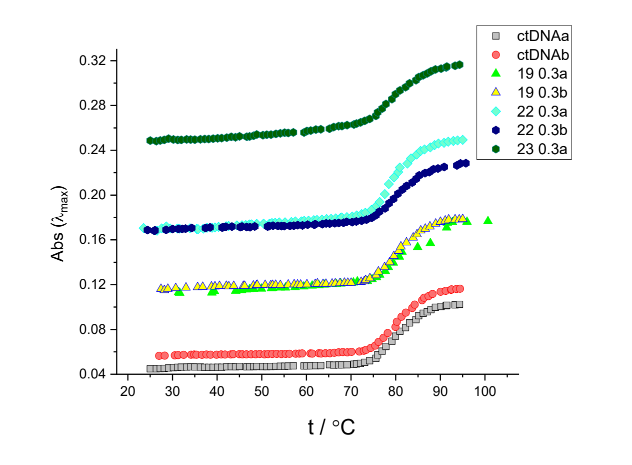 | 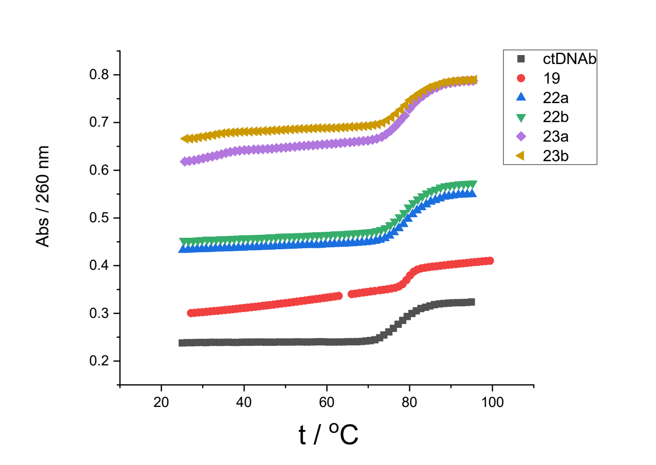 |

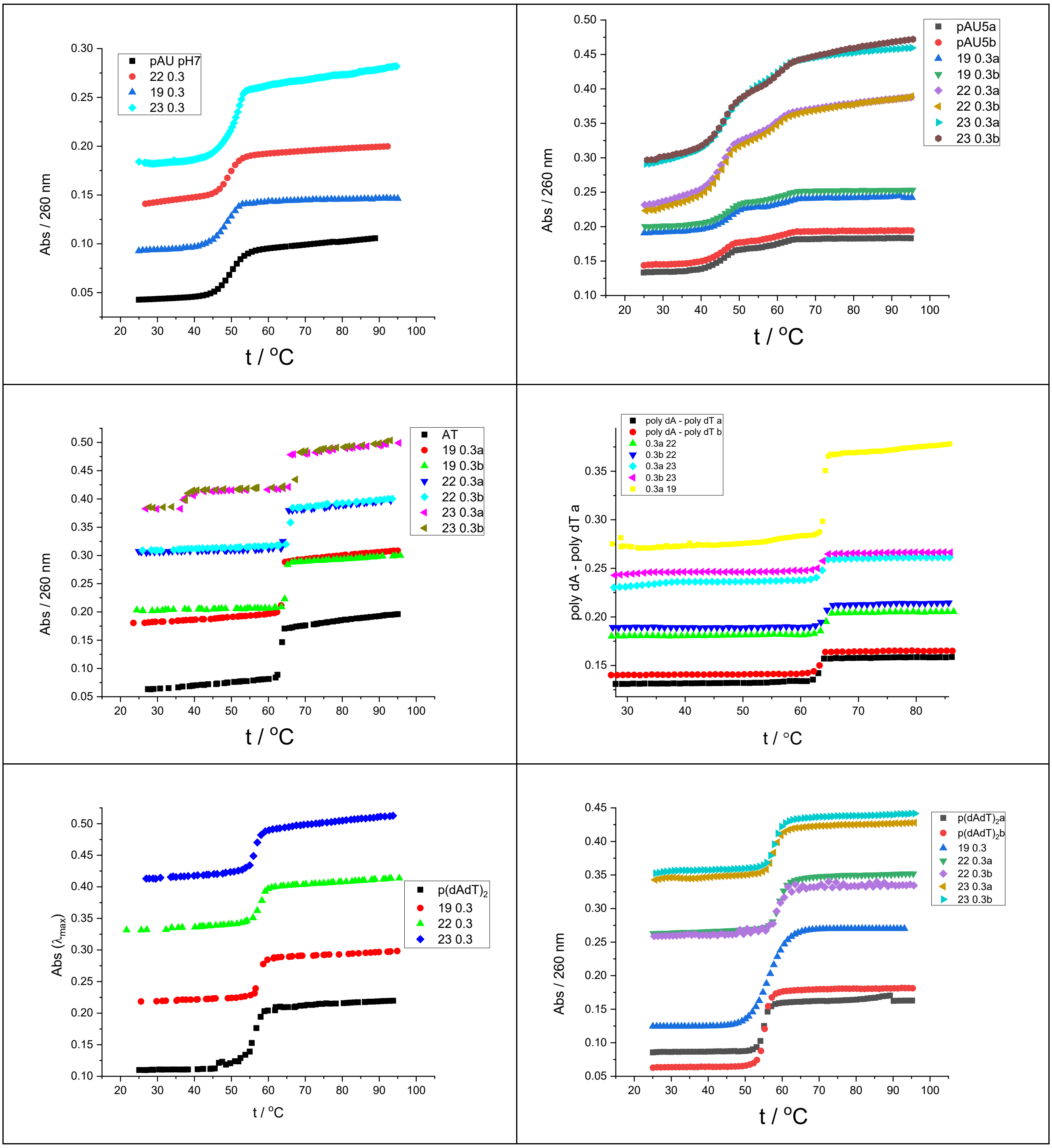


Figure S38. Melting curve of **ctDNA, poly A-poly U, poly dA-poly dT**, **poly (dA-dT)$_2$** upon addition of ratio, ***r*** ( [compound/ [polynucleotide])=0.3 of **19**, **22** and **23** at pH = 7.0 and pH = 5.0 (buffer sodium cacodylate, *I* = 0.05 mol dm$^{-3}$).

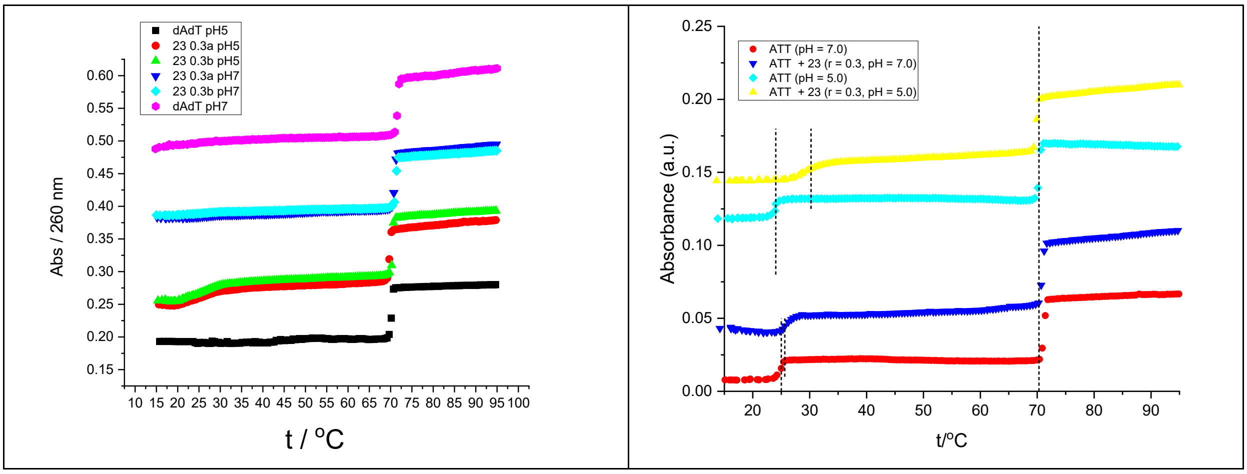


Figure S39. Melting curve of **poly dA-poly dT** and **dAdTdT** upon addition of ratio, ***r*** ( [compound/ [polynucleotide])=0.3 of **23** at pH = 7.0 and pH = 5.0 (buffer sodium cacodylate, *I* = **0.1** mol dm$^{-3}$)

## 2.3. Circular dichroism (CD) experiments

<table>
<tr><th>pH7.0</th><th>ctDNA</th><th>poly(dAdT)$_2$</th><th>poly AT</th></tr>
<tr><td>19</td><td>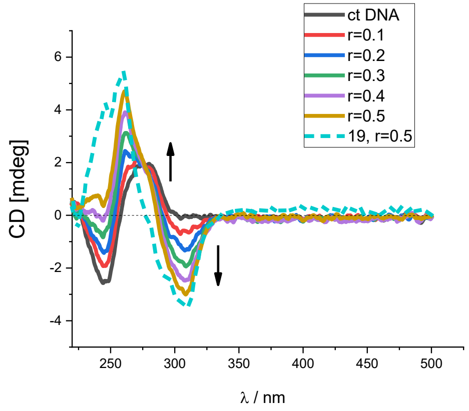
</td><td>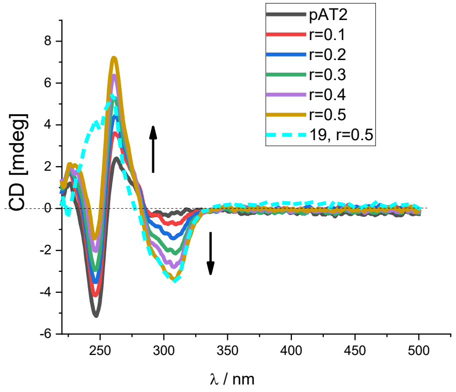
</td><td>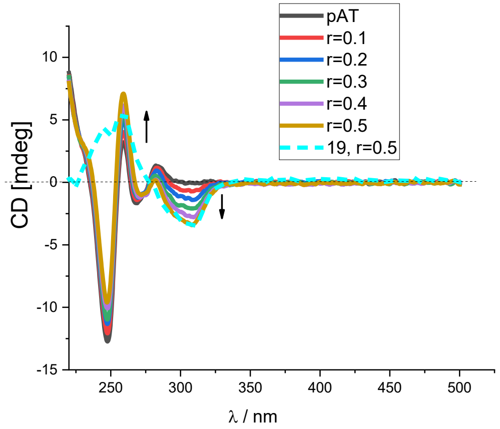
</td></tr>
<tr><td>22</td><td>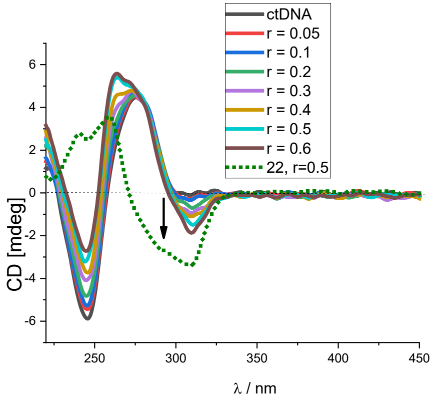
</td><td>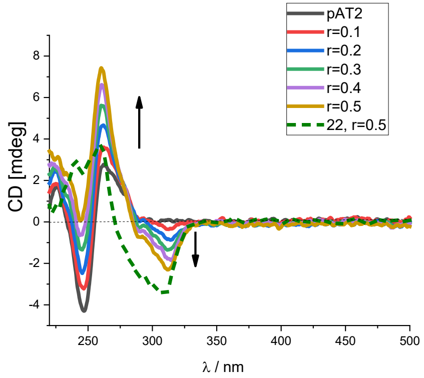
</td><td>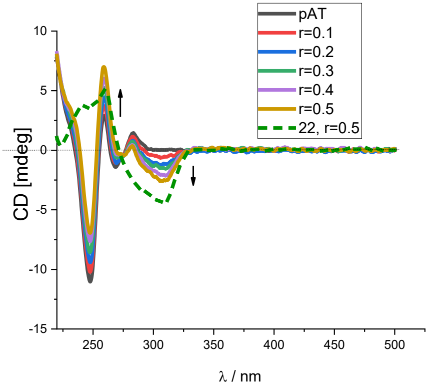
</td></tr>
</table>

| | | | |
|---|---|---|---|
| 23 | 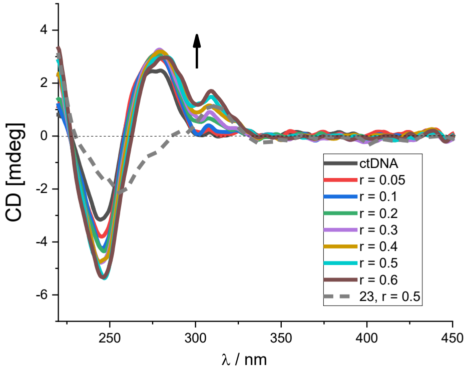 | 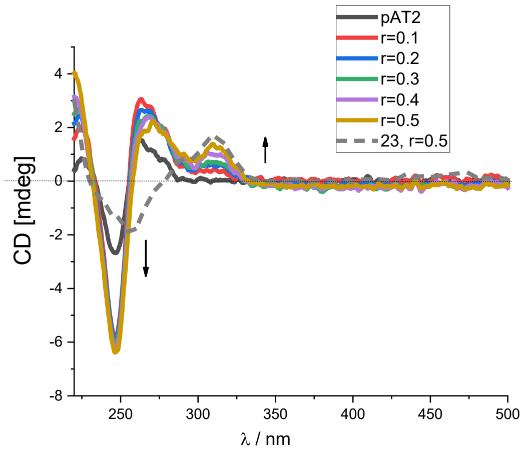 | 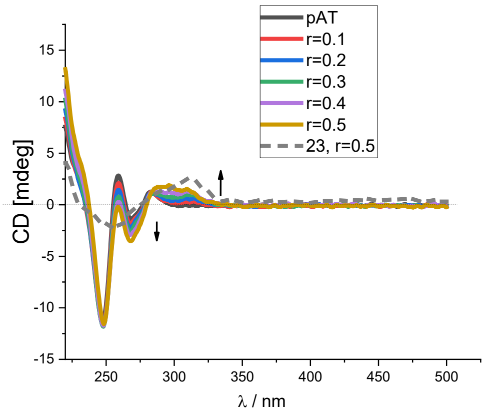 |
| | poly(dGdC)$_2$ | ATT | poly AU |
| 19 | 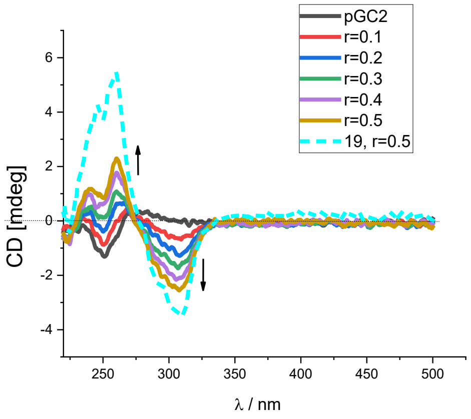 | | 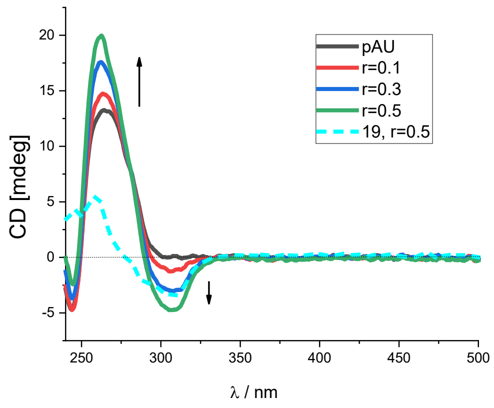 |

22

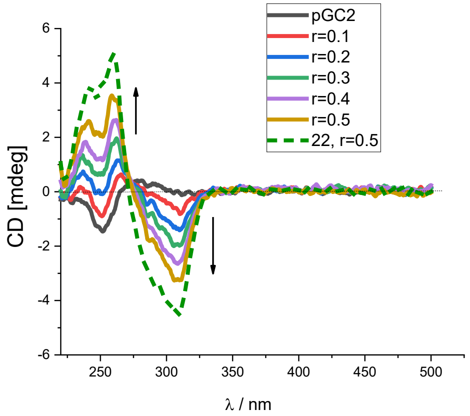
pGC2
r=0.1
r=0.2
r=0.3
r=0.4
r=0.5
22, r=0.5
CD [mdeg]
λ / nm

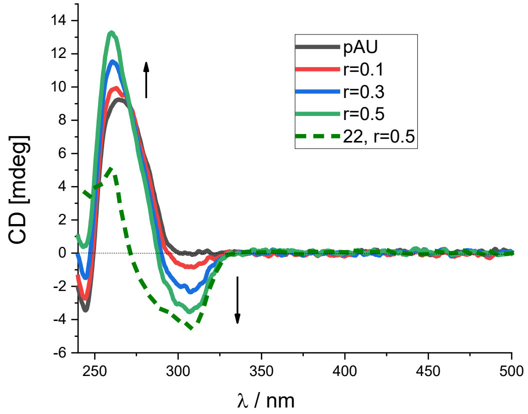
pAU
r=0.1
r=0.3
r=0.5
22, r=0.5
CD [mdeg]
λ / nm

23

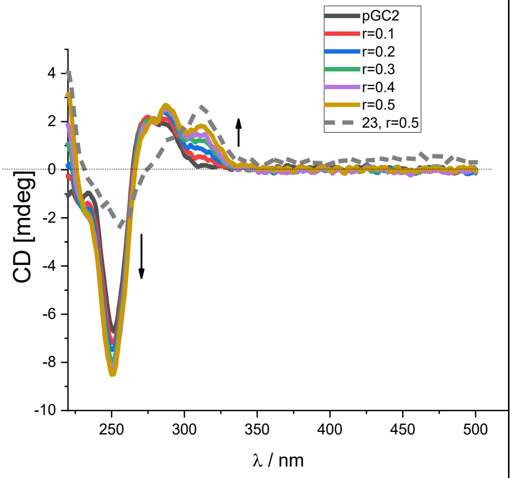
pGC2
r=0.1
r=0.2
r=0.3
r=0.4
r=0.5
23, r=0.5
CD [mdeg]
λ / nm

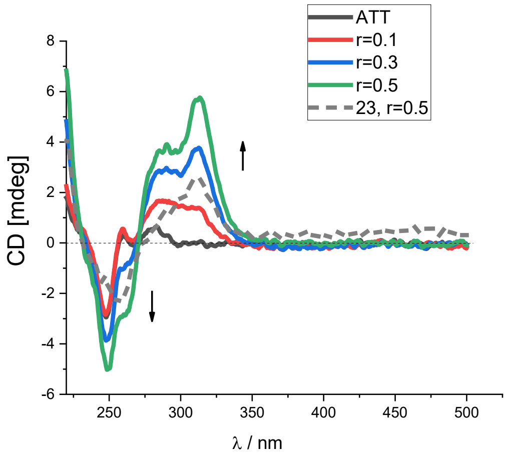
ATT
r=0.1
r=0.3
r=0.5
23, r=0.5
CD [mdeg]
λ / nm

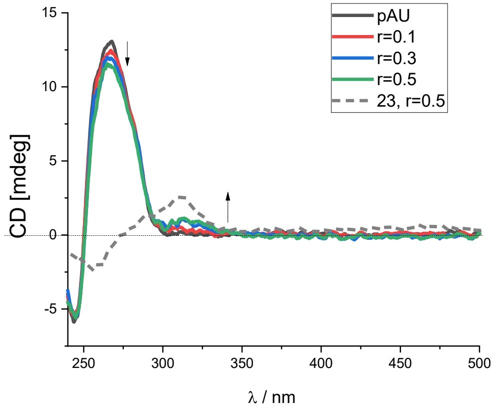
pAU
r=0.1
r=0.3
r=0.5
23, r=0.5
CD [mdeg]
λ / nm

<table>
<tr><th>pH5.0</th><th>ctDNA</th><th>poly(dAdT)$_2$</th><th>poly AU</th></tr>
<tr><td>19</td><td>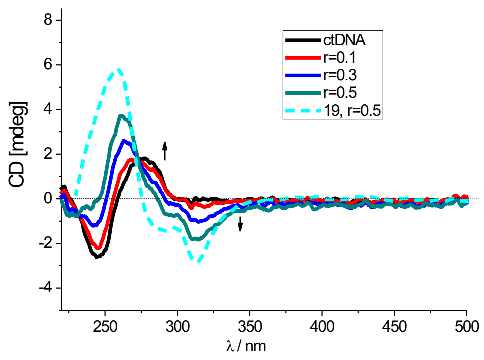
</td><td>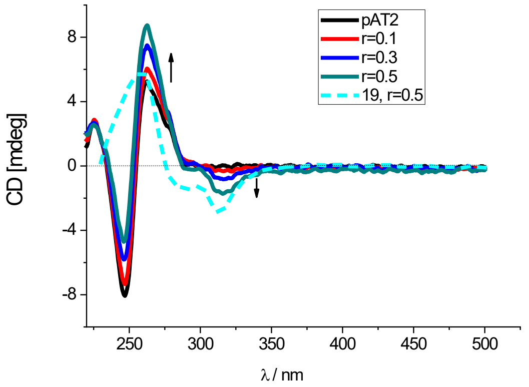
</td><td>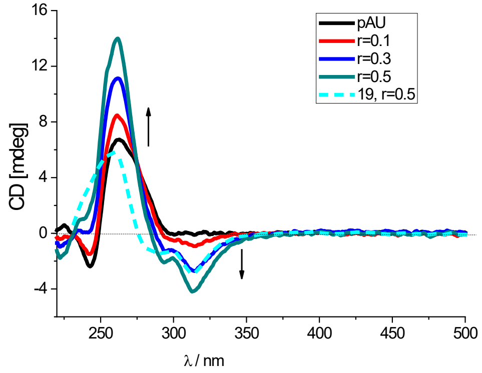
</td></tr>
<tr><td>22</td><td>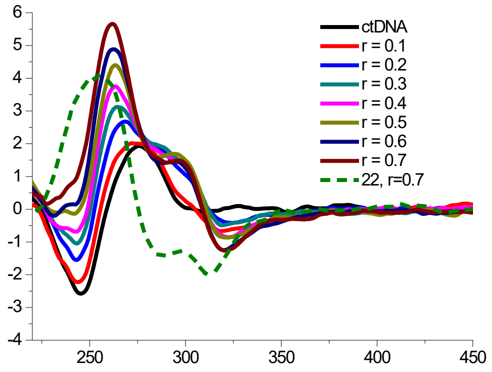
</td><td>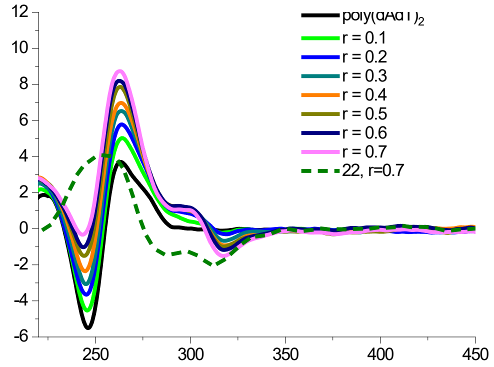
</td><td>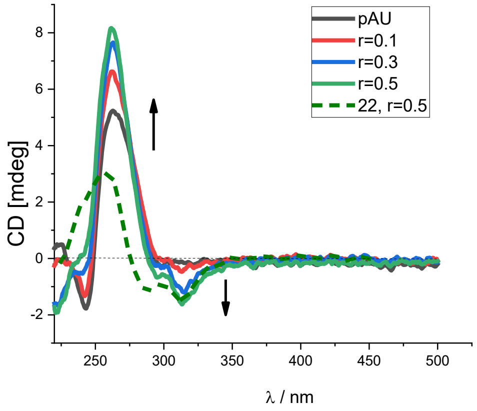
</td></tr>
</table>

| | | | |
|---|---|---|---|
| 23 | 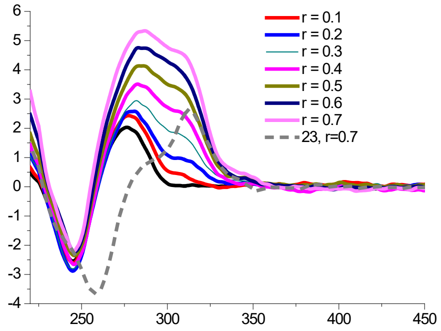 | 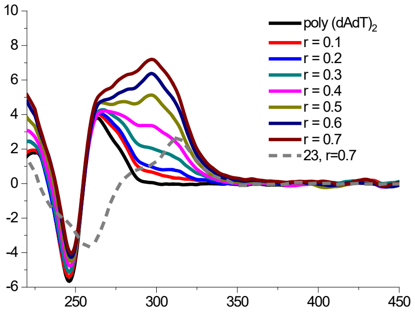 | 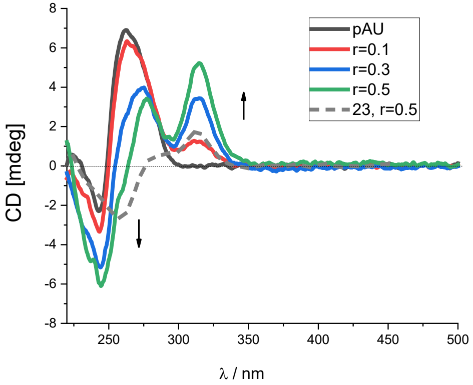 |
| | poly(dGdC)$_2$ | ATT | poly AT |
| 19 | 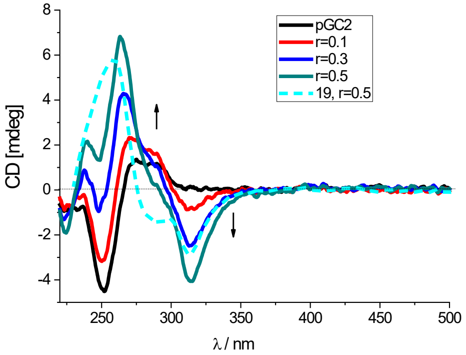 | | 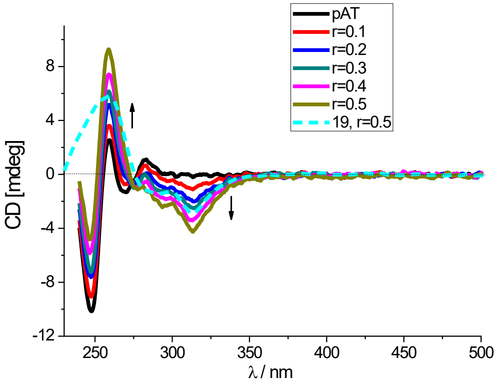 |

| | | | |
|---|---|---|---|
| 22 | 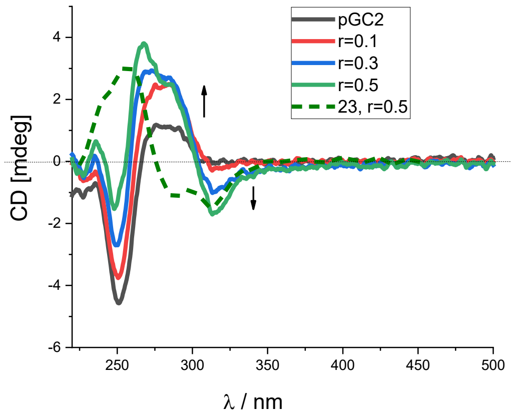 | | 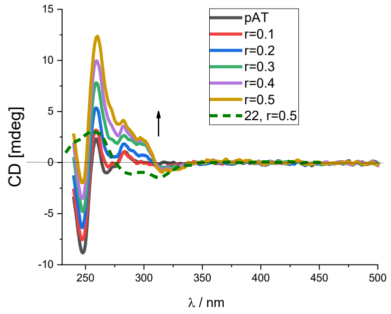 |
| 23 | 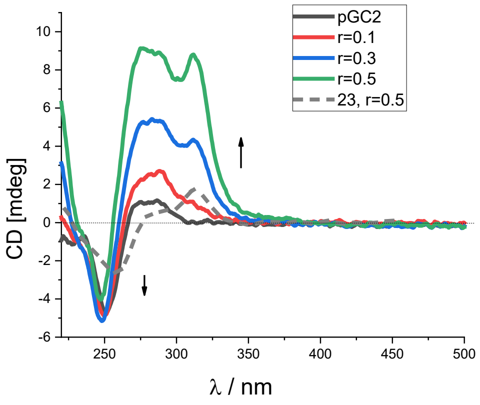 | 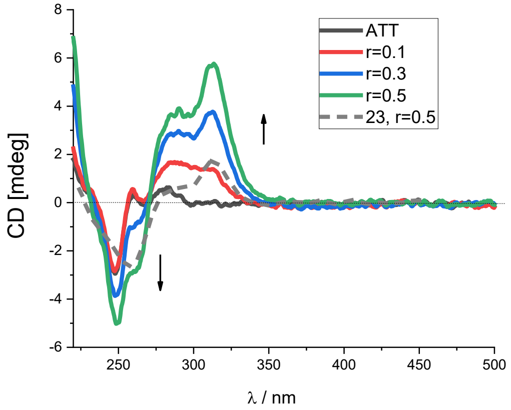 | 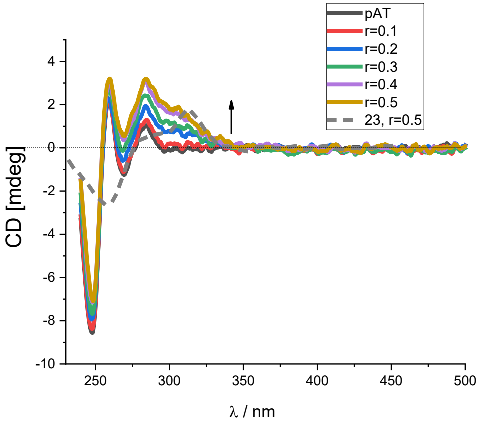 |

Figure S40. CD titration of polynucleotides ($c = 3.0 \times 10^{-5}$ mol dm$^{-3}$) with **19, 22** and **23** at molar ratios ***r*** = [compound] / [polynucleotide] (pH = 7.0 and 5.0, buffer sodium cacodylate, $I$ = 0.05 mol dm$^{-3}$).

## 2.4. Mass spectra

## 2.4. Mass spectra

Figure S41 **a.** Snapshot scan of compound **19** (signal at *m/z 587* assigned as [**19**+H]$^+$ ion) dissolved in $H_2O$ or $D_2O$ about $10^{-5}$M. The observed signals correspond to the H/D exchange results observed for the single charged ion of compound **19**.

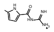

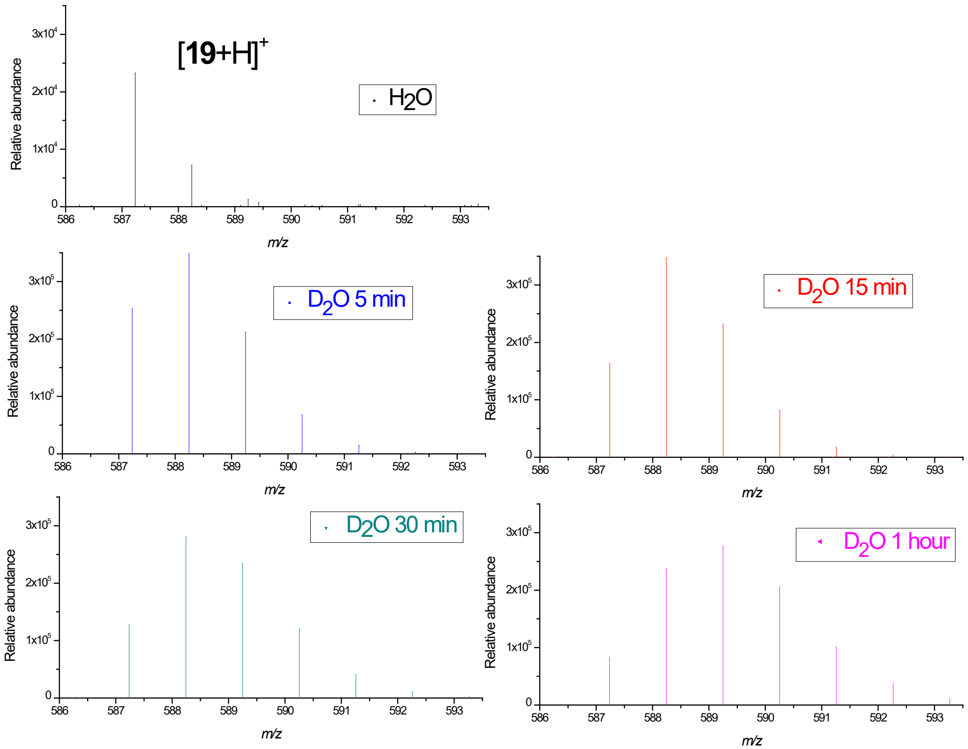

Figure S41 **b.** Snapshot scan of compound **19** (signal at *m/z 294* assigned as [**19**+2H]$^{2+}$ ion) dissolved in $H_2O$ or $D_2O$ about $10^{-5}$M. The observed signals correspond to the H/D exchange results observed for the double charged ion of compound **19.**

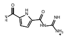

19

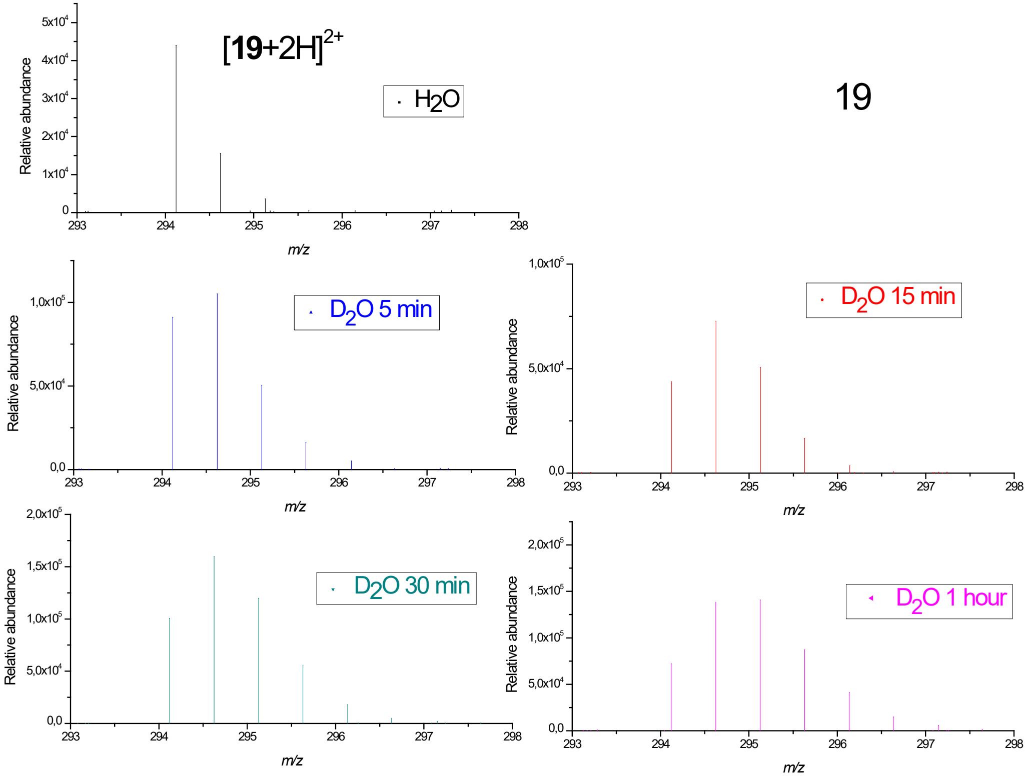

Figure S41 **c.** Snapshot scan of compound **22** (signal at *m/z 658* assigned as [**22**+H]$^+$ ion) dissolved in $H_2O$ or $D_2O$ about $10^{-5}$M. The observed signals correspond to the H/D exchange results observed for the single charged ion of compound **22**.

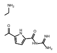

22

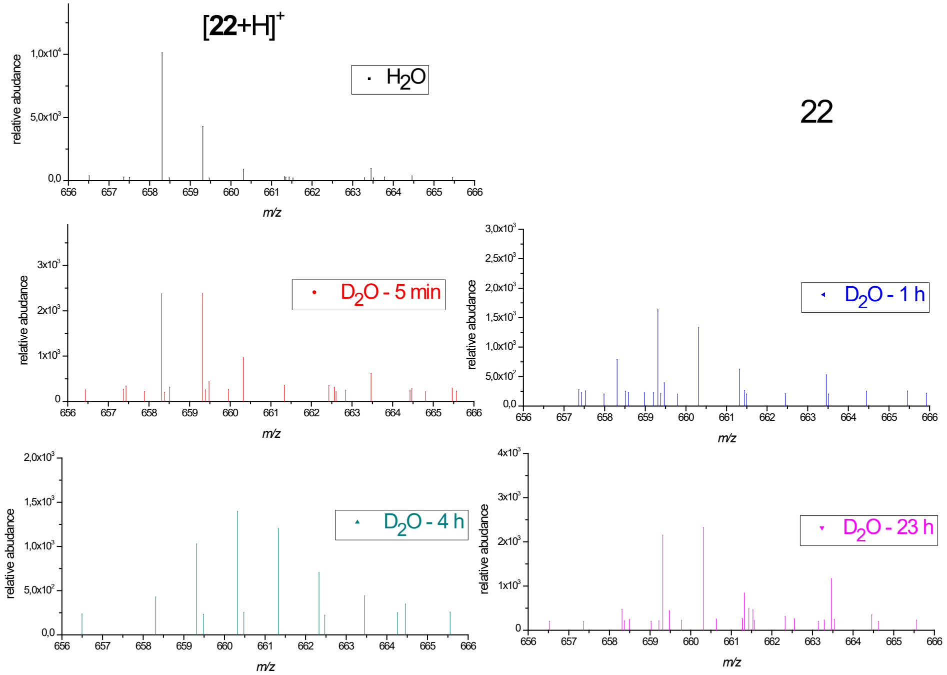

Figure S41 **d.** Snapshot scan of compound **22** (signal at *m/z 329* assigned as [**22**+2H]$^{2+}$ ion) dissolved in $H_2O$ or $D_2O$ about $10^{-5}$M. The observed signals correspond to the H/D exchange results observed for the double charged ion of compound **22.**

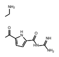

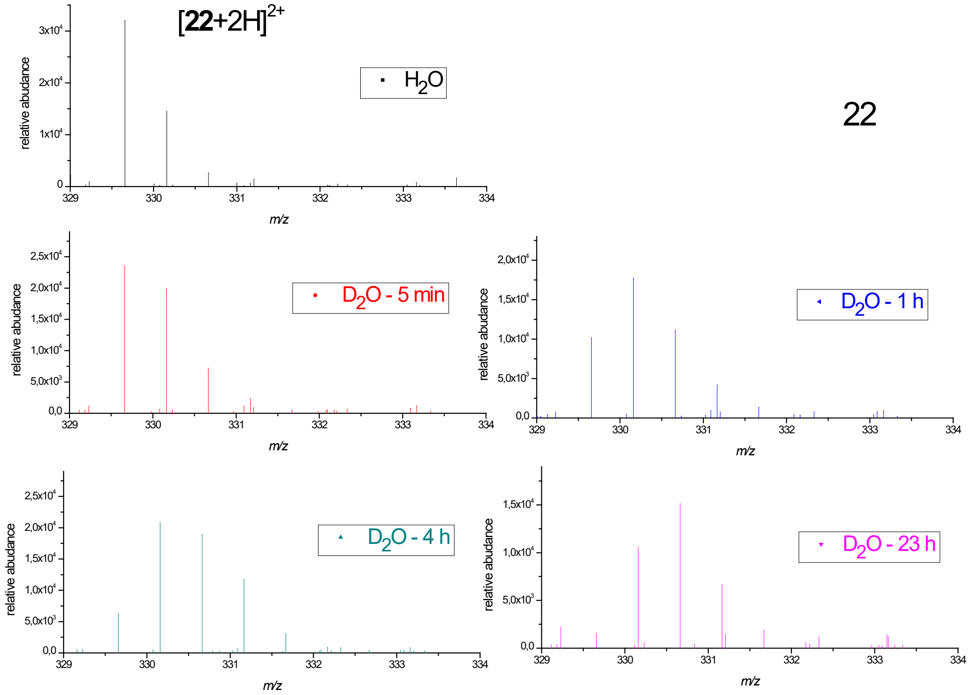


22

Figure S41 **e.** Snapshot scan of compound **23** (signal at *m/z 658* assigned as [**23**+H]$^+$ ion) dissolved in $H_2O$ or $D_2O$ about $10^{-5}$M. The observed signals correspond to the H/D exchange results observed for the single charged ion of compound **23**.

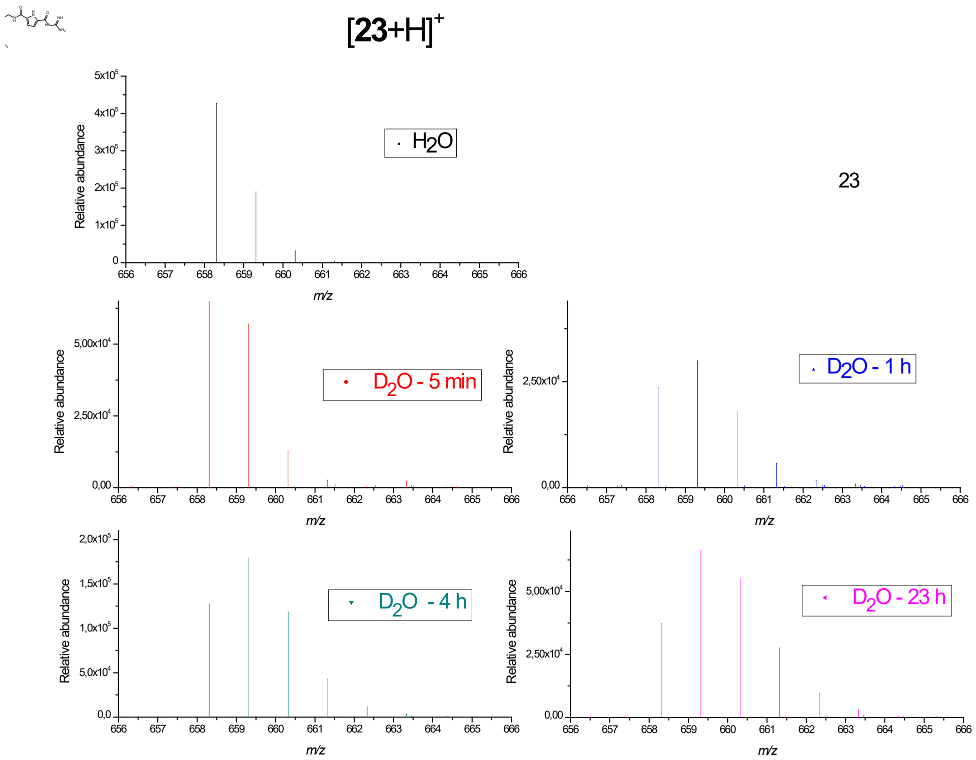

Figure S41 **f.** Snapshot scan of compound **23** (signal at *m/z 329* assigned as [**23**+2H]$^{2+}$ ion) dissolved in $H_2O$ or $D_2O$ about $10^{-5}$M. The observed signals correspond to the H/D exchange results observed for the double-charged ion of compound **23.**

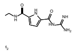

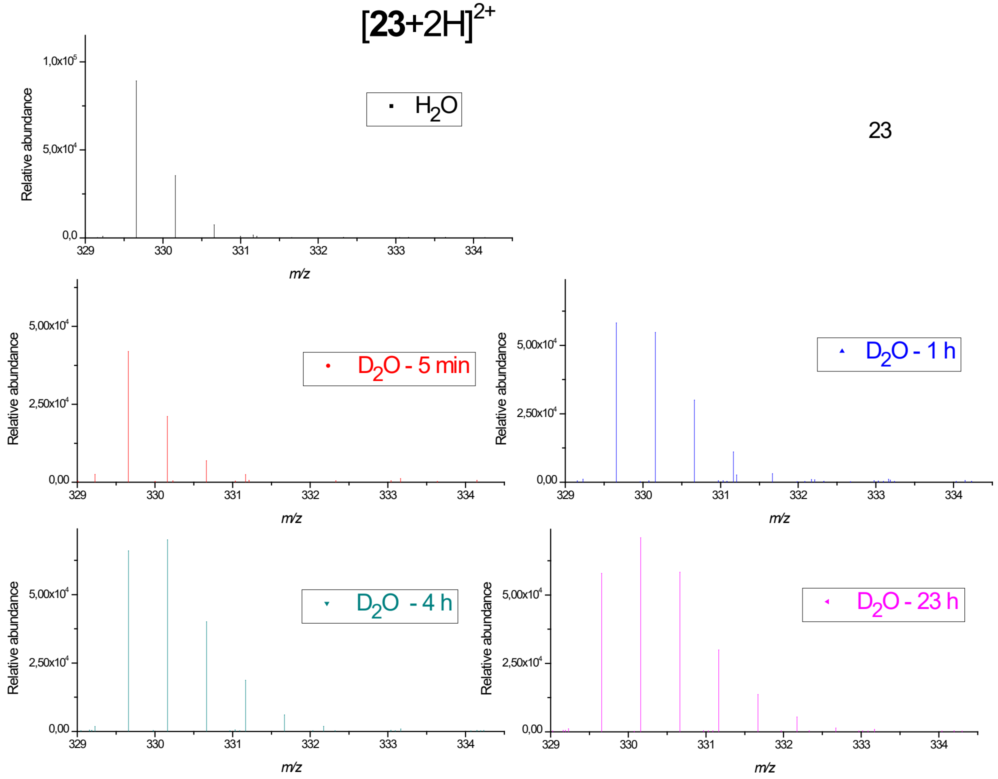


23

Figure S41 **g**. Snapshot scan of compound **23** (signal at *m/z 1337* assigned as [$\mathbf{23_2}$+Na]$^+$ ion) dissolved in $H_2O$ or $D_2O$ about $10^{-5}$M. The observed signals correspond to the H/D exchange results observed for the single charged dimer ion (sodium adduct) of compound **23**.

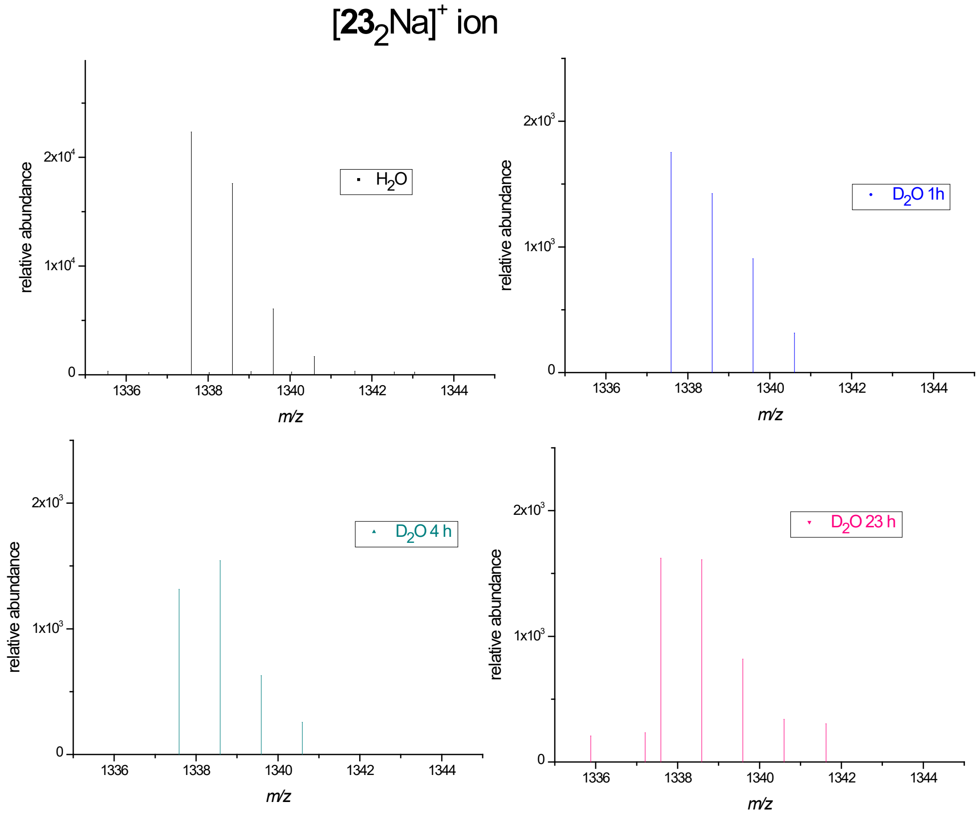

## 3. Computational analysis of 19, 22 and 23

| System | pH = 7 | pH = 5 |
|---|---|---|
| **19** | 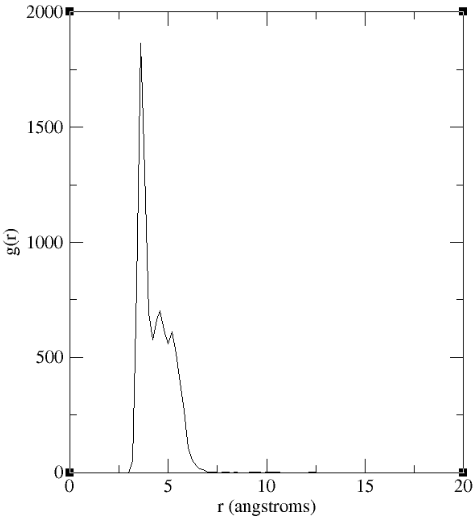 | 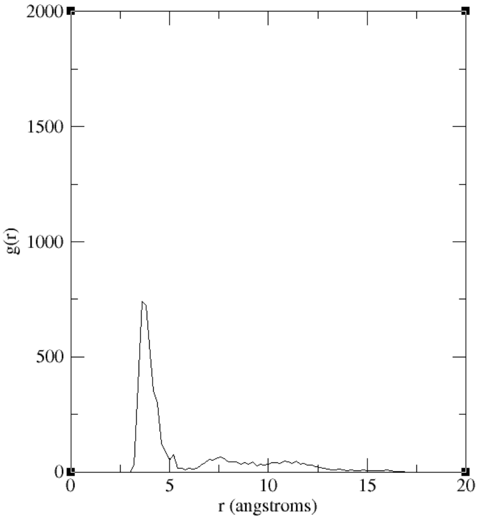 |
| **22** | 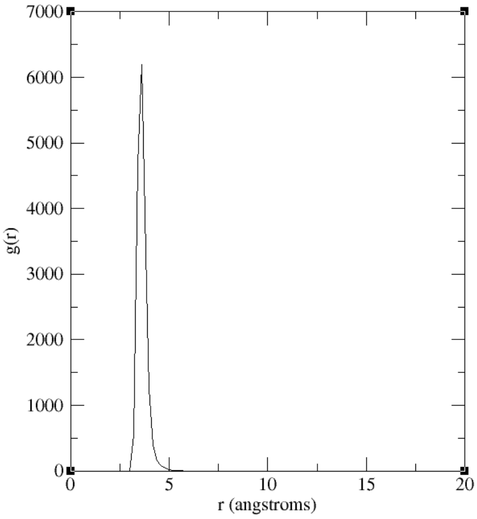 | 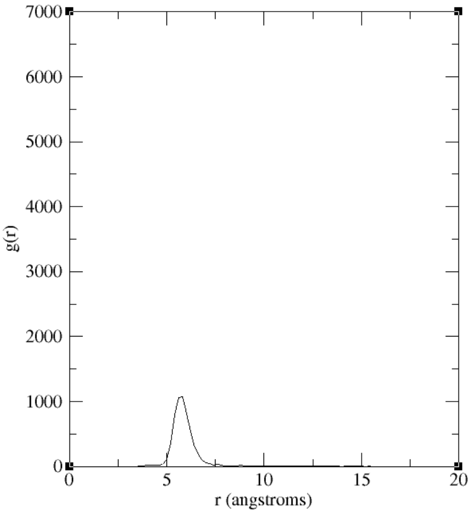 |
| **23** | 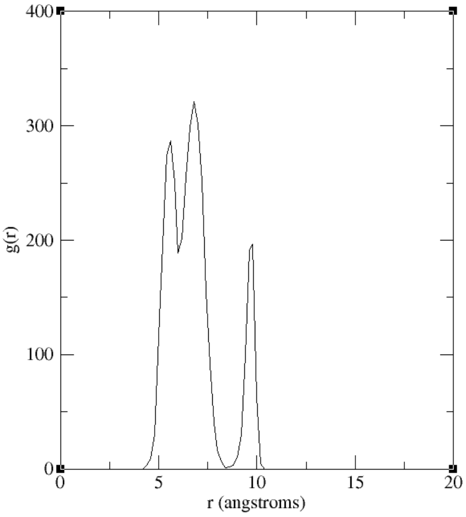 | 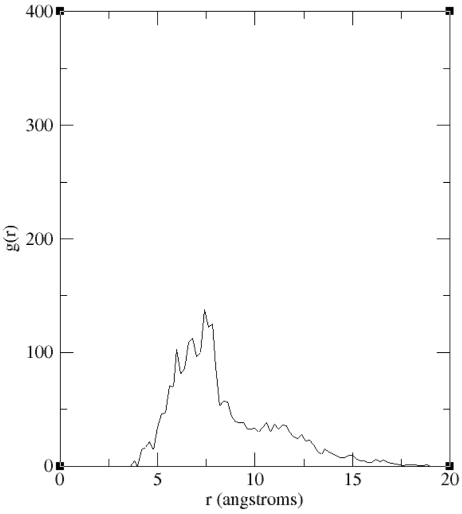 |

Figure S42. RDF graphs describing the distance between centres of mass of phenanthridine and pyrrole rings during the last 30 ns of the matching molecular dynamics simulations.

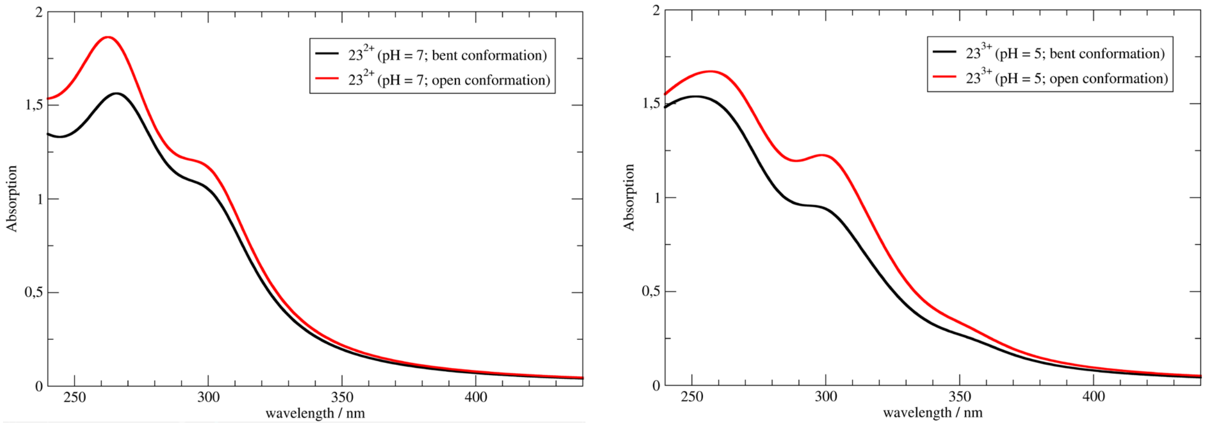


Figure S43. Calculated UV/Vis spectra for the representative structures of both bent and open conformations of systems **23** under normal (pH = 7, left) and acidic conditions (pH = 5, right) obtained with the TD-DFT approach and the (SMD)/B3LYP/6–31+G(d) model in water.
